# Development of Benzothiophene–Bearing Bis–oxadiazole Derivatives for Potential Anticancer Targets: Evaluation of DFT, ADME–Tox, and In Silico Docking

**DOI:** 10.1002/cbdv.71782

**Published:** 2026-10-09

**Authors:** Nagaraju Madala, Vishal Sharma, Srilalitha Vinnakota

**Affiliations:** ^1^ Centre For Chemical Sciences and Technology, University College of Engineering Science and Technology Hyderabad Jawaharlal Nehru Technological University Hyderabad Hyderabad Telangana India; ^2^ Medicinal Chemistry Division, VSN LABS IDA Cherlapally, Phase‐V Hyderabad Telangana India; ^3^ Discovery Chemistry Division Jubilant Biosys Ltd Knowledge Park II, Greater Noida Uttar Pradesh India; ^4^ Department of Chemistry, Faculty of Science and Technology ICFAI Foundation for Higher Education Hyderabad Telangana India

**Keywords:** ADMET lab 3.0, anticancer, benzothiophene, Bis‐oxadiazole, DFT, in silico techniques

## Abstract

Combining two pharmacophores into one molecule with multiple applications presents interest in the field of medicinal chemistry. Benzo[*b*]thiophenes are among privileged scaffolds due to their wide biological activities, whereas oxadiazoles are versatile linkers with pharmacological potential. Thus, this article focused on the synthesis of novel benzo[*b*]thiophene‐linked bis‐oxadiazoles and the exploration of their anticancer potential. The structural elucidation of the prepared oxadiazole compounds was established by ^1^H NMR, ^13^C NMR, and HRMS spectroscopic analysis. The in vitro anticancer activity effect on the human colorectal (HT‐29, HCT‐116 and Caco‐2) and breast (MDA‐MB‐231) cancer cell lines was assessed for each synthesized compound. It was revealed that compounds **7b** and **7g** showed well‐established anticancer affinities on HT‐29 and Caco‐2 cell lines with inhibitory concentrations of 6.2 ± 0.67 and 8.1 ± 0.89 µg/mL in comparison to the reference drug etoposide (IC_50_ = 7.5 ± 1.04 and 9.5 ± 1.01 µg/mL). Notably, compound **7i** displayed the highest antiproliferative activity among the tested compounds, with selectivity towards the MDA‐MB‐231, IC_50_ 9.5 ± 0.38 µg/mL, with respect to Etoposide (IC_50_ = 8.4 ± 0.91 µg/mL). Ligand **7i** showed the best docking score (−9.92 kcal*mol^−1^), followed by ligand **7** **g** (−10.68 kcal*mol^−1^), with favorable H‐bonding residues GlnA:64, AsnA:103, GlnA:112 and SerA:111 within the colorectal active site (PDB code: 2HQ6). DFT analyses elucidated electronic properties, including geometry optimization, frontier molecular orbitals, and E_HOMO_–E_LUMO_ (0.12766–0.15523 eV), correlating with observed bioactivity. The theoretical predictions by DFT and in silico docking and ADMET‐physicochemical properties were well supported by the experimental studies and revealed strong anticancer activity. Collectively, these findings highlight the benzo[*b*]thiophene scaffold as a highly promising candidate for the development of new anticancer inhibitors, warranting further investigation.

## Graphical abstract

Innovative synthesis of a novel benzothiophene–based bis–oxadiazole derivatives. The structural characterization was performed using experimental and theoretical methods. Significant anticancer activity against selected cancer cell lines. Three derivatives showed good activity **7b, 7g** and **7i** with IC_50_ values 7.5 ± 1.04 and 9.5 ± 1.01 µg/mL. DFT calculations and molecular docking provided insight into reactivity and biological interactions. ADME–Tox predictions of compounds **7a–7k** have favorable pharmacokinetic and safety profiles of attractive therapeutic research.

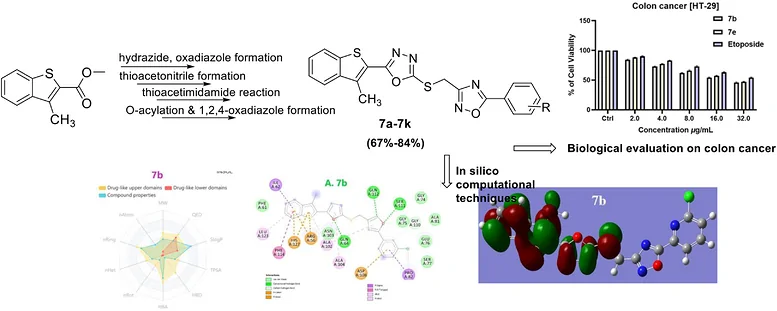

## 1 Introduction

Cancer continues to pose a global health challenge. The rising incidence of cancer worldwide is caused by a variety of reasons, including ageing populations, bad lifestyle choices, infections, and environmental exposures [1]. Cancer has detrimental psychological effects and costs the public and health systems money [2]. There were 18.1 million new cases of cancer and 9.6 million deaths from the disease in 2018. Globally, there were 9.7 million cancer‐related deaths and 20 million new cases in 2022, with emerging nations accounting for roughly 70% of these deaths [3]. WHO estimates that an additional 30 million cases of cancer will be reported by 2040 [4]. According to estimates from the International Agency for Research on Cancer (IARC), 1 in 5 individuals worldwide will have cancer at some point in their lives, and 1 in 8 men and 1 in 11 women will pass away from the disease. This means that over 50 million people are still alive five years after receiving a cancer diagnosis. The lack of tumor selectivity and pharmacokinetic problems, such as low water solubility, which impair the drug's circulation time and bioavailability and restrict its clinical use, are the main reasons why many approved medications still exhibit high systemic toxicity [5]. The aforementioned drawbacks of traditional anticancer medications are the cause of the ongoing demand for the creation of new anticancer medications or enhancements to existing ones.

Worldwide, breast cancer is the most prevalent cancer to be diagnosed in women [6]. In their lifetime, about one in eight women may receive a diagnosis of aggressive breast cancer [7]. Data from 2024 show that 42,250 women in the US lost their lives to breast cancer, while 310,720 women received a diagnosis of invasive breast cancer. About 80% of cases of breast cancer are estrogen‐dependent, which means that when oestrogen is present, there is a probability that malignant cells will grow [8]. As a result, the treatment of cancer requires chemicals that either block oestrogen receptors or inhibit the generation of oestrogen [9]. Due to a number of similarities between colon cancer and rectal cancer, colorectal cancer (CRC), a malignant tumor that develops in the colon's tissue, is categorized as a combination of the two diseases. Colorectal cancer accounted for 12.7% of new cancer diagnoses and 12.4% of all cancer‐related deaths across the 27 European countries in 2020 [10]. The estimated distribution in the EU of new cases of colorectal cancer is strictly dependent on the patients’ age group, and the survival rates for cancers diagnosed in 2018 has shown severe country‐dependent heterogeneity [11].

Heterocycles are the most important traditional domain of organic chemistry, and they are receiving increased study attention due to its industrial, antibacterial, and medicinal applications. Many natural substances, such as DNA, RNA, haemoglobin, chlorophyll, vitamins, and others are largely composed of heterocyclic rings [12]. A privileged family of chemical compounds is represented by functional derivatives of the 1,2,4‐oxadiazole ring from the standpoint of medicinal chemistry and target‐based drug design. The five‐membered 1,2,4‐oxadiazole heterocyclic ring has attracted a lot of attention because of its unique bioisosteric properties and wide variety of biological activities. It has been discovered and used as a metabolically stable analogue of an ester or amide functionality in pharmacologically significant molecules [13]. In fact, molecules containing the 1,2,4‐oxadiazole moiety possess a number of valuable biological activities, including antiproliferative [13, 14], anti‐inflammatory [15], antioxidant [16], antibacterial [17], antimycobacterial [18], antiparasitic [19], antidiabetic [20], antifungal [14], antiviral [21], anti‐infective, [22] in general [23], and other activities. The 1,2,4‐oxadiazole fragment is also known to be a building block for the synthesis of novel high‐energy materials [24].

Sulfur‐containing heterocycles are a significant class of molecules with several applications in chemical and medicinal science [25]. Benzo[*b*]thiophene and its derivatives are of particular interest among the sulfur‐containing heterocycles since they are candidates that can be used to create new, potent lead molecules for drug creation because they have structural characteristics with active substances [26]. Various stages of drug development have seen the presence of compounds with the benzothiophene moiety as anticancer [27], antimicrobial [28], antioxidant, anti‐inflammatory [29], and endothelial cell activation inhibitors [30], urokinase inhibitors [31], antidepressants [32], and cognition‐enhancing agents with potential use in Alzheimer's disease [33]. These basic structures are found in many pharmaceutical compounds, including sertaconazole, zileuton, and raloxifene, as well as in naturally occurring chemical molecules [34]. From the standpoint of target‐based drug design and medicinal chemistry, functional derivatives of the 1,3,4‐oxadiazole ring are a special class of organic molecules. Indeed, compounds with the 1,3,4‐oxadiazole moiety have several beneficial biological properties, such as antibacterial, antifungal, antiparasitic, antihypertensive, antimycobacterial, anti‐inflammatory, antiproliferative, antiviral, antidiabetic, and other activities [35, 36, 37, 38, 39]. Nesapidil, Zibotentan, Tiodazosin, Fenadiazole, Raltegravir, Pleconaril, Butalamine, and Arzoxifene are only a few of the many commercially licensed medications that include 1,3,4‐oxadiazole, 1,2,4‐oxadiazole, and a benzo[*b*]thiophene ring (Figure 1) [40]. With these oxadiazole‐based drug molecules approved by the US FDA and more in clinical trials, oxadiazole has a promising future in the drug discovery process for chemotherapy [41].

**FIGURE 1:**
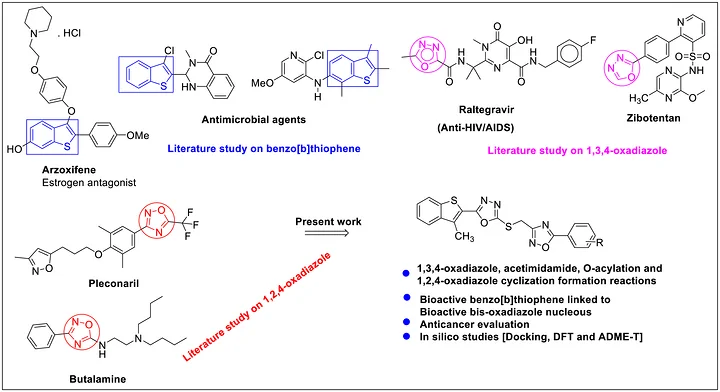
Literature review on benzo[*b*]thiophene, 1,3,4‐oxadiazole & 1,2,4‐oxadiazole and current study findings with published literature.

Computer‐aided drug discovery is pivotal in modern medicinal chemistry, as the designed molecules can be pre‐screened to understand their activity. This process can reduce the cost of drug discovery and aid in further testing of the best molecule. Protein‐molecule interactions can be studied by molecular modelling, and molecular docking computations are performed to examine these interactions [42]. Molecular docking helped us explain the biological activity of our recently synthesized ligands, in addition to our recent research where we used other in silico approaches, such as quantum chemistry and molecular mechanic calculations. Based on this rationale, the present study employed a molecular hybridization strategy in which the benzo[*b*]thiophene nucleus was linked to a bis‐oxadiazole ring and diversified through aryl substitution. The use of density functional theory (DFT) and molecular docking is therefore not incidental; rather, these approaches directly support the study objective by correlating structural and electronic properties with binding trends and experimental antifungal performance. In the quest for the most effective drug candidates and based on reported literature, this is the first report to the best of our knowledge describing the designed benzo[*b*]thiophene bearing‐bis‐oxadiazole analogues **7**(**a–k**) via three step reactions, which were demonstrated as potential inhibitors targeting the anticolon cancer *E. coli* (PDB code: 2HQ6), with compound **7i** emerging as the most promising member of the series in both in silico and biological analyses. To forecast their pharmacokinetic behavior and toxicity profiles, an in silico absorption, distribution, metabolism, excretion, and toxicity (ADMET) analysis was also carried out. Important characteristics, including as solubility, lipophilicity, intestinal absorption, metabolic stability, clearance, and toxicity risks, were assessed to determine their safety and drug‐likeness. The results offer valuable insights into their mechanisms of action, pharmacokinetic behavior, and potential limitations, thereby laying a solid foundation for their further development as effective and sustainable therapeutic candidates.

## 2 Results and Discussion

### 2.1 Chemistry

Therefore, as part of our ongoing research effort on the synthesis of novel bioactive heterocycles [28, 43, 44], we are very much engrossed in the design and synthesis of the title compounds. Targeted moieties were created by us using a three‐step reaction that was used in mild reaction conditions (Scheme 1). In the right circumstances, cyclisation of the 1‐(2‐chlorophenyl)ethan‐1‐one group (**1**) with methyl 2‐mercaptoacetate by stirring it with NaH in DMF for 3 h while under reflux. Then followed the key intermediate **2**, which was afforded via the mixture of ester compound **1** in ethanol, hydrazine hydrate was added for 4 h. As a further step, we employed the cyclization reaction of oxadiazole in which the hydrazide **2** was reacted with CS_2_ in the presence of KOH in ethanol to give 5‐(3‐methylbenzo[*b*]thiophen‐2‐yl)‐1,3,4‐oxadiazole‐2‐thiol (**4**) in good yields (70%). Monitoring of the reaction was done by thin‐layer chromatography, and completion of the reaction was confirmed by a single spot in TLC under a UV lamp.

**SCHEME 1:**
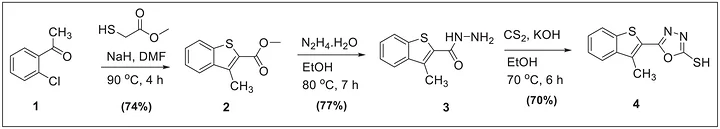
Synthesis of benzo[*b*]thiophene intermediate with 1,3,4‐oxadiazole **5**.

Second, we have synthesized substituted 1,2,4‐oxadiazole compounds (**7a‐7k**) starting from key intermediate acetimidamide and corresponding acid derivatives in three steps (Scheme 2). Accordingly, compound **4** reacted with 2‐bromoacetonitrile in the presence of potassium carbonate as a base catalyst under refluxing conditions using acetone as a solvent, which resulted in a corresponding thio‐acetonitrile **5** (70%). The acetimidamide compound (**6**) was prepared by condensation reaction, which was the reaction of 2‐((5‐(3‐methylbenzo[*b*]thiophen‐2‐yl)‐1,3,4‐oxadiazol‐2‐yl)thio)acetonitrile (**5**) and hydroxylamine hydrochloride, under the influence of a catalytic amount of TEA in DCM in a good to excellent yield (73%). In addition to this, the chemical reactivity of compound **5** with several acids was also investigated to synthesize a series of novel oxadiazole derivatives via O‐acylation and cyclization. Therefore, when acetimidamide **5** was treated with corresponding acids in DCM as a solvent in the presence of coupling reagent EDC. HCl as a catalyst, it afforded acylated product. Finally, the 1,3,4‐oxadiazole **7** was formed through the acylated intermediate in ethanol and in the presence of sodium acetate, an intramolecular cyclization and finally elimination of a water molecule [45]. All the compounds **7a‐7k** were obtained in moderate to good yields, and the structures of all compounds **7a‐7k** were confirmed by their spectroscopic data.

**SCHEME 2:**
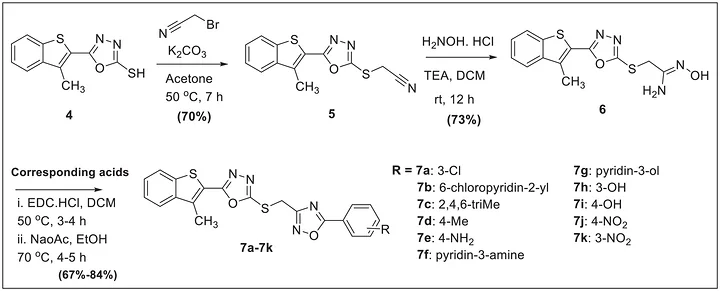
General synthetic procedure of the 1,3,4‐oxadiazole linked to 1,2,4‐oxadiazole conjugates (**7a‐7k**).

### 2.2 Spectral Data

All the structures of the series of products **7a–** **7** **k** were also confirmed through the analytical followed by spectral data analysis (see Experimental). The ^1^H‐NMR spectra of **7a** showed chemical shifts of protons located on benzo[*b*]thiophene methyl group (H_1_) as singlet at *δ* 2.27 ppm, whereas the oxadiazole substituted methylene proton (H_2_) exhibited as singlet at 4.57 ppm, respectively. The singlet signal for the phenylic proton (H_7_) was observed at *δ* 8.02 ppm, while the remining signals of the aromatic group appear at *δ* 7.42‐7.83 ppm, confirming the formation of final product. In the ^13^C‐NMR spectra, the signals for the 1,2,4‐oxadiazole, and 1,3,4‐oxadiazole carbons were present *δ* at 176.2, 162.4, and 159.2 ppm, while the aromatic carbon signals for **7a** appear at *δ* ranging from 143.5−119.6 ppm. Also, when we examined the spectrum of compound **7a**, while the signal of the −SCH_2_ group appears at 35.5 ppm, the −CH_3_ signals of substituted benzo[*b*]thiophene appears at 15.8 ppm. Molecular weight at HRMS (*m/z*) = 441.0310 [M+1]^+^ of mass spectrum is evidence of 5‐(3‐chlorophenyl)‐3‐(((5‐(3‐methylbenzo[*b*]thiophen‐2‐yl)‐1,3,4‐oxadiazol‐2‐yl)thio)methyl)‐1,2,4‐oxadiazole **(7a)**, which were in accordance with the proposed structures representing their correct molecular ion peaks beside some other important peaks.

Derivatization was confirmed by the disappearance of peaks of amine (NH_2_) and OH at 3348 and 3425 cm^−1^ of acetimidamide and the appearance of C═N and C═C at 1448 and 1576 cm^−1^ in compound **7b**. Moreover, the peaks of COC and CSC linkers at 1158 and 1255 cm^−1^ in compound **7b** also confirmed the formation of oxadiazole. IR spectra peaks of sp^2^ (═CH) and sp^3^ (CH) functional groups were found at 3070 and 2850 cm^−1^ in the **7b** derivative. Compound **7b** showed a typical singlet signal that appeared at *δ* 2.28 (H_1_), and 4.52 (H_2_) ppm due to the methyl group, and thio‐methylene, respectively, in the ^1^H‐NMR spectrum. In addition to this, a multiplet peaks is observed in the region: 7.31–8.48 (H_3_‐H_9_) ppm assignable to the seven aromatic hydrogens. On the other hand, the ^13^C‐NMR spectrum of **7b** showed three signals at *δ*  = 164.84, 162.41, and 159.17 ppm characteristic for oxadiazole groups, respectively, in addition to thirteen aromatic carbon signals in the range of *δ* 120.52–157.29 ppm. In the ^13^C‐NMR signal, *S*‐methylene and benzo[*b*]thiophene carbons exhibited at *δ* 35.71 and 14.86 ppm for compound **7b**. Besides, its HRMS spectrum revealed a molecular ion peak at *m/z* = 443.9798 [M+1]^+^ for the compound 5‐(6‐chloropyridin‐2‐yl)‐3‐(((5‐(3‐methylbenzo[*b*]thiophen‐2‐yl)‐1,3,4‐oxadiazol‐2‐yl)thio)methyl)‐1,2,4‐oxadiazole (**7b**).

### 2.3 Antiproliferative Evaluation

The novel bis‐oxadiazole derivatives of benzo[*b*]thiophene were evaluated for cytotoxicity using human colorectal adenocarcinoma (HT‐29; RRID: CVCL_0320), human colorectal carcinoma (HCT‐116; RRID: CVCL_0291), human colorectal adenocarcinoma (Caco‐2; RRID: CVCL_0025), and human breast carcinoma (MDA‐MB‐231; RRID: CVCL_0062) cell lines. To determine the cytotoxic properties of the novel benzo[*b*]thiophene compounds, the four cell lines (HT‐29, HCT‐116, Caco‐2, and MDA‐MB‐231) were subjected to an MTT (3‐(4,5‐dimethyl thiazol2‐yl)‐2,5‐diphenyltetrazolium bromide)assay [46, 47] after incubation with the compounds at concentrations ranging from 0.5 to 100 µg/mL for a duration of 24 h. A 24 h time‐dependent cell viability assay was carried out through which the IC_50_ values (µg/mL) of the synthesized **7a–7k** were obtained according to the MTT assay results as presented in Table 1. The concentration (µM) of test compounds necessary to kill 50% of the cell population (IC_50_) was established by generating data to create a dose–response curve. The mean IC_50_ population from three different trials served as the basis for the cytotoxic effects. The most potent anticancer effects were observed with compounds **7b**, **7e**, **7g,** and **7i** exhibiting excellent activity on the colorectal and breast cancer. Specifically, compounds **7b** and **7e** showed promising antiproliferative effects against HT‐29 cell, with IC_50_ values of 6.2 ± 0.67 and 6.8 ± 0.84 µg/mL, respectively, when compared to etoposide (IC_50_ = 7.5 ± 1.04 µg/mL) (Figure 2). Likewise, compound **7g** and **7i** displayed strongest anticancer activity against the same cell, with IC_50_ values of 7.4 ± 1.90, and 12.5 ± 1.70 µg/mL, respectively. Compounds **7d** and **7j** showed less activity than other derivatives, with IC_50_ values against the HT‐29 cell line of 42.2 ± 1.03 and 44.1 ± 0.47 µg/mL, respectively. Also, the prepared analogues **7b**, **7e**, **7g,** and **7i** showed potent anticancer activity which showed IC_50_ values of 5.1 ± 0.78, 7.9 ± 0.74, 8.6 ± 1.33, and 10.0 ± 2.10 µg/mL against human colorectal carcinoma (HCT‐116) cell line, respectively. Bis‐oxadiazole **7d** containing *para*‐methyl phenyl ring, on the other hand, shows low anticancer activity (IC_50_  =  36.3 ± 0.55 µM). According to the data presented in Table 2, **7i** showed the highest antitumor activity against human colorectal adenocarcinoma (Caco‐2), and (human breast carcinoma (MDA‐MB‐231) cell lines among the synthesized compounds with IC_50_ values of 8.8 ± 1.01 and 9.5 ± 0.38 µg/mL, respectively.

**TABLE 1:** Anticancer efficacy and IC_50_ values of the title compounds in µg/mL.

Codes	IC_50_ ± SD^a^ (µg/mL)			
	HT‐29	HCT‐116	Caco‐2	MDA‐MB‐231
**7a**	15.5 ± 1.06	14.3 ± 1.02	20.6 ± 1.08	>50
**7b**	6.2 ± 0.67	5.1 ± 0.78	7.9 ± 1.15	11.2 ± 1.02
**7c**	>50	27.6 ± 0.88	>50	32.7 ± 1.15
**7d**	42.2 ± 1.03	36.3 ± 0.55	29.1 ± 0.60	37.5 ± 1.19
**7e**	6.8 ± 0.84	7.9 ± 0.74	13.0 ± 1.20	>50
**7f**	>50	28.7 ± 0.90	21.9 ± 0.60	32.5 ± 0.95
**7g**	7.4 ± 1.90	8.6 ± 1.33	8.1 ± 0.89	10.3 ± 0.54
**7h**	14.2 ± 1.02	>50	17.10 ± 1.70	25.9 ± 1.04
**7i**	12.5 ± 1.70	10.0 ± 2.10	8.8 ± 1.01	9.5 ± 0.38
**7j**	44.1 ± 0.47	>50	>50	>50
**7k**	30.20 ± 0.88	28.6 ± 2.02	>50	44.2 ± 1.33
**Etoposide**	7.5 ± 1.04	6.0 ± 1.21	9.5 ± 1.01	8.4 ± 0.91

^a^Values are expressed as mean ± SD of three independent triplets (*n* = 3); >50: not active.

**FIGURE 2:**
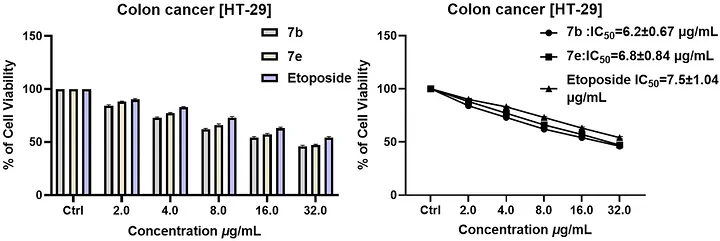
Graphical representation of % cell viability of synthesized compounds (**7b, 7e,** and **Etoposide**) (Left); IC_50_ values of synthesized compounds against colon cancer cell line (**7b, 7e,** and **Etoposide**) (Right). Data are expressed as means ± standard deviation (SD) (*n* = 2). Statistical analysis was performed using GraphPad Prism 10.0. The significance level has been set to *p* < 0.05 [48].

**TABLE 2:** Interacting residues and docking constant of novel *bis*‐oxadiazoles with colon cancer target (2HQ6).

Ligands	Docking constant	Dissociation constant	H‐bond amino acid residues	Hydrophobic amino acid residues
**7b**	−9.70 kcal*mol^−1^	77.96 nM	GlnA:64, GlnA:112 SerA:111	**Van der Waals**: PheA:61, AsnA:103, GlyA:74, GlyA:75, AlaA:81, GluA:76, SerA:77 **Pi‐alkyl & alkyl**: LeuA:123, AlaA:102×3, AlaA:104, ProA:82; **Pi‐sigma**: IleA:62, ProA:82; **Pi‐Pi stacked**: PheA:114; **CH‐bond**: GlyA:110; **Pi‐anion**: HisA:127×2, ArgA:56×2; **Pi‐cation**: AspA:108
**7g**	−9.92 kcal*mol^−1^	53.76 nM	GlnA:64 AsnA:103 GlnA:112 SerA:111	**Van der Waals**: PheA:61, AlaA:104, GlyA:72, AlaA:81, SerA:77, GlyA:75, GlyA:74 **Pi‐alkyl & alkyl**: LeuA:123, IleA:62, AlaA:102 **Pi‐sigma**: AlaA:102, ProA:82; **Pi‐Pi stacked**: PheA:114 **Pi‐cation**: HisA:127×2, ArgA:56×2 **Pi‐donor H‐bond**: GlyA:110
**7i**	−10.12 kcal*mol^−1^	37.91 nM	GlnA:64 AsnA:103 GlnA:112 SerA:111 GluA:76	**Van der Waals**: PheA:61, IleA:62, PheA:114, GlyA:72, SerA:77, GlyA:74, GlyA:75, AlaA:104 **Pi‐alkyl & alkyl**: LeuA:123, AlaA:102, ProA:82 **Pi‐sigma**: AlaA:102; **Pi‐Cation**: HisA:127×2, ArgA:56×2; **Pi‐donor H‐bond**: GlyA:110

The benzo[*b*]thiophenes **7g** and **7i** (IC_50_  = 8.1 ± 0.89 and 8.8 ± 1.01 µg/mL, respectively) have encouraging anticancer activity against Caco‐2, while 1,3,4‐thiadiazoles **7e**, and **7h** have moderate cytotoxic activity (IC_50_  =  13.0 ± 1.20 & 17.10 ± 1.70 µg/mL) (Figure 3). As a reference standard, etoposide showed an IC_50_ value of 9.5 ± 1.01 µg/mL against Caco‐2. The results obtained revealed that compounds **7a**, and **7b** exhibited significant anticancer potency against the tested Caco‐2 cell line at IC_50_ ranging from 7.9 ± 1.15 to 20.6 ± 1.08 µg/mL. The hydroxy protected derivatives **7g** and **7i** were more active (IC_50_ = 10.3 ± 0.54 and 9.5 ± 0.38 µg/mL, respectively) than their *meta*‐hydroxy protected precursor **7h** (IC_50_ = 25.9 ± 1.04 µg/mL), when active breast cancer cell line MDA‐MB‐231. The results showed also, that compound **7b** exhibited a considerable wide range of anticancer potency against the MDA‐MB‐231 of the tested breast cancer with low IC_50_ values of 11.2 ± 1.02 µg mL^−1^. Otherwise, compounds **7c, 7d,** and **7f** revealed moderate anticancer effect at the inhibitory concentrations ranging from 32.5 ± 0.95 to 37.5 ± 1.19 µg/mL against the tested MDA‐MB‐231. Meanwhile, oxadiazols **7a**, **7e,** and **7j** did not showed antibreast cancer effect at even high concentrations (i.e., >50 µg/mL) against the MDA‐MB‐231 cell line.

**FIGURE 3:**
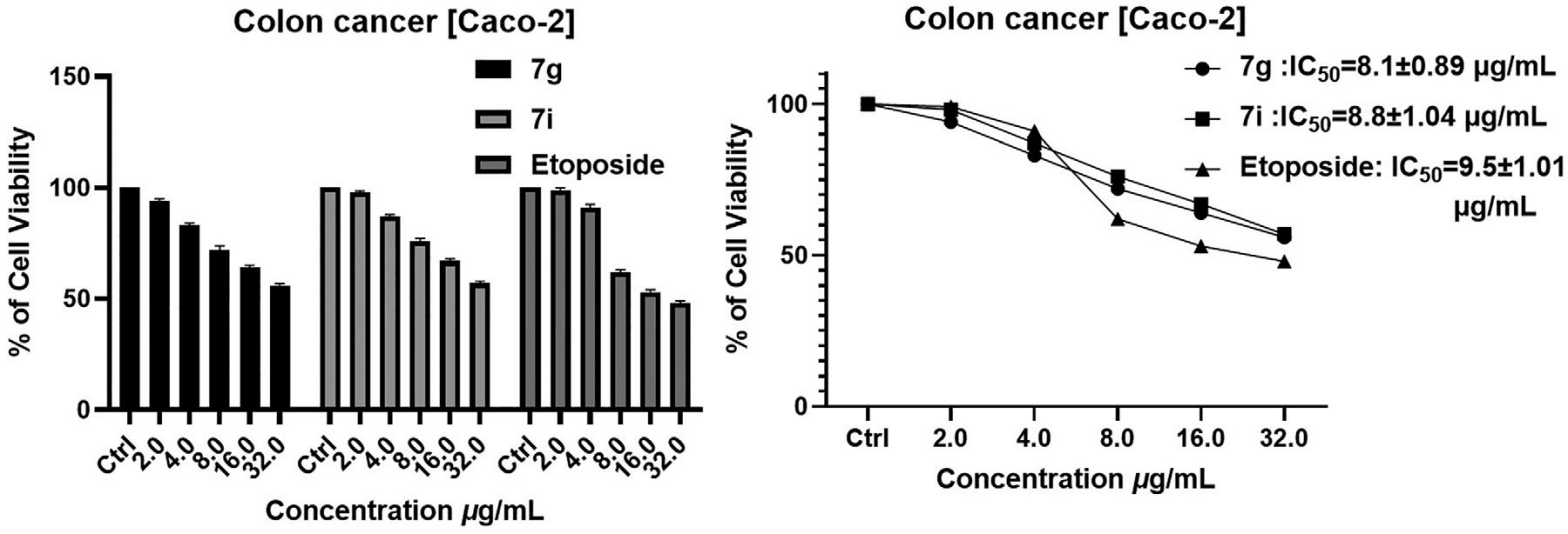
Visual (% cell viability and IC_50_ values) anticancer activity of final bis‐oxadiazole analogues (**7g, 7i,** and Etoposide).

### 2.4 Structure Activity Relationship (SAR) Studies

The terminal amine is a crucial pharmacophore, according to these studies, most likely mediating ionic or H─bond interactions with the molecular target and/or improving cellular absorption. Compound **7b**’s electron‐withdrawing, potently activating chloropyridin‐2‐yl group might be the cause of improved breast and colon cancer treatments. The anticolon and antibreast cancer properties of compounds **7c**, **7d**, and **7e** are diminished due to the presence of weakly deactivating electron‐donating methyl and amine groups. The IC_50_ values of the produced analogues (**7a‐7k**) were either three times lower or equivalent to the corresponding IC_50_ values. The synthetic analogues with hydroxy substitution on the fourth and third positions of the phenyl/pyridine ring are the most active of all the derivatives, according to the anticancer activity investigation. Because of its large chloro‐pyridinyl linker, which enables the oxadiazole nucleus to be orientated optimally for CH‐bonding and H‐bonding interactions within the colon binding pocket, compound **7b** demonstrated increased cytotoxic activity. Compounds containing methylphenyl linkers, like **7c** and **7d**, on the other hand, exhibited decreased activity, indicating that too much flexibility reduces binding affinity. The potency of all four cell lines was significantly reduced when the protective group from hydroxy was removed to create derivatives with free nitro groups (**7j** and **7k**).

### 2.5 Docking Analysis

To assess the therapeutic potential of the synthesized compounds, molecular docking studies were conducted against one major virulence factor of human colon cancer (PDB code: 2HQ6) [49, 50]. These targets are critical for cancer colonization and tissue damage. The binding affinities and key interacting residues for the most active compounds **7b, 7g,** and **7i**, are summarized in Table 2. The binding scores correlated with the IC_50_ values of the compounds, with energy values ranging from −9.70 to −10.12 kcal*mol^−1^. The –2D, –3D, and ligplot structures of the docked complex (**7b**) was shown in Figure 4. Compound **7b** bound to the target via H‐bonds with GlnA:64 [O15…NE2 (3.09 Å)], GlnA:112 [(N19…N, 3.01 Å)], and SerA:111 [(O20…N, 2.98 Å)] with its aliphatic tail protruding into a hydrophobic Pi‐alkyl & alkyl groove lined by LeuA:123, AlaA:102, AlaA:104, and ProA:82. In contrast, benzo[*b*]thiophene penetrated deeper into the binding crevice, secured by four Pi‐anion bonds with HisA:127 and ArgA:56. Crucially, this ligand formed a strong Pi–sigma interactions with IleA:62, ProA:82, and Pi–cation bond with AspA:108, stabilizing force absent in the docking pose of compound **7b**. Compound‐**7b** consistently demonstrated superior binding affinities driven by its capacity to form specific polar interactions (LeuA:123, AlaA:102, AlaA:104, ProA:82, PheA:61, AsnA:103, GlyA:74, GlyA:75, AlaA:81, GluA:76, and SerA:77), while maintaining hydrophobic integrity via its benzo[*b*]thiophene, oxadiazole chloropyridine moieties.

**FIGURE 4:**
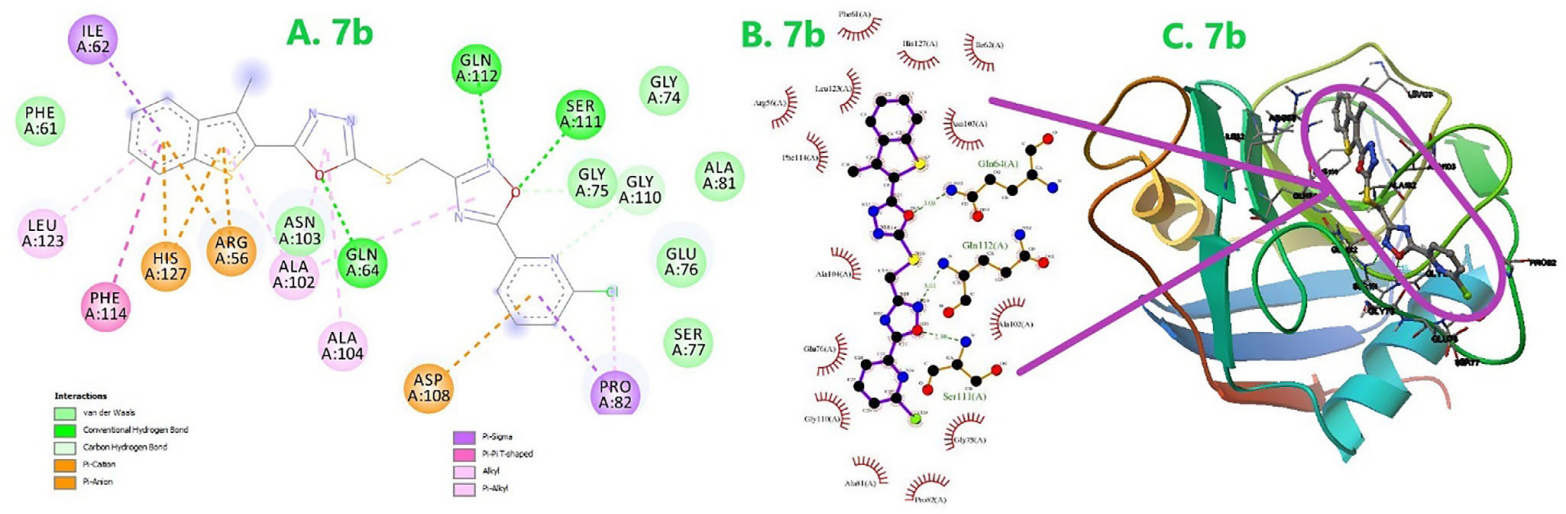
Docking representation of the –2D (A), Ligplot (B), and –3D complex (C) colon cancer therapy [2HQ6].

Ligand **7g**, for instance, inserts its oxadiazole framework into this cleft and hydroxy pyridine via four H‐bonds to residues GlnA:64 (N12…NE2, 3.13 Å), AsnA:103 (O15…N, 2.85 Å), GlnA:112 (3.08 Å), and SerA:111 (O20…N, 3.00 Å). Its multiple aromatic rings also engage in hydrophobic Pi–Pi and Pi–cation stacking interactions (notably with PheA:114, HisA:127×2, ArgA:56×2) that reinforce binding. Likewise, this ligand was found to snugly occupy the same pocket, bridging benzo[*b*]thiophene domain interface via Pi‐alkyl bonds (with LeuA:123, IleA:62, AlaA:102). The 1,3,4‐oxadiazole and pyridinyl rings docked to the target through one Pi‐sigma interaction with the residues AlaA:102, and ProA:82 at 3.88 and 4.10 Å, respectively. In addition, compound **7g** with binding energy −9.92 kcal mol^−1^ showed one Pi–donor H‐bond with the target residue GlyA:110 at 2.74 Å (Figure 5). Finally, this ligand showed seven van der Waals week forces with residues PheA:61, AlaA:104, GlyA:72, AlaA:81, SerA:77, GlyA:75, and GlyA:74 in the active site of colon cancer. Compound **7i** exhibited one Pi‐sigma and one Pi‐donor H‐bond interactions with the residues AlaA:102 and GlyA:110 at distance 3.36 and 3.91 Å, respectively. Moreover, benzo[*b*]thiophene core declared Pi‐cation interaction with the residues HisA:127×2, ArgA:56 at 3.28 and 3.78 Å accordingly. Finally, this ligand showed five HB interactions with GlnA:64, AsnA:103, GlnA:112, SerA:111, and GluA:76 at 2.81, 2.90, 3.04, 2.89, and 2.87 Å, respectively, effectively stabilizing the molecule in the binding pocket. Whereas, (anticancer drug‐**7i**) exhibited eight van der Waals forces with the active site residues of PheA:61, IleA:62, PheA:114, GlyA:72, SerA:77, GlyA:74, GlyA:75, and AlaA:104 in the active site of colon receptor. Thymol exhibited the strongest binding affinity (−10.12 kcal*mol^−1^) with a high *K*i value (37.91 nM), interacting via hydrophobic Pi‐alkyl bonds with LeuA:123, AlaA:102, ProA:82 (Figure 5).

**FIGURE 5:**
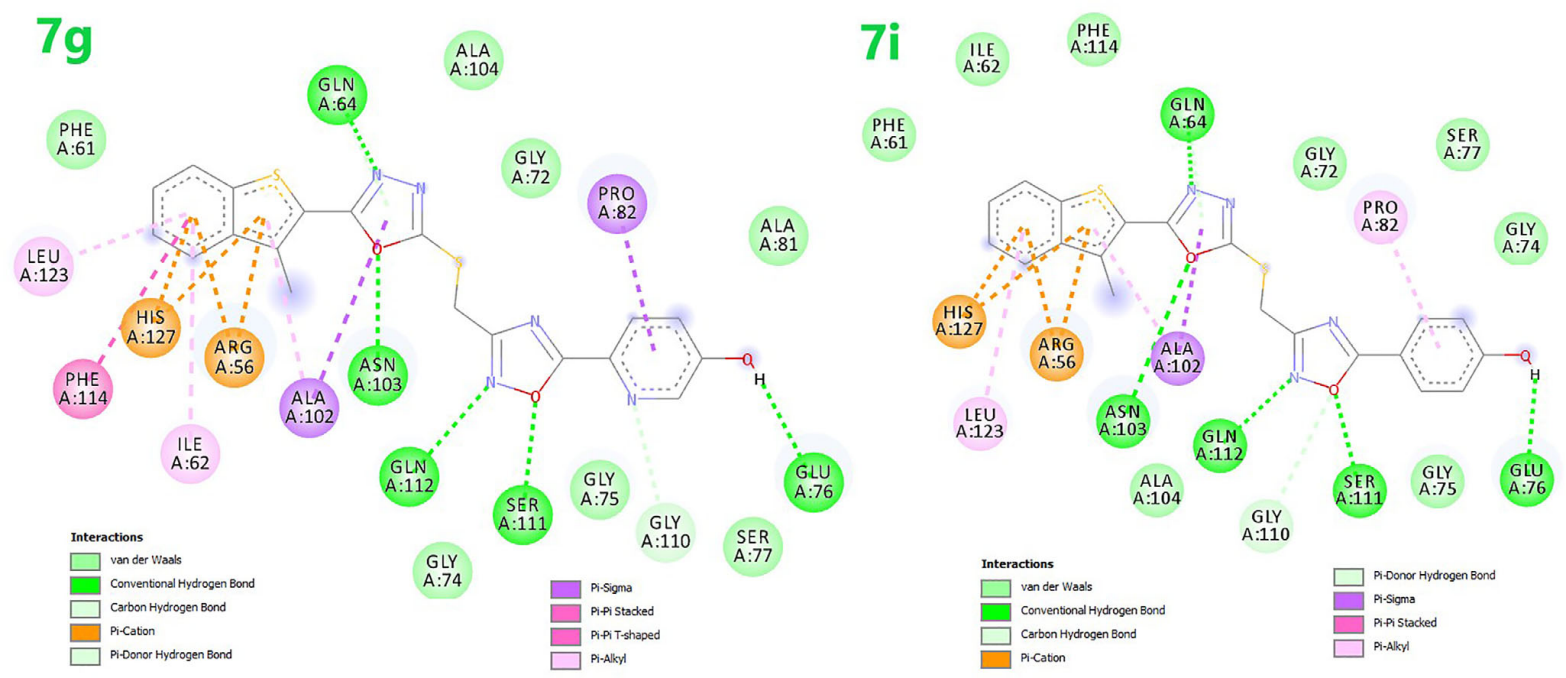
2D–molecular interactions of ligands **7g** and **7i** complexed with 2HQ6 protein.

The enhanced anticancer activity of the best docked compounds **7b**, **7g,** and **7i** was ascribed to the presence of electron withdrawing and donating groups –Cl, and –OH attached to aromatic and heteroaromatic rings such as pyridine. Among the derivatives, compound **7i** exhibited the strongest binding affinity (−10.12 kcal*mol^−1^) and the lowest inhibition constant (*K*i = 37.91 nM), forming five H‐bonds, compound **7g** showed similar high binding affinity (−9.92 kcal*mol^−1^) with low K_i_ values (53.76 nM), although five H‐bond interactions were observed. Other derivative, including compound **7b** and **7d**, demonstrated moderate binding affinities (−9.70 to −8.10 kcal*mol^−1^) and formed three hydrogen bonds with specific K_i_ values (77.96 nM to 67.28 nM).

### 2.6 Computational Analysis of Molecular Properties

To gain deeper insight into the electronic characteristics and potential reactivity of compounds **7b** and **7i**, density functional theory (DFT) calculations were performed [51]. Using the Gaussian 09 software package with the B3LYP/6‐31G(d) assay [52]. The energy separation between them, termed the E_HOMO_–E_LUMO_ gap, provides crucial information about the compound's reactivity profile and overall stability (Figure 6). The Pi–donor delocalized electron density is condensed on benzo[*b*]thiophene and 1,3,4–oxadiazole of the compound **7b** (as shown in the HOMO orbital). Meanwhile, the Pi–acceptor electron cloud is localized on the substituted 1,2,4–oxadiazole skeleton, as shown in the LUMO orbital for compound **7b**. The existence of an electron‐donating group such as OH (fourth position on the phenyl ring) in compound **7i** has not shifted the electron cloud efficiency from the benzo[*b*]thiophene skeleton to the opposite sides. Thus, the donor–Pi–acceptor system (D–Pi–A) of benzo[*b*]thiophene/1,3,4–oxadaizole of compound **7i** played a pivotal role in the Charge Transfer Interaction (CTI) with the active site of the target enzyme. Compound **7b** shows the smallest E_HOMO–LUMO_ gap (Δ*E* = 0.12766 eV). Highest chemical reactivity and better charge‐transfer ability. Compound **7i** has the largest energy gap (Δ*E* = 0.15523 eV) compared to other derivatives. Greater kinetic stability and lower reactivity. The spatial distribution of these orbitals provides additional insights into the compound's behavior. All the potent bis‐oxadiazole compounds show moderate HOMO–LUMO gaps, suggesting a balance between stability and biological/electronic activity, suitable for medicinal chemistry relevance.

**FIGURE 6:**
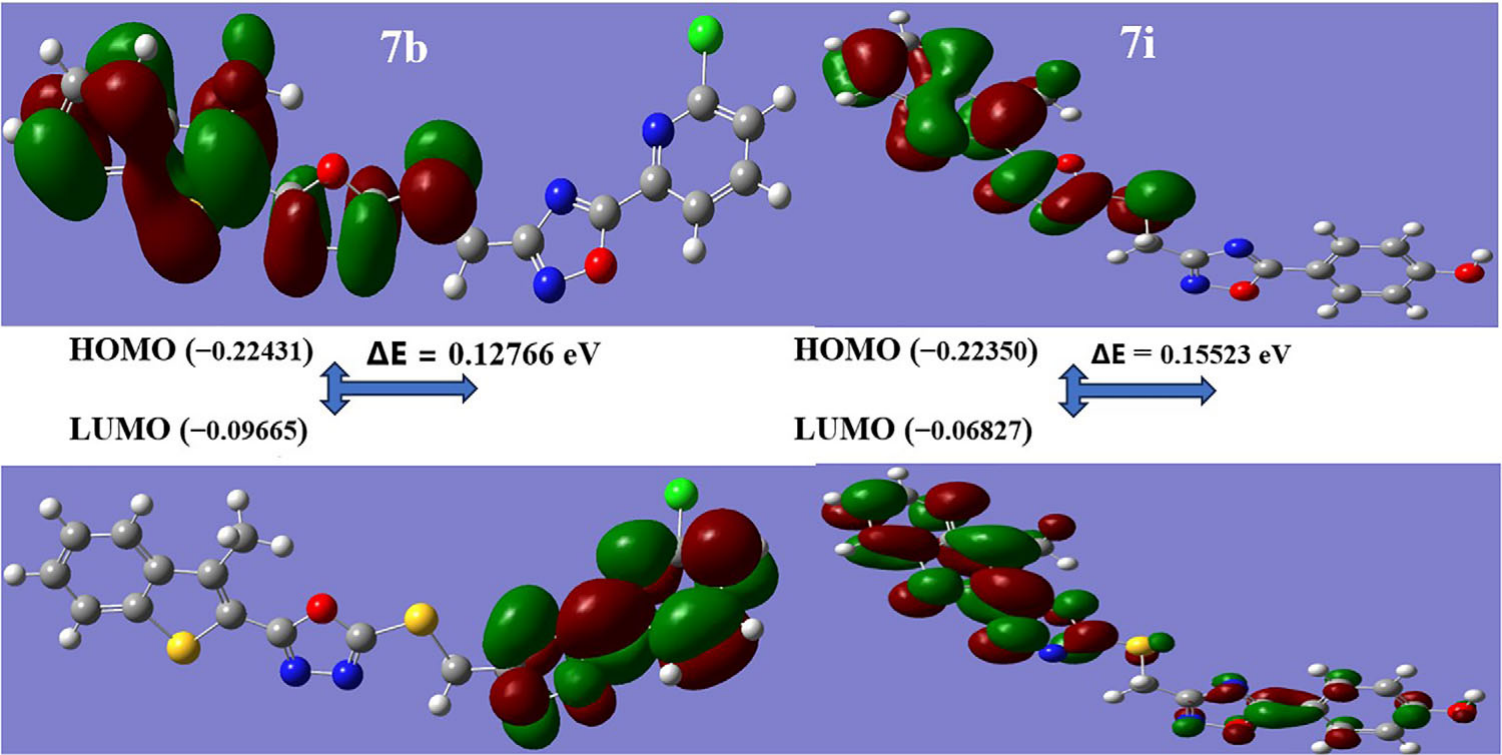
DFT calculated HOMO and LUMO top to bottom for **7b**, and **7i** at the B3LYP/6–31G(d) level of theory.

### 2.7 Evaluation of Drug‐Likeness and ADME Properties

In recent years, computational methods for analyzing and forecasting ADME aspects have gained popularity. These methods use in silico simulations of membrane permeability, biomolecular interactions during absorption and excretion, and structural stability throughout the metabolic process. For this experiment, the target compounds were initially sketched in ChemBioDraw Ultra 14.0, and their SMILES notations were extracted for further computer analysis [53]. These SMILES strings were then processed using three key prediction systems: SwissADME, ADMET lab3.0, and AdmetSAR 3.0 [54, 55]. Studies on drug similarity assessment and ADME‐Tox prediction of the generated compounds **7a‐7k** were carried out using reliable online servers (Tables 3 and 4). High solubility is shown by the tested chemicals' hydrogen bond donors (HBD) and hydrogen bond acceptors (HBA), which fall within the permissible ranges of 10 and 5, respectively. They also have strong absorption qualities (%HIA). They have ideal lipophilicities (as determined by MLogP). Additionally, analogues with a rotatable bond of <10 and a TPSA of ≤ 40 Å^2^ are thought to have high bioavailability, according to Veber et al. [56]. More rotatable bonds allow a ligand to be more flexible and can be rearranged to better interact with the active site cavity. They also showed significant absorption in the gastrointestinal tract. Blood–brain barrier (BBB) permeability was absent in all of them, suggesting that the central nervous system (CNS) was unaffected. All things considered, the compounds' pharmacokinetic characteristics complied with Lipinski's rule of five, indicating that they would be useful as anticancer medication candidates [57]. The polarity, flexibility, and size of the molecule are all reflected in these properties, which together control solubility, membrane permeability, and absorption. The only characteristic of compounds **7g** and **7i** that differs substantially from its ideal value is the degree of polarity and insaturation (Figure 7). Compounds that satisfied these requirements were thought to be promising for additional biological analysis.

**TABLE 3:** Physicochemical and pharmacokinetics properties of final *bis*‐oxadiazole compounds (**7a‐7k**).

Entry	TPSA	LogP	LogS	GI absor.	Lipinski	Pfizer	GSK	Golden Tri.	Muegge	Veber	Synth. score	Pains
**7a**	77.84	5.65	−5.57	Low	Yes	Yes	No	Yes	Yes	Yes	2.58	0
**7b**	90.73	4.72	−5.51	Low	Yes	Yes	No	Yes	Yes	Yes	2.79	0
**7c**	77.84	5.94	−6.63	Low	Yes	Yes	No	Yes	Yes	Yes	2.75	0
**7d**	77.84	5.43	−5.60	Low	Yes	Yes	No	Yes	Yes	Yes	2.52	0
**7e**	103.86	4.39	−5.10	Low	Yes	Yes	No	Yes	Yes	Yes	2.61	0
**7f**	116.75	3.63	−4.62	Low	Yes	Yes	No	Yes	Yes	Yes	2.83	0
**7g**	110.96	3.99	−4.43	Low	Yes	Yes	No	Yes	Yes	Yes	2.80	0
**7h**	98.07	4.62	−4.74	Low	Yes	Yes	No	Yes	Yes	Yes	2.64	0
**7i**	98.07	4.63	−4.69	Low	Yes	Yes	No	Yes	Yes	Yes	2.59	0
**7j**	120.98	4.91	−5.24	Low	Yes	Yes	No	Yes	Yes	Yes	2.64	0
**7k**	120.98	4.86	−5.24	Low	Yes	Yes	No	Yes	Yes	Yes	2.67	0

**TABLE 4:** ADME‐Tox properties of synthesized bis‐oxadiazole scaffolds.

Entry	7a	7b	7c	7d	7e	7f	7g	7h	7i	7j	7k
**Absorption**											
Caco‐2	−4.744	−4.765	−4.811	−4.715	−4.787	−4.845	−4.79	−4.85	−4.79	−4.75	−4.79
MDCK	7.78E‐06	9.27E‐06	1.12E‐05	9.57E‐06	1.12E‐05	1.26E‐06	1.14E‐05	1.06E‐05	1.07E‐05	1.67E‐05	1.67E‐05
Pgp	—	—	—	—	—	—	—	—	—	—	—
HIA	—	—	—	—	—	—	—	—	—	—	—
PPB	76.9%	73.1%	77.9%	64.9%	91.3%	82.2%	79.6%	63.5%	80.4%	71.2%	67.5%
VDss	−0.07	0.01	−0.17	0.14	0.29	−0.07	0.13	−0.1	0.07	0.11	−0.001
**Metabolism**											
CYP1A2	No	No	No	No	No	No	No	No	No	No	No
CYP2C19	No	No	No	No	No	No	No	No	No	No	No
CYP2C9	No	No	No	No	No	No	No	No	No	No	No
CYP2D6	No	No	No	No	No	No	No	No	No	No	No
CYP3A4	Yes	Yes	Yes	Yes	Yes	Yes	Yes	Yes	Yes	Yes	Yes
**Excretion**											
CL plasma	3.294	3.056	3.782	3.50	4.779	3.943	4.559	4.963	4.620	2.490	2.712
T_1/2_	0.045	0.061	0.044	0.052	0.055	0.098	0.152	0.147	0.126	0.053	0.066
**Toxicity**											
hERG	0.008	0.005	0.002	0.006	0.006	0.005	0.014	0.011	0.007	0.069	0.084
AMES	0.056	0.073	0.091	0.096	0.087	0.219	0.066	0.044	0.058	0.916	0.897
Skin. Sen	0.403	0.237	0.329	0.399	0.136	0.220	0.172	0.227	0.204	0.438	0.443
Carcino.	0.903	0.906	0.913	0.908	0.914	0.888	0.888	0.898	0.903	0.914	0.907
Eye. cor.	0.035	0.044	0.061	0.055	0.087	0.071	0.044	0.095	0.116	0.09	0.071
HHT	0.799	0.834	0.593	0.744	0.863	0.901	0.847	0.777	0.771	0.89	0.886
PPB	101.77	101.30	101.57	101.30	100.76	99.67	100.25	101.00	101.09	101.78	101.77

Probability values: 0‐0.1 (—) & 0.1‐0.3 (–): excellent; 0.3‐0.5 (‐) & 0.5‐0.7(+): moderate; 0.7‐0.9 (++), and 0.9‐1.0 (+++): week.

**FIGURE 7:**
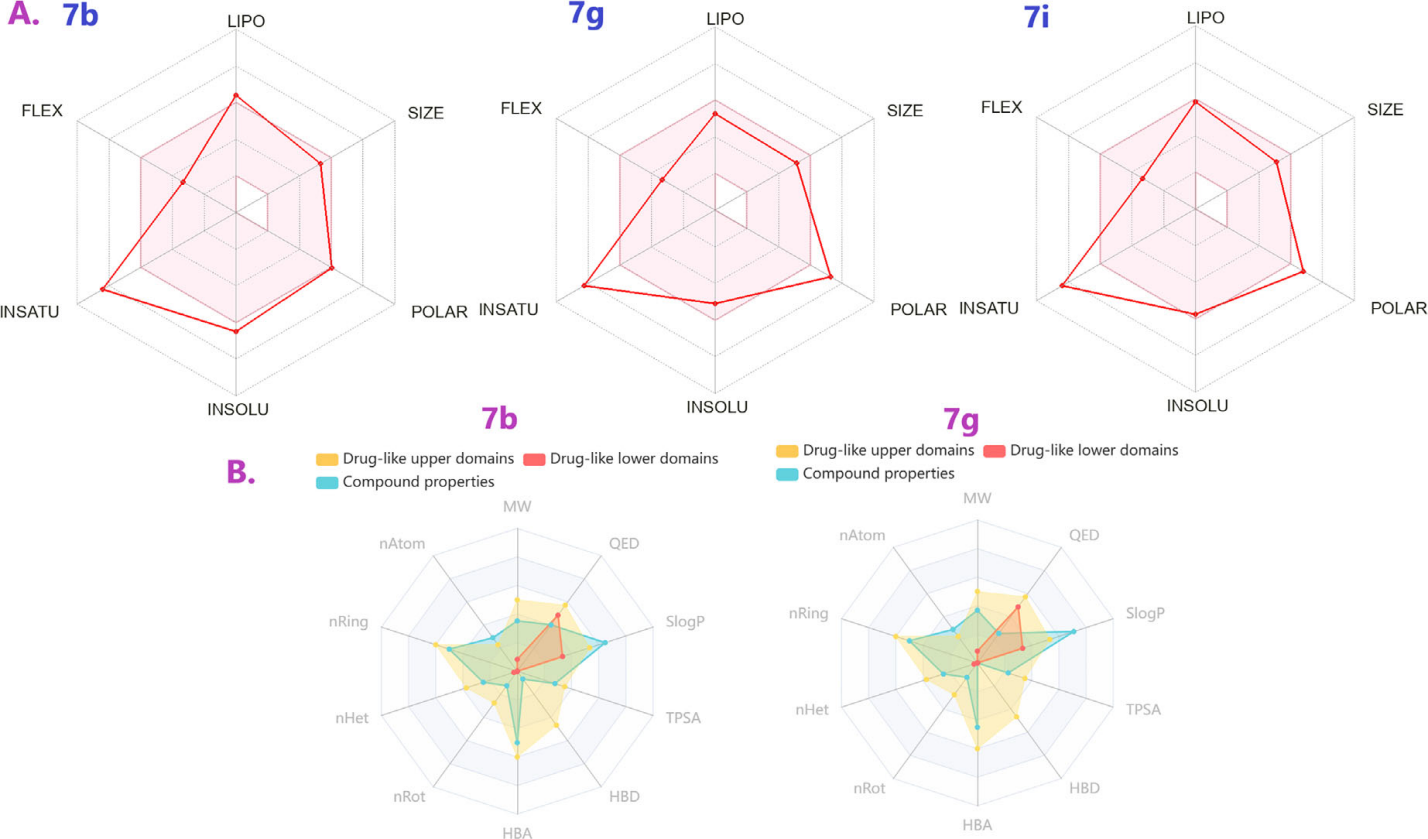
(A) Bioavailability radar plots of **7b, 7g,** and **7i; (**B) Druglike properties of compounds **7b, 7g,** and **7i**.

The CYP1A2, CYP2C9, CYP2C9, and CYP2D6 non‐inhibitors in the metabolic section were all inhibited by compounds **7a–7k**. All the targeted bis‐oxadiazole medications also inhibited the CYP3A4 inhibitor. However, none of the bis‐oxadiazole (**7a–7k**) are expected to operate as substrates of P‐glycoproteins (PGP) (blue dot), exhibit evidence of human intestinal absorption (HIA) (white), or pass the BBB (not in the yolk) according to the BOILED‐egg in Figure 8. Additionally, because the compounds were located in the grey portion of the egg, which is next to the white portion, they all showed low GI absorption. Either the new target compounds are toxic for the liver (H‐HT positive), with a predicted value of 0.3 to 0.7 (yellow/medium) for moderate toxicity and 0.7 to 1.0 (red/poor) for strong toxicity, or they are nontoxic for the liver (H‐HT negative), with a value of 0 to 0.3 (green/excellent). As a result, all of the chemicals were categorized as poor carcinogens with high predicted values between 0.888 and 0.914 and as substantial carcinogens. The chemicals that best meet the requirements of low AMES probability, moderate hERG liability, and acceptable rat oral acute toxicity are **7a** and **7h**. Compounds **7g** and **7h** show balanced acute oral toxicity, moderate hERG blockade risk, and low projected hepatotoxicity and mutagenicity. Likewise, **7b** and **7f** combine modest anticipated cardiotoxicity and systemic toxicity with negligible AMES risks. **7b** and **7c** had the highest hERG likelihood out of all the synthesized compounds and the chosen clinically approved medications. This information suggests that this medication may have a higher cardiotoxic risk, which would make it less ideal. The orange color radar represents the inactive cluster of physicochemical regions for optimal oral bioavailability, while the blue color radar displays the active cluster (hepatotoxicity, neurotoxicity, cardiotoxicity, immunotoxicity, cytotoxicity, clinical toxicity, GABA receptor, etc.) (Table 5 & Figure 9). Moreover, these compounds may have a limited therapeutic index and just a marginal membrane‐crossing efficiency. According to our research, the best‐docked oxadiazoles showed appropriate physicochemical, pharmacokinetic, and bioavailability in silico without any toxicity or carcinogenicity, indicating that they may be a potential new class of anticancer medications.

**FIGURE 8:**
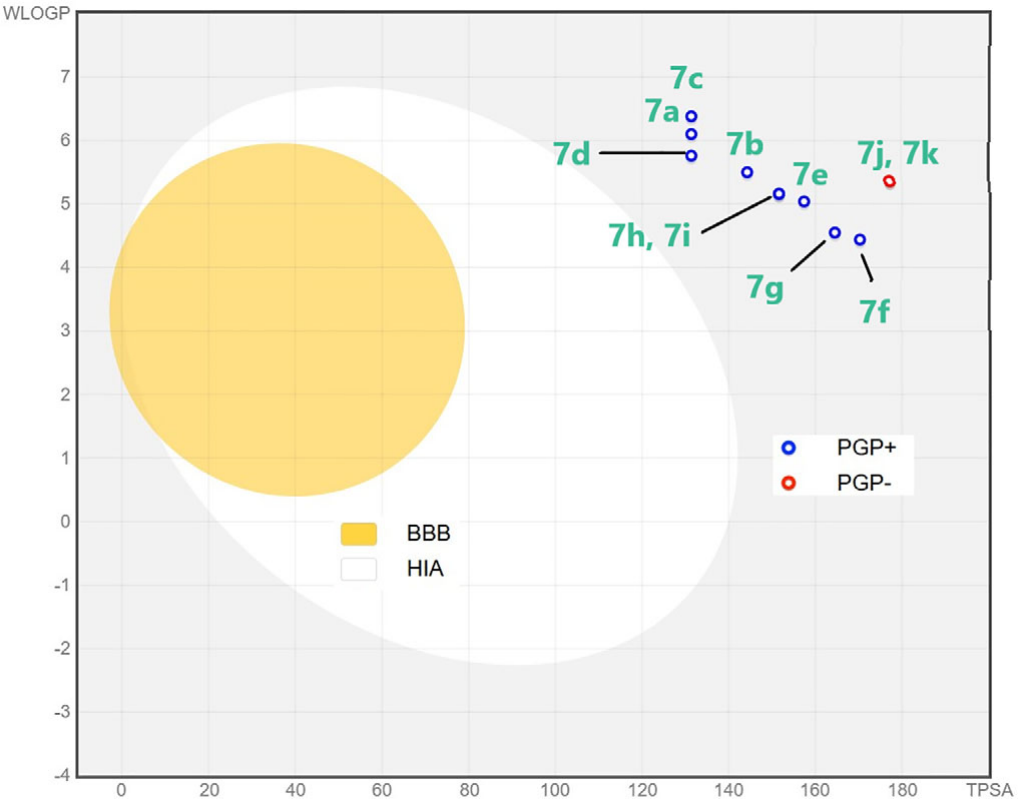
BOILED‐Egg predictive model of the bis‐oxadiazole scaffolds.

**TABLE 5:** Prediction of Toxicity of active compounds by using Protox‐II [58]^.^

Targets	Prediction/Probability		
	**7b**	**7g**	**7i**
Hepatotoxicity	Active/0.62	Active/0.60	Active/0.59
Neurotoxicity	Inactive/0.50	Active/0.52	Inactive/0.54
Nephrotoxicity	Inactive/0.54	Inactive/0.58	Inactive/0.50
Respiratory toxicity	Active/0.62	Active/0.64	Active/0.63
Cardiotoxicity	Inactive/0.82	Inactive/0.82	Inactive/0.76
Carcinogenicity	Active/0.51	Active/0.67	Active/0.50
Immunotoxicity	Inactive/0.97	Inactive/0.99	Inactive/0.97
Mutagenicity	Active/0.56	Active/0.56	Active/0.54
Cytotoxicity	Inactive/0.86	Inactive/0.84	Inactive/0.79
BBB‐barrier	Active/0.87	Active/0.82	Active/0.72
Clinical toxicity	Inactive/0.56	Inactive/0.56	Inactive/0.55
Androgen Receptor (AR)	Inactive/0.95	Inactive/0.91	Inactive/0.92
Estrogen Receptor Alpha (ER)	Inactive/0.75	Inactive/0.77	Inactive/0.82
GABA receptor	Inactive/0.79	Inactive/0.78	Inactive/0.80
Pregnane X receptor	Inactive/0.67	Inactive/0.76	Inactive/0.70

**FIGURE 9:**
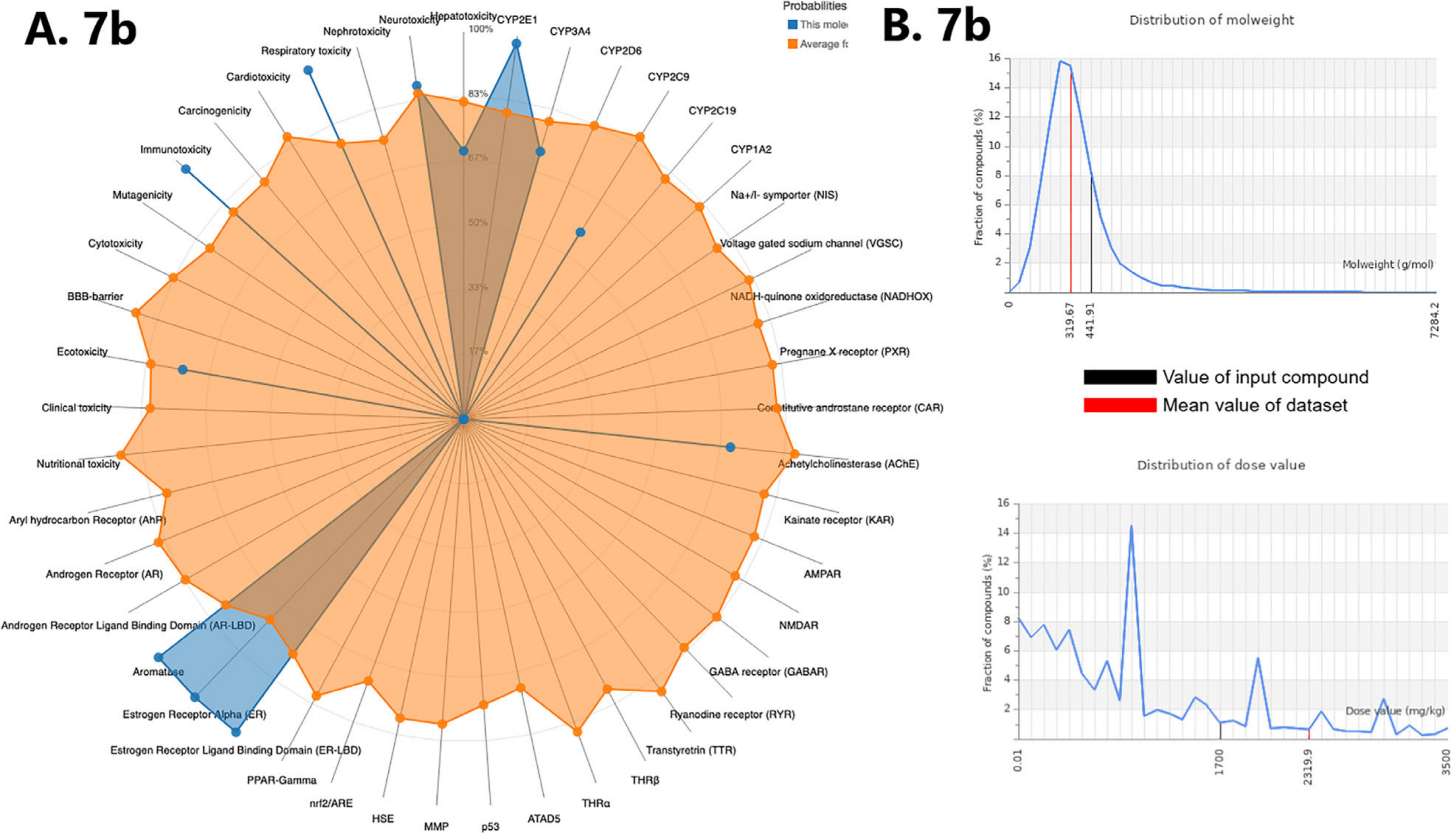
(A) The toxicity radar chart of oxadiazole **7a**. (B) Comparison of input compound **7b** with dataset compound.

## 3 Experimental

### 3.1 Materials and Methods

All the chemicals and reagents were purchased from Sigma‐Aldrich, Merck, and used without further purification. Uncorrected melting points were measured in open capillaries. Thin‐layer chromatography (TLC) using aluminum plates coated with silica gel from Merck Specialties Pvt. Ltd. was used to track the reaction's progress. The Gallenkamp melting point instrument (MP‐D) was used to observe melting points. ^1^H NMR spectra were registered on CDCl_3_ on a BRUKER AVANCE 400 MHz NMR Spectrometer, and the infrared (FTIR) spectra were recorded on a 470‐Shimadzu infrared spectrophotometer using the KBr disc made by pressed pellet technique, with ν_max_ expressed in cm^−1^. The solvent signal serves as the reference with respect to TMS (*δ* = 0) as the internal standard, and all chemical shifts (*δ*) are expressed in parts per million (ppm), while the coupling constants (*J*) are given in Hertz (Hz). ^13^C NMR spectra were determined by Bruker Avance II 400 NMR spectrometer in DMSO. The mass spectra (70 eV) were obtained on a Q‐TOF Micro mass (LC‐MS) Instrument. The molecular docking study was carried out using PyRx version v0.8, a computational tool that uses the Autodock 4.2 module. SwissADME (https://www.swissadme.ch/), ADMETlab 3.0 (https://admetlab3.scbdd.com/), and AdmetSAR 3.0 (https://lmmd.ecust.edu.cn/admetsar3/predict.php) was thus used to predict the target compounds' physicochemical and pharmacokinetic characteristics. Using the Gaussian 09 quantum chemical calculations and B3LYP functional with 6‐31G(d) basic set and Geometry optimization performed in gas phase (PCM model).

### 3.2 Synthesis of Intermediate Compound **5**

Addition of 1‐(2‐chlorophenyl)ethan‐1‐one (**1**) (0.025 mol, 4 g) and sodium hydride (0.077 mol, 1.90 g) to be jointly stirred in DMF (40 mL). After cooling, methyl 2‐mercaptoacetate (0.048 mol, 4.16 g) was progressively added, and the reaction mixture was heated under reflux for 4 h. After completion, the reaction was followed by evaporation of the solvent, then washing with acetone, to obtain the cyclized product (**5**) (white solid, yield: 80%). The remaining intermediate compounds, up to **4,** were prepared by using the previous literature [40] and used for further reaction without purification.

#### Synthesis of 2‐((5‐(3‐methylbenzo[*b*]thiophen‐2‐yl)‐1,3,4‐oxadiazol‐2‐yl)thio)acetonitrile (**5**)

A solution containing 0.068 molar amount (3.6 g) of 5‐(3‐methylbenzo[b]thiophen‐2‐yl)‐1,3,4‐oxadiazole‐2‐thiol **4** and selected 2‐bromoacetonitrile (0.020 mol, 3.15 g) was prepared in acetone (35 mL). The reaction mixture was then treated with a catalytic amount of potassium carbonate (0.002 mol, 1.90 g) and refluxed for 7 h. The purity of the product was determined by TLC, the solvent system used was n‐hexane: ethyl acetate (8:2). The reaction mixture was filtered and washed with dichloromethane/water, and the solvent was evaporated under reduced pressure. The obtained product was purified by flash column chromatography using hexane: ethyl acetate (8:2) to give the brownish product **5** (70%, 114°C–116°C).

#### Preparation of (*Z*)‐N'‐hydroxy‐2‐((5‐(3‐methylbenzo[*b*]thiophen‐2‐yl)‐1,3,4‐oxadiazol‐2‐yl) thio)acetimidamide (**6**)

In DCM (30 mL) containing triethylamine (0.0057 mol, 2.8 g), compound **5** (0.014 mol, 3.0 g) and hydroxylamine hydrochloride (0.039 mol, 2.8 g) were heated under rt for 12 h (as monitored by TLC). The light brownish coloured solid product was formed in a reaction mixture. Upon completion, the solvent was removed, and the crude product was washed with water and filtered and recrystallization from ethanol to yield the light‐brownish solid (73% yield, mp. 170°C–172°C).

### 3.3 General Procedure for the Preparation of Benzo[*b*]Thiophene‐1,3,4‐oxadiazole Linked to 1,2,4‐oxadiazole Molecules (**7a‐7k**)

Acetimidamide **6** (0.037 mol, 2.5 g), EDC.HCl (0.034 mol, 2.14 g) and the corresponding acid (*m*‐chlorobenzoic acid) (0.014 mol, 2.18 g) were charged in 25 mL of DCM at 15°C in a three‐necked flask for the experiment and it was agitated for 3–4 h at reflux temperature. Then, the acylated intermediate was refluxed in the presence of sodium acetate (0.011 mol, 0.92 g) in ethanol for 4‐5 h. Following TLC monitoring to verify acetimidamide **6** consumption (EtOAc: hexane; 2:8). The reaction mixture was put into 100 mL of dil. NaHCO_3_, brine solution (50 mL), and 150 mL of DCM before being moved into a separating funnel. Anhydrous Na_2_SO_4_ was used to dry the organic phase before it was filtered. After the target compound was purified using flash column chromatography, the solvent was extracted under vacuum, enabling its recovery.

### 3.4 Spectral Information

#### 5‐(3‐Chlorophenyl)‐3‐(((5‐(3‐methylbenzo[*b*]thiophen‐2‐yl)‐1,3,4‐oxadiazol‐2‐yl)thio)methyl)‐1,2,4‐oxadiazole **(7a)**

White solid; yields: 84%, mp: 180°C‐182°C; **^1^H‐NMR (400 MHz, DMSO−*d*_6_) *δ***: 8.02 (s, 1H, H_7_), 7.83‐7.91 (t, 2H, *J* = 9.0 Hz, H_3_, H_10_), 7.61 (d, 1H, *J* = 8.0 Hz, H_6_), 7.42‐7.52 (m, 4H, H_4,5,8,9_), 4.57 (s, 2H, H_2_), 2.27 (s, 3H, H_1_). **^13^C‐ NMR (100 MHz, DMSO‐*d*_6_) *δ***: 176.2, 162.4, 159.2, 143.5, 142.4, 141.2, 138.2, 136.4, 135.3, 132.2, 130.0, 128.8, 126.9, 125.2, 122.8, 119.6, 35.5, 15.8. Analysis for C_20_H_13_ClN_4_O_2_S_2_ (441.0310 [M+H]^+^, 442.0504 [M+2H]^+^): calcd, C 54.48; H 2.97; Cl 8.04; N 12.71; S 14.54; found, C 54.37; H 2.88; Cl 7.95; N 12.60; S 14.43.

#### 5‐(6‐Chloropyridin‐2‐yl)‐3‐(((5‐(3‐methylbenzo[*b*]thiophen‐2‐yl)‐1,3,4‐oxadiazol‐2‐yl)thio) methyl)‐1,2,4‐oxadiazole **(7b)**

Brown solid; yields: 74%, mp: 168°C‐170°C; **^1^H NMR (400 MHz, DMSO−*d*_6_) *δ***: 8.48 (d, 1H, *J* = 8.0 Hz, H_7_), 8.30 (t, 1H, *J* = 9.0 Hz, H_8_), 7.91 (d, 1H, *J* = 8.0 Hz, H_3_), 7.63‐7.68 (m, 2H, H_4,6_), 7.44 (t, 1H, *J* = 9.0 Hz, H_5_), 7.31 (d, 1H, *J* = 8.0 Hz, H_9_), 4.52 (s, 2H, H_2_), 2.28 (s, 3H, H_1_). **^13^C NMR (100 MHz, DMSO‐*d*_6_) *δ***: 164.8, 162.4, 159.2, 157.3, 149.3, 144.4, 142.4, 141.6, 139.2, 132.5, 131.6, 130.7, 129.1, 126.0, 125.0, 120.5, 35.7, 14.8. Analysis for C_19_H_12_ClN_5_O_2_S_2_ (442.9865 [M+H]^+^, 443.9798 [M+2H]^+^): calcd, C 51.72; H 2.83; Cl 8.11; N 15.93; S 14.60; found, C 51.64; H 2.74; Cl 8.02; N 15.85; S 14.51.

#### 5‐Mesityl‐3‐(((5‐(3‐methylbenzo[*b*]thiophen‐2‐yl)‐1,3,4‐oxadiazol‐2‐yl)thio)methyl)‐1,2,4‐oxadiazole (**7c**)

Gummy solid; yields: 67%, mp: 136°C‐138°C; **^1^H NMR (400 MHz, DMSO−*d*_6_) *δ***: 7.93 (d, 1H, *J* = 8.0 Hz, H_6_), 7.74 (d, 1H, *J* = 8.0 Hz, H_9_), 7.59 (t, 1H, *J* = 9.0 Hz, H_7_), 7.42 (t, 1H, *J* = 9.0 Hz, H_8_), 7.03 (s, 1H, H_5_), 4.50 (s, 2H, H_4_), 2.70 (s, 6H, H_3_), 2.32 (s, 3H, H_2_), 2.18 (s, 3H, H_1_). **^13^C NMR (100 MHz, DMSO‐*d*_6_) *δ***: 175.7, 162.4, 159.8, 144.8, 142.5, 141.2, 139.6, 137.2, 136.2, 132.0, 129.1, 126.5, 126.0, 125.0, 122.7, 120.3, 36.1, 22.7, 18.9, 14.8. Analysis for C_23_H_20_N_4_O_2_S_2_ (449.0477 [M+H]^+^): calcd, C 61.68; H 4.58; N 12.58; S 14.38; found, C 61.59; H 4.49; N 12.49; S 14.29.

#### 3‐(((5‐(3‐Methylbenzo[*b*]thiophen‐2‐yl)‐1,3,4‐oxadiazol‐2‐yl)thio)methyl)‐5‐(p‐tolyl)‐1,2,4‐oxadiazole **(7d)**

Gummy solid; yields: 69%, mp: 125°C‐127°C; **^1^H NMR (400 MHz, DMSO−*d*_6_) *δ***: 8.11 (d, 2H, *J* = 7.8 Hz, H_5_), 7.90 (d, 1H, *J* = 8.0 Hz, H_6_), 7.77 (d, 1H, *J* = 8.0 Hz, H_9_), 7.60 (t, 1H, *J* = 9.0 Hz, H_7_), 7.49 (t, 1H, *J* = 9.0 Hz, H_8_), 7.28 (d, 2H, d, *J* = 7.8 Hz, H_4_), 4.47 (s, 2H, H_3_), 2.34 (s, 3H, H_2_), 2.19 (s, 3H, H_1_). **^13^C NMR (100 MHz, DMSO‐*d*_6_) *δ***: 178.9, 162.7, 161.2, 145.6, 142.8, 141.2, 136.3, 132.9, 130.6, 129.2, 127.2, 126.2, 125.0, 124.2, 121.6, 36.4, 21.2, 15.2. Analysis for C_21_H_16_N_4_O_2_S_2_ (421.8810 [M+H]^+^): calcd, C 59.98; H 3.84; N 13.32; S 15.25; found, C 59.87; H 3.73; N 13.20; S 15.16.

#### 4‐(3‐(((5‐(3‐Methylbenzo[*b*]thiophen‐2‐yl)‐1,3,4‐oxadiazol‐2‐yl)thio)methyl)‐1,2,4‐oxadiazol‐5‐yl)aniline (**7e**)

Light brown solid; yields: 77%, mp: 178°C‐180°C; **^1^H NMR (400 MHz, DMSO−*d*_6_) *δ***: 8.18 (d, 1H, *J* = 8.0 Hz, H_6_), 8.01 (d, 1H, *J* = 8.0 Hz, H_9_), 7.74 (d, 2H, *J* = 7.8 Hz, H_5_), 7.52 (t, 1H, *J* = 9.0 Hz, H_7_), 7.43 (t, 1H, *J* = 9.0 Hz, H_8_), 6.68 (d, 2H, *J* = 7.8 Hz, H_4_), 5.45 (brs, 2H, H_3_), 4.45 (s, 2H, H_2_), 2.32 (s, 3H, H_1_). **^13^C NMR (100 MHz, DMSO‐*d*_6_) *δ***: 176.9, 162.3, 159.7, 146.8, 142.6, 141.4, 139.7, 132.1, 129.8, 128.2, 125.8, 122.7, 120.7, 115.7, 114.0, 33.7, 14.3. Analysis for C_20_H_15_N_5_O_2_S_2_ (422.4275 [M+H]^+^): calcd, C 56.99; H 3.59; N 16.62; S 15.21; found, C 56.88; H 3.47; N 16.52; S 15.10.

#### 2‐(3‐(((5‐(3‐Methylbenzo[*b*]thiophen‐2‐yl)‐1,3,4‐oxadiazol‐2‐yl)thio)methyl)‐1,2,4‐oxadiazol‐5‐yl)pyridin‐3‐amine (**7f**)

Brown solid; yields: 79%, mp: 162°C‐164°C; **^1^H NMR (400 MHz, DMSO−*d*_6_) *δ***: 8.23 (d, 1H, *J* = 8.0 Hz, H_6_), 8.08 (d, 2H, *J* = 7.8 Hz, H_5_,_7_), 7.85 (d, 1H, *J* = 8.0 Hz, H_10_), 7.41 (t, 1H, *J* = 9.0 Hz, H_8_), 7.29 (t, 1H, *J* = 9.0 Hz, H_9_), 7.16 (d, 1H, *J* = 8.0 Hz, H_4_), 5.70 (brs, 2H, H_3_), 4.41 (s, 2H, H_2_), 2.29 (s, 3H, H_1_). **^13^C NMR (100 MHz, DMSO‐*d*_6_) *δ***: 167.6, 163.7, 157.9, 145.1, 142.7, 141.7, 137.6, 136.4, 135.1, 131.0, 128.8, 126.0, 124.3, 122.5, 35.1, 14.4. Analysis for C_19_H_14_N_6_O_2_S_2_ (423.1281 [M+H]^+^): calcd, C 54.12; H 3.43; N 19.97; S 15.26; found, C 54.02; H 3.34; N 19.89; S 15.18.

#### 6‐(3‐(((5‐(3‐Methylbenzo[*b*]thiophen‐2‐yl)‐1,3,4‐oxadiazol‐2‐yl)thio)methyl)‐1,2,4‐oxadiazol‐5‐yl)pyridin‐3‐ol (**7g**)

Brown solid; yields: 74%, mp: 172°C‐174°C; **^1^H NMR (400 MHz, DMSO−*d*_6_) *δ***: 10.25 (brs, 1H, H_6_), 8.34 (s, 1H, H_3_), 8.22 (d, 1H, *J* = 8.0 Hz, H_5_), 7.90 (d, 1H, *J* = 8.0 Hz, H_7_), 7.70 (d, 1H, *J* = 8.0 Hz, H_10_), 7.41‐7.51 (m, 3H, H_4,8,9_), 4.47 (s, 2H, H_2_), 2.21 (s, 3H, H_1_). **^13^C NMR (100 MHz, DMSO‐*d*_6_) *δ***: 165.9, 164.4, 163.2, 157.6, 149.2, 137.9, 137.0, 131.5, 129.0, 125.7, 125.6, 123.0, 120.4, 119.4, 118.1, 35.0, 14.5. Analysis for C_19_H_13_N_5_O_3_S_2_ (424.0627 [M+H]^+^): calcd, C 53.89; H 3.09; N 16.54; S 15.14; found, C 53.78; H 3.01; N 16.43; S 15.05.

#### 3‐(3‐(((5‐(3‐Methylbenzo[*b*]thiophen‐2‐yl)‐1,3,4‐oxadiazol‐2‐yl)thio)methyl)‐1,2,4‐oxadiazol‐5‐yl)phenol (**7h**)

Light yellow solid; yields: 63%, mp: 190°C–192°C; **^1^H NMR (400 MHz, DMSO−*d*_6_) *δ***: 9.59 (brs, 1H, H_7_), 8.09 (d, 1H, *J* = 8.0 Hz, H_8_), 7.92 (d, 1H, *J* = 8.0 Hz, H_11_), 7.71 (d, 1H, *J* = 8.0 Hz, H_3_), 7.48 (t, 1H, *J* = 9.0 Hz, H_9_), 7.32 (d, 1H, *J* = 8.0 Hz, H_4_), 7.21 (t, 1H, *J* = 9.0 Hz, H_10_), 7.02 (s, 1H, H_6_), 6.74 (d, 1H, *J* = 8.0 Hz, H_5_), 4.29 (s, 2H, H_2_), 2.37 (s, 3H, H_1_). **^13^C NMR (100 MHz, DMSO‐*d*_6_) *δ***: 176.8, 162.3, 158.0, 157.9, 142.8, 139.0, 138.6, 131.5, 128.9, 127.7, 127.6, 124.8, 123.2, 122.0, 120.1, 115.3, 111.9, 35.6, 14.6. Analysis for C_20_H_14_N_4_O_3_S_2_ (423.0656 [M+H]^+^): calcd, C 56.86; H 3.34; N 13.26; S 15.18; found, C 56.75; H 3.25; N 13.15; S 15.09.

#### 4‐(3‐(((5‐(3‐Methylbenzo[*b*]thiophen‐2‐yl)‐1,3,4‐oxadiazol‐2‐yl)thio)methyl)‐1,2,4‐oxadiazol‐5‐yl)phenol (**7i**)

Light yellow solid; yields: 66%, mp: 182°C‐184°C; **^1^H NMR (400 MHz, DMSO−*d*_6_) *δ***: 9.82 (brs, 1H, H_5_), 7.98 (d, 1H, *J* = 8.0 Hz, H_6_), 7.81 (d, 2H, *J* = 7.8 Hz, H_3_), 7.61 (d, 1H, *J* = 8.0 Hz, H_9_), 7.38 (t, 1H, *J* = 9.0 Hz, H_7_), 7.28 (t, 1H, *J* = 9.0 Hz, H_8_), 6.90 (d, 2H, *J* = 7.8 Hz, H_4_), 4.33 (s, 2H, H_2_), 2.23 (s, 3H, H_1_). **^13^C NMR (100 MHz, DMSO‐*d*_6_) *δ***: 176.1, 163.9, 162.8, 162.0, 141.9, 140.6, 130.5, 129.3, 126.8, 125.9, 122.6, 120.4, 119.3, 116.2, 34.1, 14.2. Analysis for C_20_H_14_N_4_O_3_S_2_ (423.1481 [M+H]^+^): calcd, C 56.95; H 3.43; N 13.34; S 15.04; found, C 56.86; H 3.34; N 13.26; S 15.18.

#### 3‐(((5‐(3‐Methylbenzo[*b*]thiophen‐2‐yl)‐1,3,4‐oxadiazol‐2‐yl)thio)methyl)‐5‐(4‐nitrophenyl)‐1,2,4‐oxadiazole (**7j**)

Yellow solid; yields: 81%, mp: 178°C‐180°C; **^1^H NMR (400 MHz, DMSO−*d*_6_) *δ***: 8.62 (d, 2H, *J* = 7.8 Hz, H_4_), 8.32 (d, 2H, *J* = 7.8 Hz, H_3_), 7.82 (d, 1H, *J* = 8.0 Hz, H_5_), 7.61 (d, 1H, *J* = 8.0 Hz, H_8_), 7.50 (t, 1H, *J* = 9.0 Hz, H_6_), 7.35 (t, 1H, *J* = 9.0 Hz, H_7_), 4.39 (s, 2H, H_2_), 2.30 (s, 3H, H_1_). **^13^C NMR (100 MHz, DMSO‐*d*_6_) *δ***: 175.6, 160.1, 157.7, 148.2, 143.8, 142.4, 137.5, 135.5, 134.7, 129.9, 128.6, 128.2, 124.5, 123.5, 120.2, 34.6, 14.9. Analysis for C_20_H_13_N_5_O_4_S_2_ (452.0316 [M+H]^+^): calcd, C 53.21; H 2.90; N 15.51; S 14.20; found, C 53.11; H 2.80; N 15.41; S 14.10.

#### 3‐(((5‐(3‐Methylbenzo[*b*]thiophen‐2‐yl)‐1,3,4‐oxadiazol‐2‐yl)thio)methyl)‐5‐(3‐nitrophenyl)‐1,2,4‐oxadiazole (**7k**)

Yellow solid; yields: 77%, mp: 198°C‐200°C; **^1^H NMR (400 MHz, DMSO−*d*_6_) *δ***: 8.51 (s, 1H, H_6_), 8.40 (d, 1H, *J* = 8.0 Hz, H_3_), 8.29 (d, 1H, *J* = 8.0 Hz, H_5_), 7.88 (d, 1H, *J* = 8.0 Hz, H_4_), 7.86 (t, 1H, *J* = 9.0 Hz, H_7_), 7.75 (d, 1H, *J* = 8.0 Hz, H_10_), 7.43 (t, 1H, *J* = 9.0 Hz, H_8_), 7.32 (t, 1H, *J* = 9.0 Hz, H_9_), 4.31 (s, 2H, H_2_), 2.35 (s, 3H, H_1_). **^13^C NMR (100 MHz, DMSO‐*d*_6_) *δ***: 176.9, 161.1, 159.8, 146.7, 142.3, 140.9, 139.2, 131.1, 129.6, 125.1, 124.0, 123.4, 122.7, 121.8, 120.3, 33.6, 14.3. Analysis for C_20_H_13_N_5_O_4_S_2_ (452.4855 [M+H]^+^): calcd, C 53.32; H 2.99; N 15.60; S 14.30; found, C 53.21; H 2.90; N 15.51; S 14.20. General experimental details of biological activity, docking studies and copies of ^1^H‐NMR, ^13^C‐NMR, and HRMS spectrums are included in supporting information (Figures S1‐S33).

## 4 Conclusion

In summary, eleven benzo[*b*]thiophene‐based bis‐oxadiazole derivatives **7a–7k** were synthesized from acetimidamide and various substituted acids under conventional conditions. The recently synthesized benzo[*b*]thiophene analogues were characterized through ^1^H/^13^C‐NMR and HRMS spectrometry. All compounds exhibited notable anticancer activity, with IC_50_ ranging from 5.1 ± 0.78 to 44.2 ± 1.33 µg/mL against human colorectal (HT‐29, HCT‐116, and Caco‐2) and breast (MDA‐MB‐231). In vitro assays demonstrated that these molecules exhibit stronger inhibitory activity against colon cancer HCT‐116, particularly compounds **7b** and **7g**, showing notable potency (IC_50_  = 5.1 ± 0.78 and 8.6 ± 1.33 µg/mL). Specifically, compound **7i** showed promising anti‐breast cancer effects against MDA‐MB‐231, with IC_50_ values of 9.5 ± 0.38 µg/mL. With the greatest negative Δ*G* (−9.92 kcal*mol^−1^), the ligand‐**7g** actually exhibited advantageous electrostatic, hydrophobic, H‐bond, and CHB interactions with the colorectal receptor's main active residues. For compounds **7a–7k**, in‐silico ADME/T profiling revealed favorable pharmacokinetic characteristics that adhered to Lipinski's Rule of Five and demonstrated prospective oral bioavailable medications. Additionally, DFT analysis identified electron‐rich and electron‐deficient areas on molecules, which were utilized in docking studies to compare polar and non‐polar interactions with kinase. Moreover, we anticipate that further research will lead to the development of more potent druggable agents.

## Author Contributions

**Nagaraju Madala** contributed to methodology, project administration, and writing – original draft. **Srilalitha Vinnakota** contributed to Investigation, review, and writing – original draft. **Vishal Sharma** contributed to investigation, supervision, writing – original draft, reviewed and edited the paper. All authors read and approved the final manuscript.

## Funding

This Research work is funded by VSN Labs to support Researches on higher education program (VSNCUP2024001), Medicinal Chemistry Division, VSN LABS, IDA Cherlapally, Phase‐V, Hyderabad, Telangana, India.

## Conflicts of Interest

The authors declare that they have no known competing financial interests or personal relationships that could have appeared to influence the work reported in this paper.

## Supporting information




**Supporting File 1**: cbdv71782‐sup‐0001‐SuppMat.pdf

## Data Availability

The data that support the findings of this study are available from the corresponding author upon reasonable request.
