# Supplementary material for: Development of Benzothiophene–Bearing Bis–oxadiazole Derivatives for Potential Anticancer Targets: Evaluation of DFT, ADME–Tox, and In Silico Docking
Source: Chem Biodivers. 2026 Oct 9;23(10):e71782. doi: 10.1002/cbdv.71782 (PMC13653123; doi:10.1002/cbdv.71782)
Supplement: Supplementary file 1 [file CBDV-23-e71782-s001.pdf]

Supporting Information

Anticancer -MTT assay

For the cytotoxicity screening of the *N*-acyl-hydrazone derivatives, three cell lines were employed: HT-29 (human colorectal adenocarcinoma), HCT-116 (human colorectal carcinoma), Caco-2 (human colorectal adenocarcinoma), and MDA-MB-231 (human breast carcinoma). The human cancer cell lines HT-29, HCT-116, Caco-2, and MDA-MB-231 used in this study were procured from the National Centre for Cell Science (NCCS), Pune, India.

Detailed information on the cell lines is as follows:

HT-29: Human colorectal adenocarcinoma cell line; species: Homo sapiens; sex: female; tissue of origin: colon/rectum adenocarcinoma; official cell line name: HT-29; RRID: CVCL_0320.

HCT-116: Human colorectal carcinoma cell line; species: Homo sapiens; sex: male; tissue of origin: colon carcinoma; official cell line name: HCT-116; RRID: CVCL_0291.

Caco-2: Human colorectal adenocarcinoma cell line; species: Homo sapiens; sex: male; tissue of origin: colorectal adenocarcinoma; official cell line name: Caco-2; RRID: CVCL_0025.

MDA-MB-231: Human breast adenocarcinoma cell line; species: Homo sapiens; sex: female; tissue of origin: breast adenocarcinoma; official cell line name: MDA-MB-231; RRID: CVCL_0062.

Both culture media were supplemented with 10% fetal bovine serum (FBS) and 1% penicillin-streptomycin mix, to formulate the complete culture medium. All cell lines were incubated at 37 °C in a humidified atmosphere containing 5% CO_2. Cells were initially seeded into sterile 96-well plates at a density of 1 x 10^4 cells/well and left to adhere for 24 hours in order to perform the cytotoxicity screening. Cells were then exposed to compounds **7a–k** at progressively higher concentrations (2.0–50 μg/mL) for a duration of 24 hours. Simultaneously, Etoposide was used as a reference medication and examined in the same experimental setup with the same concentration range. To ensure that the final DMSO concentration did not surpass 0.1% (v/v) in any treatment condition, stock solutions of all tested compounds and Etoposide were prepared fresh in dimethyl sulfoxide (DMSO) and diluted in complete culture medium to final concentrations of 32, 16.0, 8.0, 4.0, and 2.0 μg/mL. The culture media for the experimental control was changed when the therapy was administered. Cell viability was evaluated using the MTT test, which used 3-[4,5-dimethylthiazol-2-yl]-2,5-diphenyltetrazolium bromide, following a 24 h treatment period. In short, a new MTT solution (1 mg/mL) was added to each well, replacing the culture media that had been removed. Isopropanol was used to dissolve the formazan crystals that formed after the plates were incubated for four hours at 37 °C.

All experiments were performed in triplicate and repeated in three independent assays. Cell viability was calculated as a percentage relative to the untreated control using the formula Cell Viability (%) = (Abs treated/Abs control) x 100. Data were processed using GraphPad Prism 10.0 and presented as mean ± standard deviation (SD).

Density Functional Theory (DFT): GaussView 6.0

Density Functional Theory (DFT)[1] studies were performed using GaussView 6.0[2] as the graphical user interface for Gaussian calculations to evaluate the electronic properties of the compounds. Initially, the molecular structures were either drawn using the GaussView builder tool or imported from standard file formats such as .mol or .pdb. The constructed molecules were pre-optimized using the molecular mechanics option to remove steric strain. Subsequently, a full geometry optimization was carried out by selecting *Calculate → Gaussian Calculation Setup*, where the job type was set to Optimization and the computational method was chosen as DFT.[3] The B3LYP functional along with a suitable basis set such as 6-31G(d) was employed, and the

charge and spin multiplicity were assigned according to the molecular system. The calculations were then submitted and allowed to converge to the lowest energy geometry. After successful optimization, the optimized structures were further subjected to single-point energy calculations at the same level of theory to obtain accurate molecular orbital energies. Upon completion of the calculations, the Gaussian output (.log) files were opened in GaussView, and the molecular orbital information was accessed through *Results → Molecular Orbitals*.[4] The energies of the highest occupied molecular orbital (HOMO) and lowest unoccupied molecular orbital (LUMO) were recorded from the output file. Visualization of the HOMO and LUMO orbitals was performed using the *Surfaces and Contours* option to analyze electron density distribution and charge-transfer behavior. Finally, the HOMO–LUMO energy gap (ΔE) was calculated as the difference between the LUMO and HOMO energies ($\Delta E = {}^{E}LUMO - {}^{E}HOMO$).[5] The obtained values were converted from atomic units to electron volts using the conversion factor 1 Hartree = 27.2114 eV. The calculated energy gap was used to interpret the chemical stability, reactivity, and electronic characteristics of the studied compounds.

Docking study

The crystal structure of the Cyclophilin_CeCYP16-Like Domain of the Serologically Defined Colon Cancer Antigen 10 from Homo Sapiens", whose PDB ID is 2HQ6,[6] was retrieved from the PDB (Protein Data Bank) and used for molecular docking. It seems to have a 1.75 Å resolution and 185 amino-acid-residue protein of chain A. The molecular weight of the protein is 20547.87 kDa. AutoDockTools (ADT)1.5.6[7] was used to prepare the input files and removed water molecules. All Hydrogens were added and saved it in PDBQT format. These molecules were sketched in Tripos sybyl6.7[8] and minimized by using Gasteiger-Huckel charges and saved it in.mol2 format.[9] All the ligands being uploaded individually in.mol2 format, and torsion angles were set and saved it in PDBQT format. To confirm the protein-ligand complex's ability to bind, Autodock 4.2 was used.[10] After removing the water molecules and adding all of the hydrogens, the proteins 2HQ6 and ligands were uploaded separately. The protein was subsequently stored in pdbqt format. The torsion angles of a rigid protein and a flexible ligand were determined using the conventional docking method. The energetic map was calculated using a distance-dependent function of the dielectric constant, and grid dimensions of 60, 60, and 60 points were chosen. The amino acids found in the active site were chosen and centred in the x, y, and z directions. The grid spacing was 0.375 Å. For docking simulations, the Lamarckian genetic algorithm approach was used. The inhibition constant (Ki) for each ligand was computed from the estimated free energy of ligand binding after the optimal postures were examined for hydrogen bonding computations using Autodock 4.2, ligplot[11] and Biovia Discovery studio visualiser [Biovia, D.S. (2015) Discovery Studio Modeling Environment. Dassault Syst. Release, San Diego, 4]

References

[1] M. Farrokhnia, 'Density Functional Theory Studies on the Antioxidant Mechanism and Electronic Properties of Some Bioactive Marine Meroterpenoids: Sargahydroquionic Acid and Sargachromanol', *ACS Omega*, 5 (2020), 20382–20390.

[2] X. Ji, Y. Yang, 'Density Functional Theory Investigation on the Molecular Structure and Vibrational Spectra of Triclosan', *Advances in Infrared Spectroscopy*, 37 (2022), 23–32

[3] J. A. Pople, 'Quantum Chemical Models', *Angew. Chem.* 38 (1999), 13–14.

[4] M. S. Gordon, B. J. Stephen, J. A. Pople, W. J. Pietro, W. J. Hehre, ‘Self-consistent Molecular-orbital Methods. Small Split-valence Basis Sets for Second-row Elements’, *J. Am. Chem. Soc.* 104 (1982), 2797–2803.
[5] Z. Markovic, J. Dorovic, M. Dekic, M. Radulovi, S. Markovic, M. Ilic, ‘DFT Study of Free Radical Scavenging Activity of Erodiol’, *Chemical Papers*, 67 (2013), 1453–1461.
[6] T. L. Davis, J. R. Walker, V. Campagna-Slater, P. J. Finerty, R. Paramanathan, G. Bernstein, F. MacKenzie, W. Tempel, H. Ouyang, W. H. Lee, E. Z. Eisenmesser, S. Dhe-Paganon, ‘Structural and biochemical characterization of the human cyclophilin family of peptidyl-prolyl isomerases’, *PLoS Biol.* 8 (2010), e1000439.
[7] G. M. Morris, R.Huey, W. Lindstrom, M. F. Sanner, R. K. Belew, D. S. Goodsell, A.J. Olson, ‘AutoDock4 and AutoDockTools4: Automated docking with selective receptor flexibility’, *J. Comput. Chem.* 30 (2009), 2785-2791.
[8] G.M. Morris, D.S. Goodsell, R.S. Halliday, R. Huey, W.E. Hart, R.K. Belew, A.J. Olson, ‘Automated docking using a Lamarckian genetic algorithm and an empirical binding free energy function’, *J. Comput. Chem*. 19 (1998), 1639-1662.
[9] S. Forli, R. Huey, M. E. Pique, M. F. Sanner, D. S. Goodsell, A. J. Olson, ‘Computational protein-ligand docking and virtual drug screening with the AutoDock suite’, *Nat Protoc*. 1 (2016), 905-19.
[10] R. A. Laskowski, J. Jabłońska, L. Pravda, R. S. Varekova, J. M. Thornton, ‘PDBsum: Structural summaries of PDB entries’, *Protein Sci*. 27 (2018), 129-134.
[11] R. A. Laskowski, M. B. Swindells, ‘LigPlot+: multiple ligand-protein interaction diagrams for drug discovery’, *J Chem Inf Model* 51 (2011), 2778-86.

Supporting Spectral Information [^{1}H/^{13}C-NMR & HRMS] (Figure S1-S33)

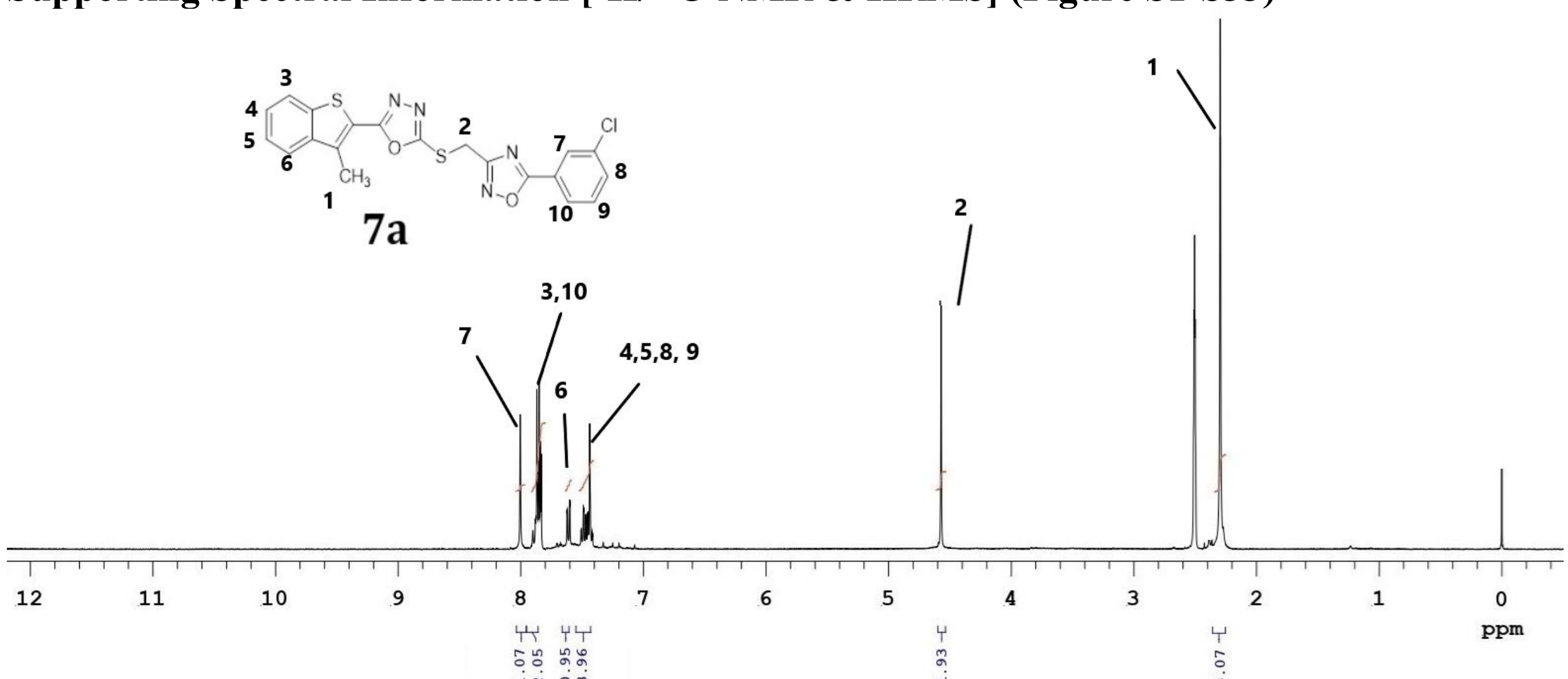

Figure S1. ^{1}H NMR of **7a**

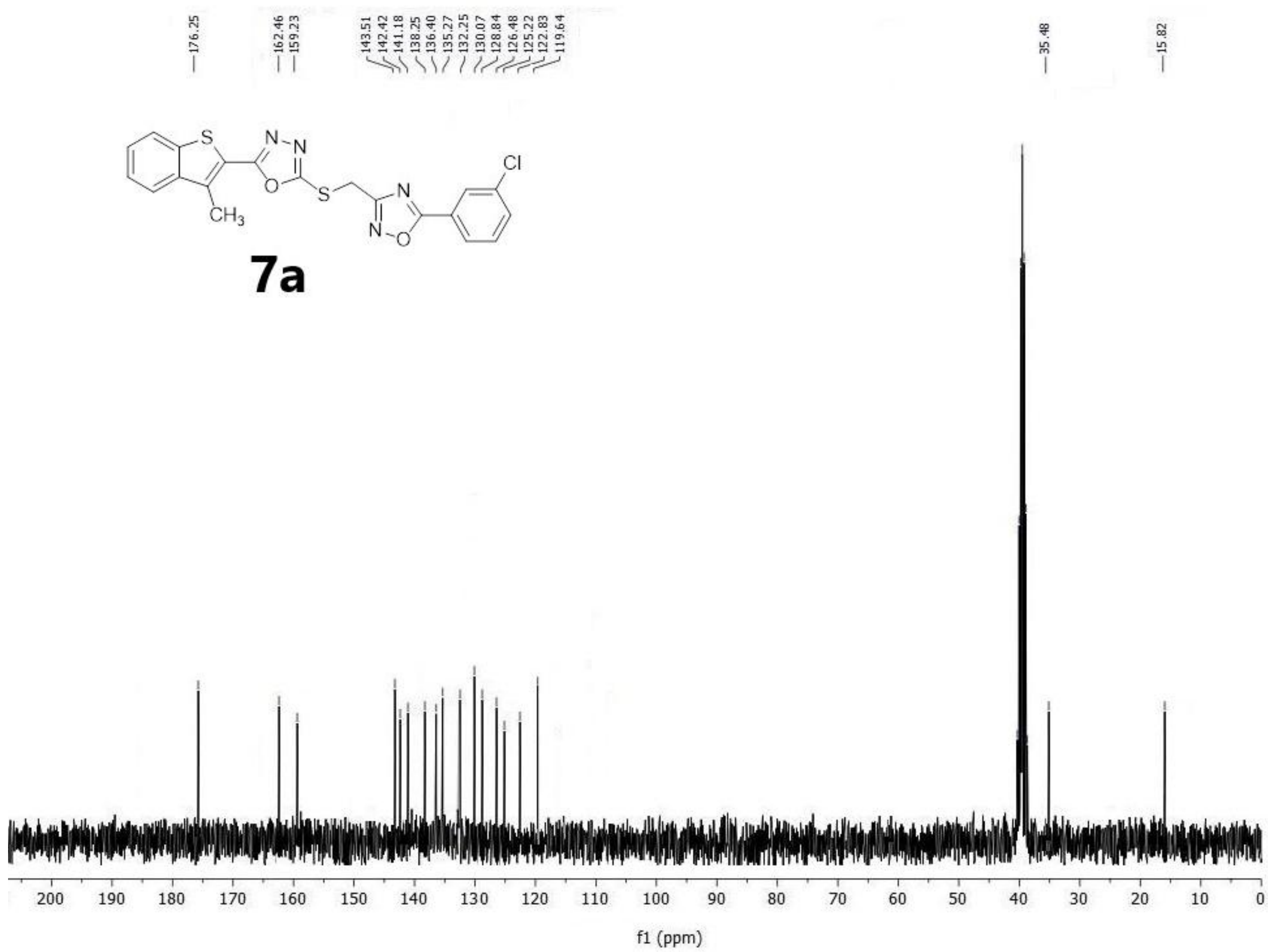

Figure S2. ^{13}C NMR of **7a**

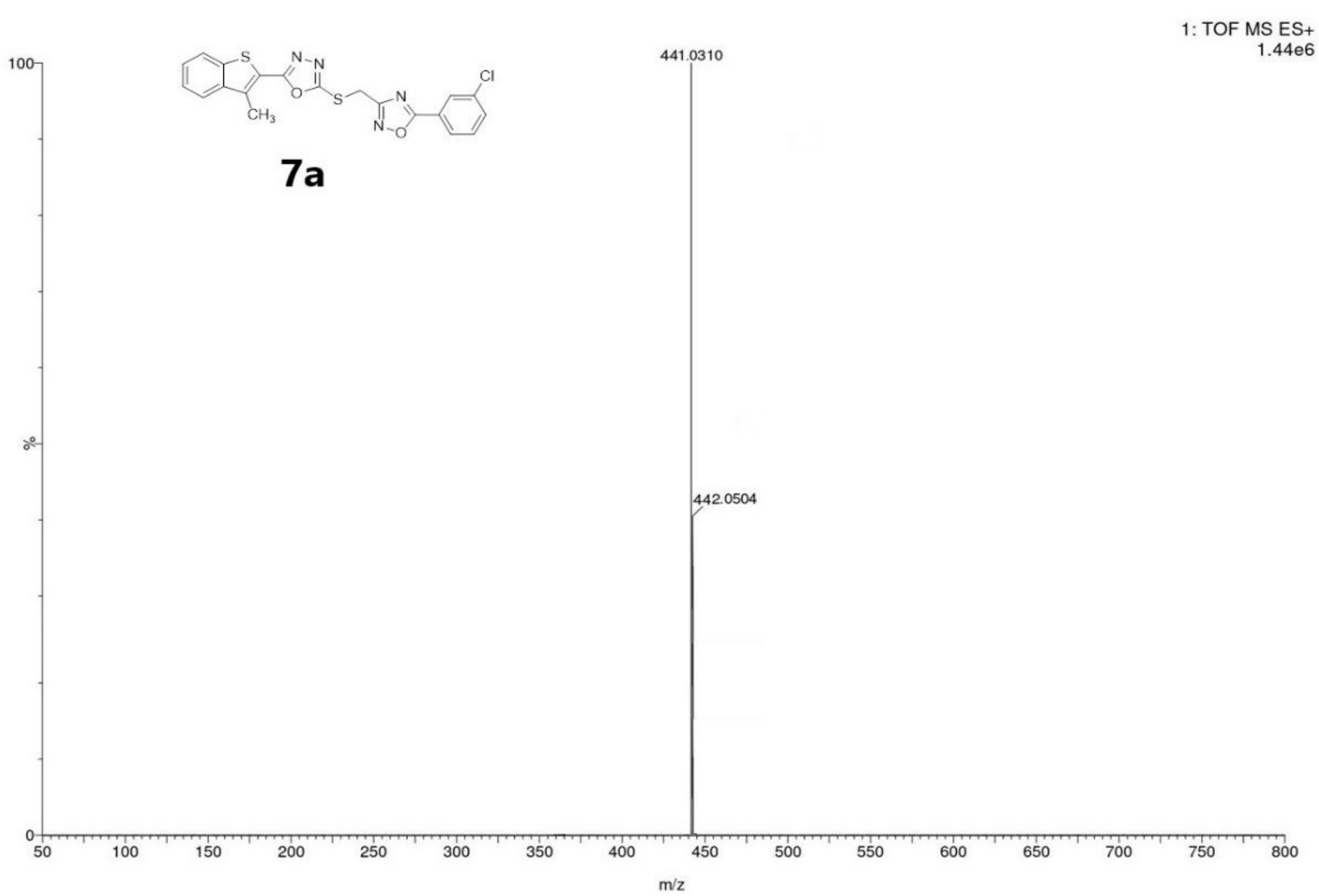

Figure S3. HRMS of **7a**

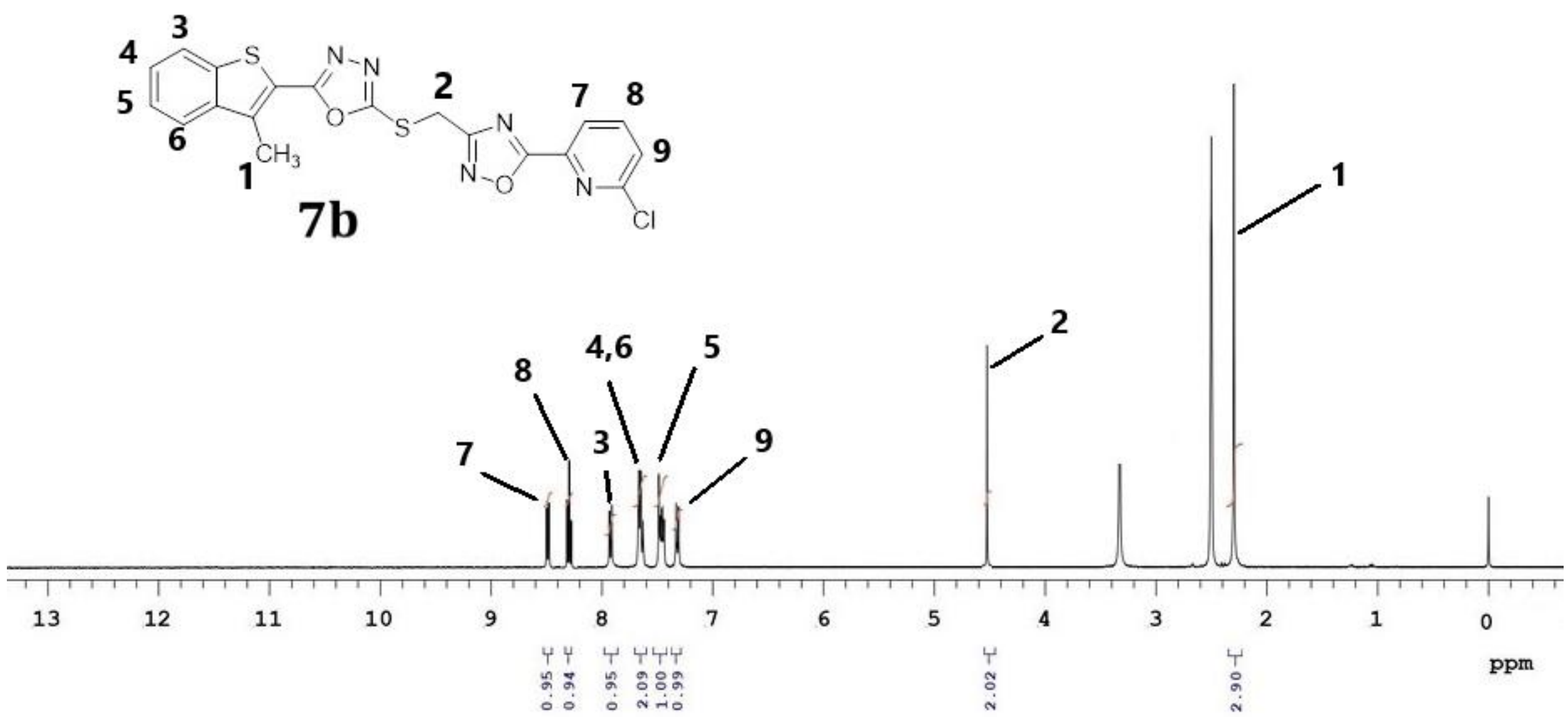

Figure S4. ^{1}H NMR of **7b**

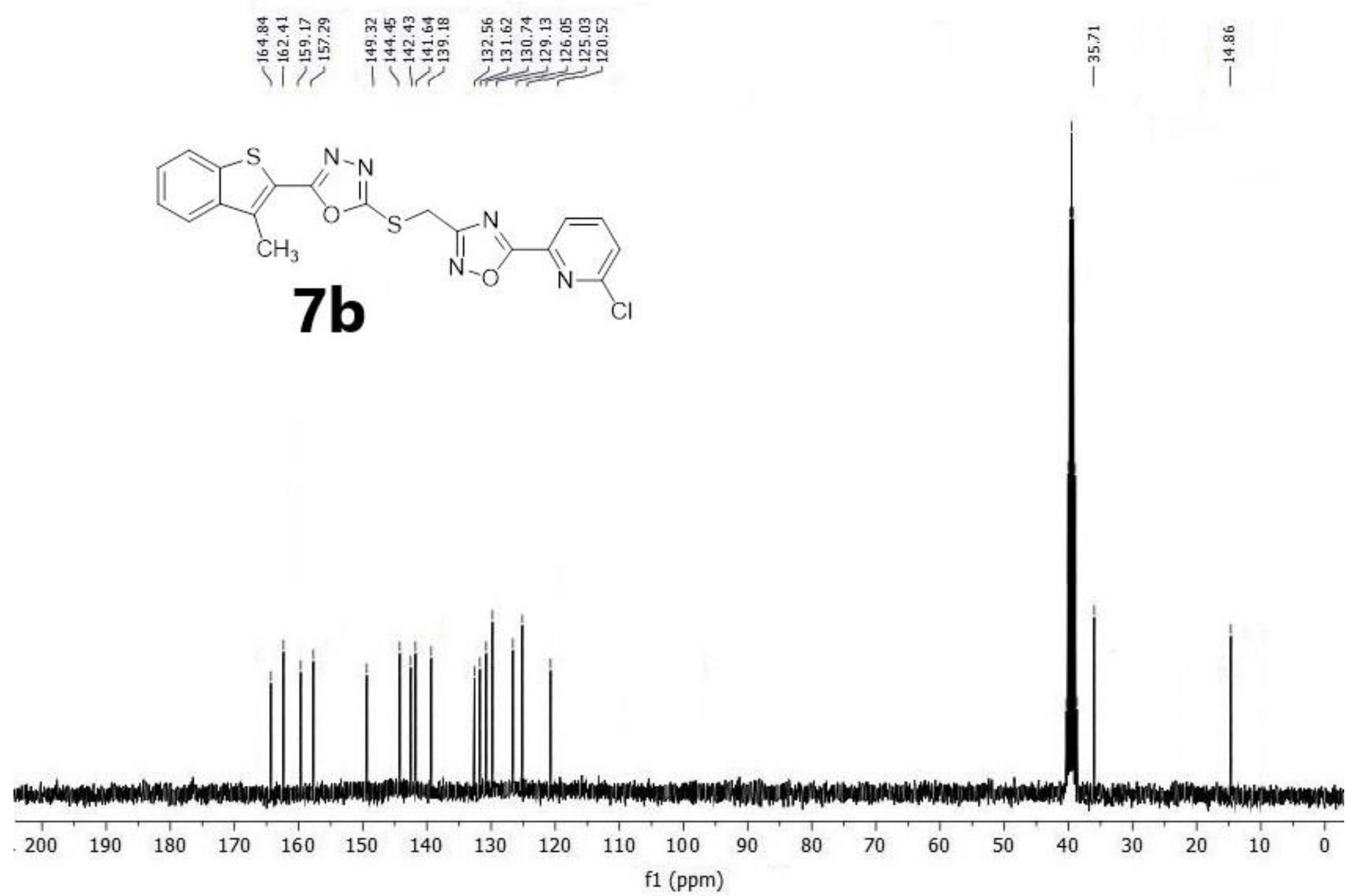

Figure S5. ^{13}C NMR of **7b**

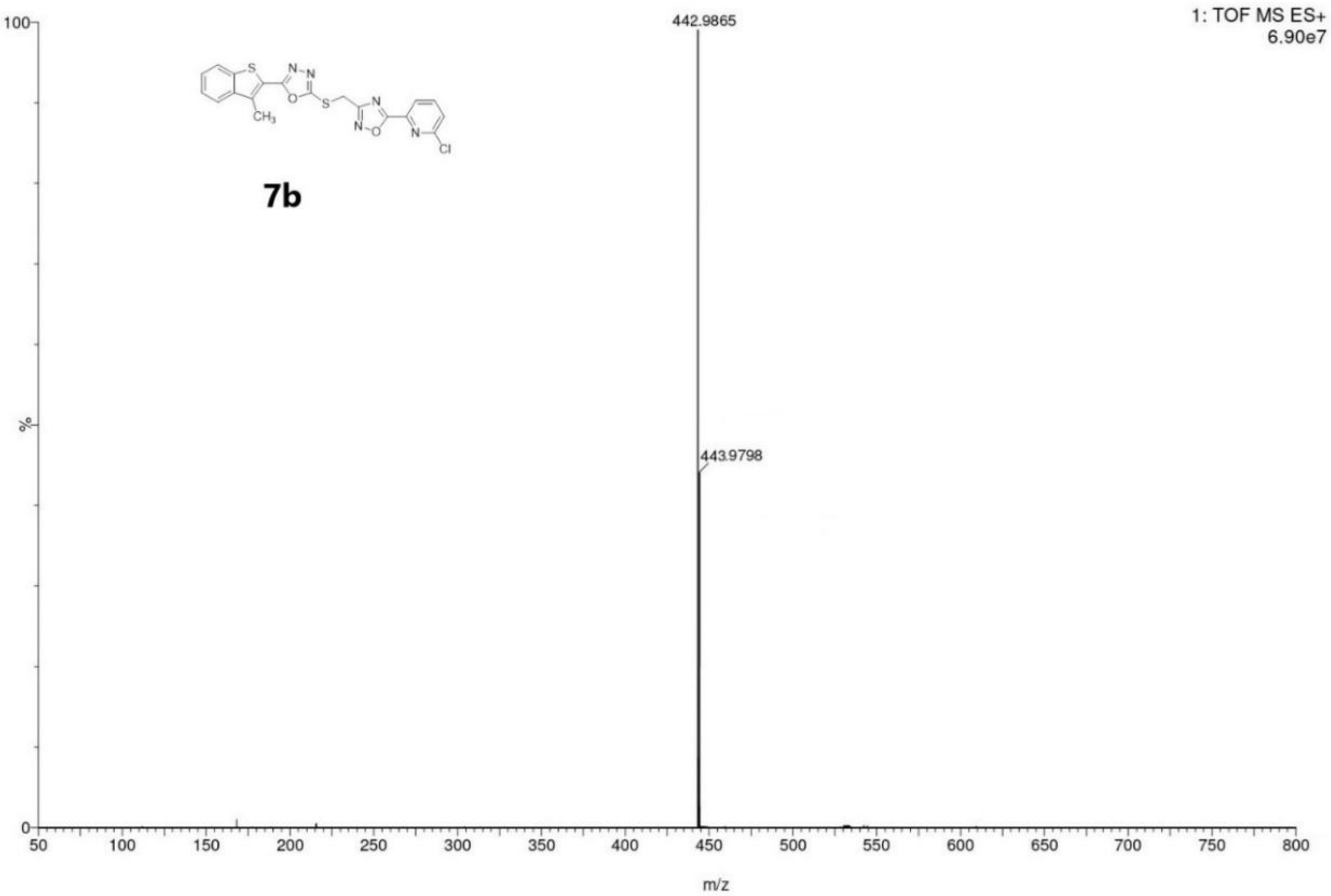

Figure S6. HRMS of **7b**

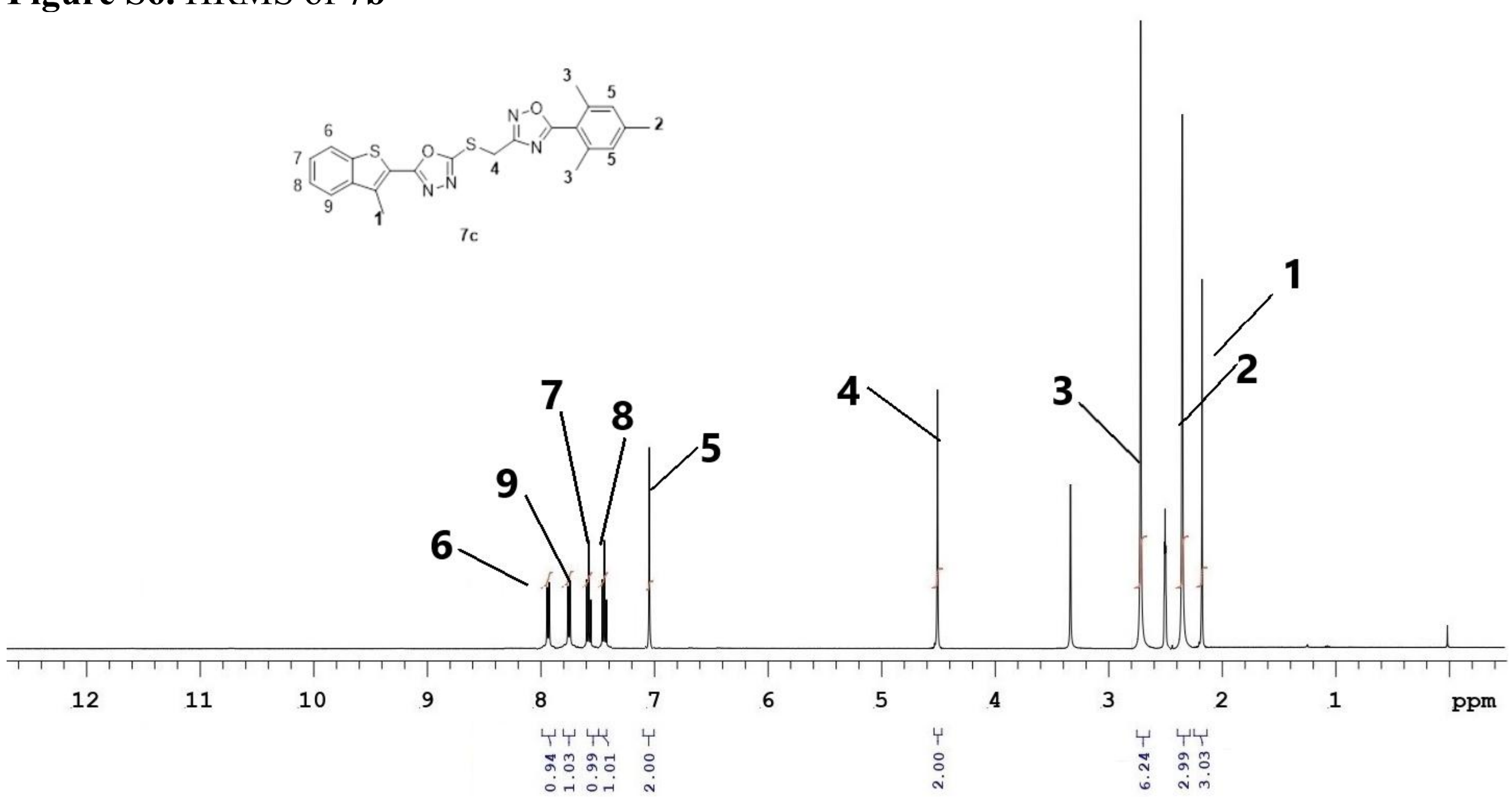

Figure S7. ^{1}H NMR of **7c**

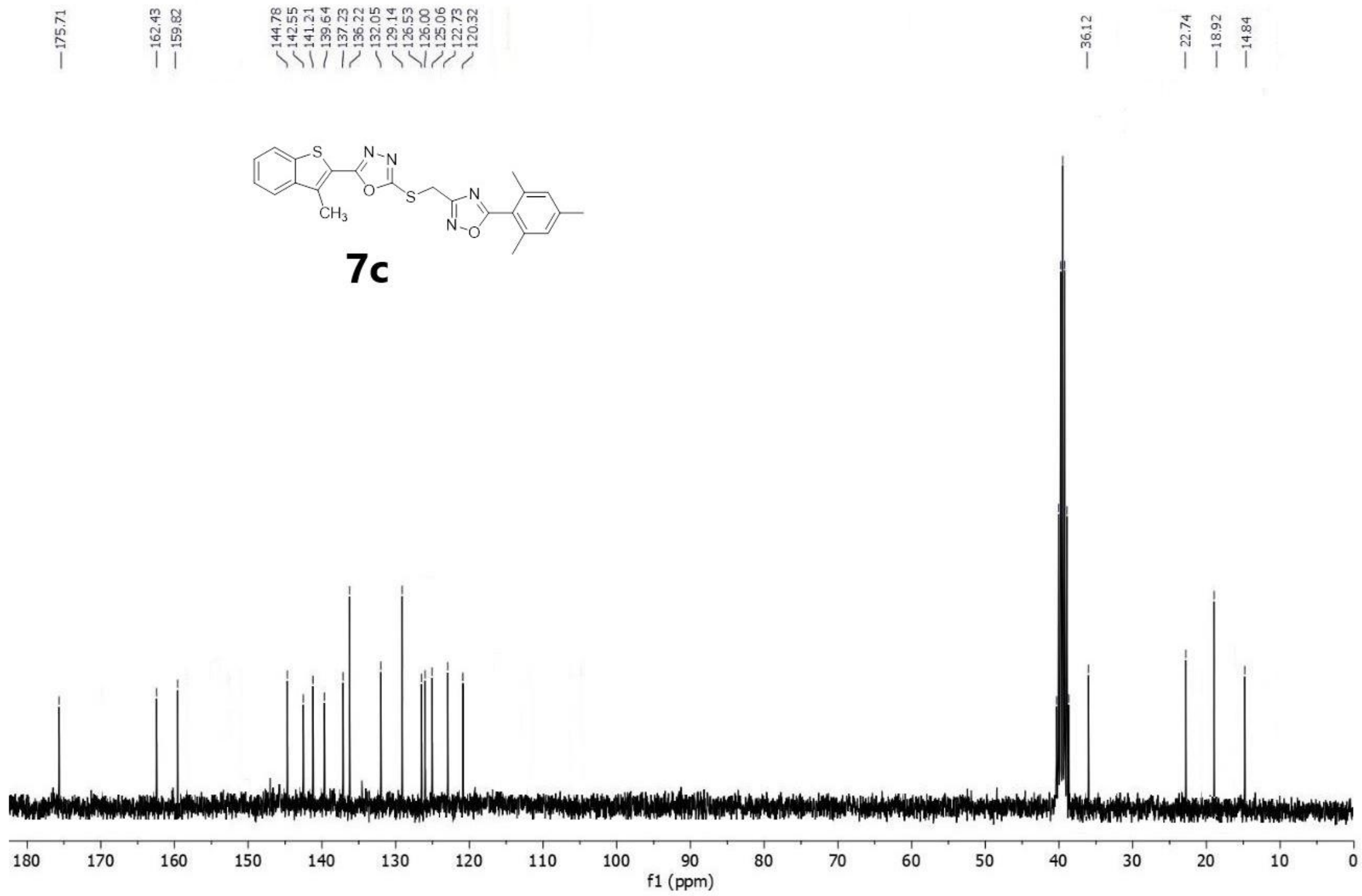

Figure S8. ^{13}C NMR of **7c**

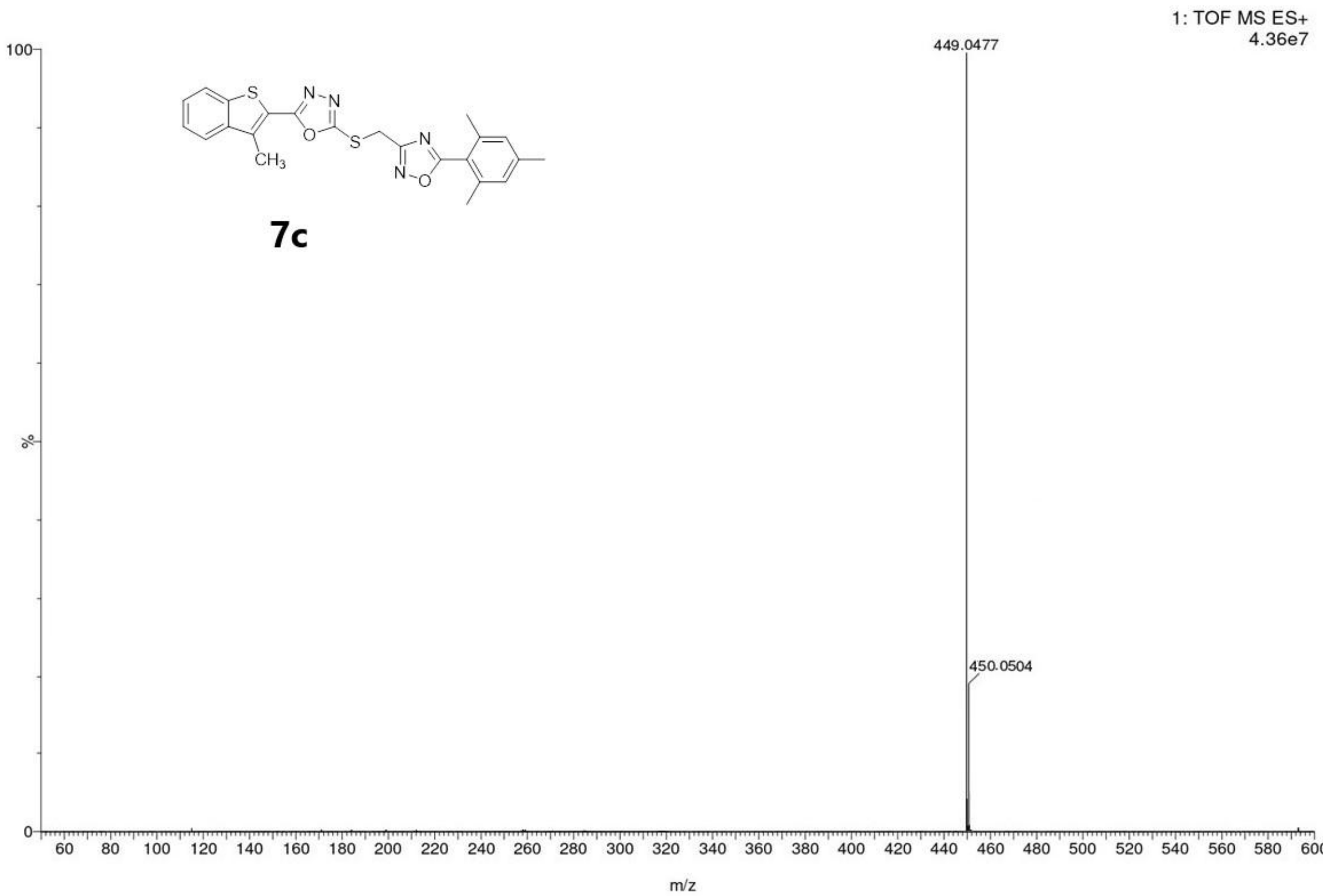

Figure S9. HRMS of **7c**

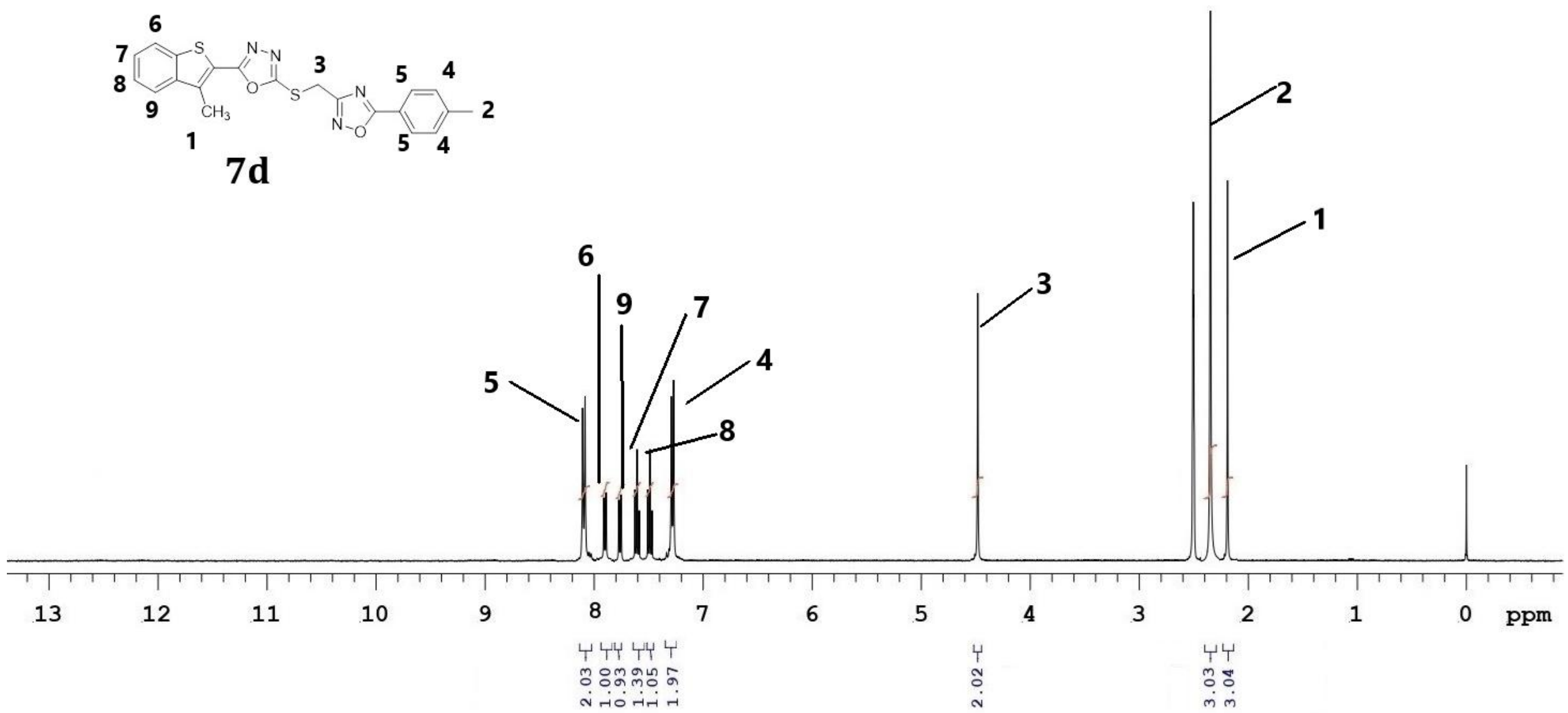

Figure S10. ^{1}H NMR of **7d**

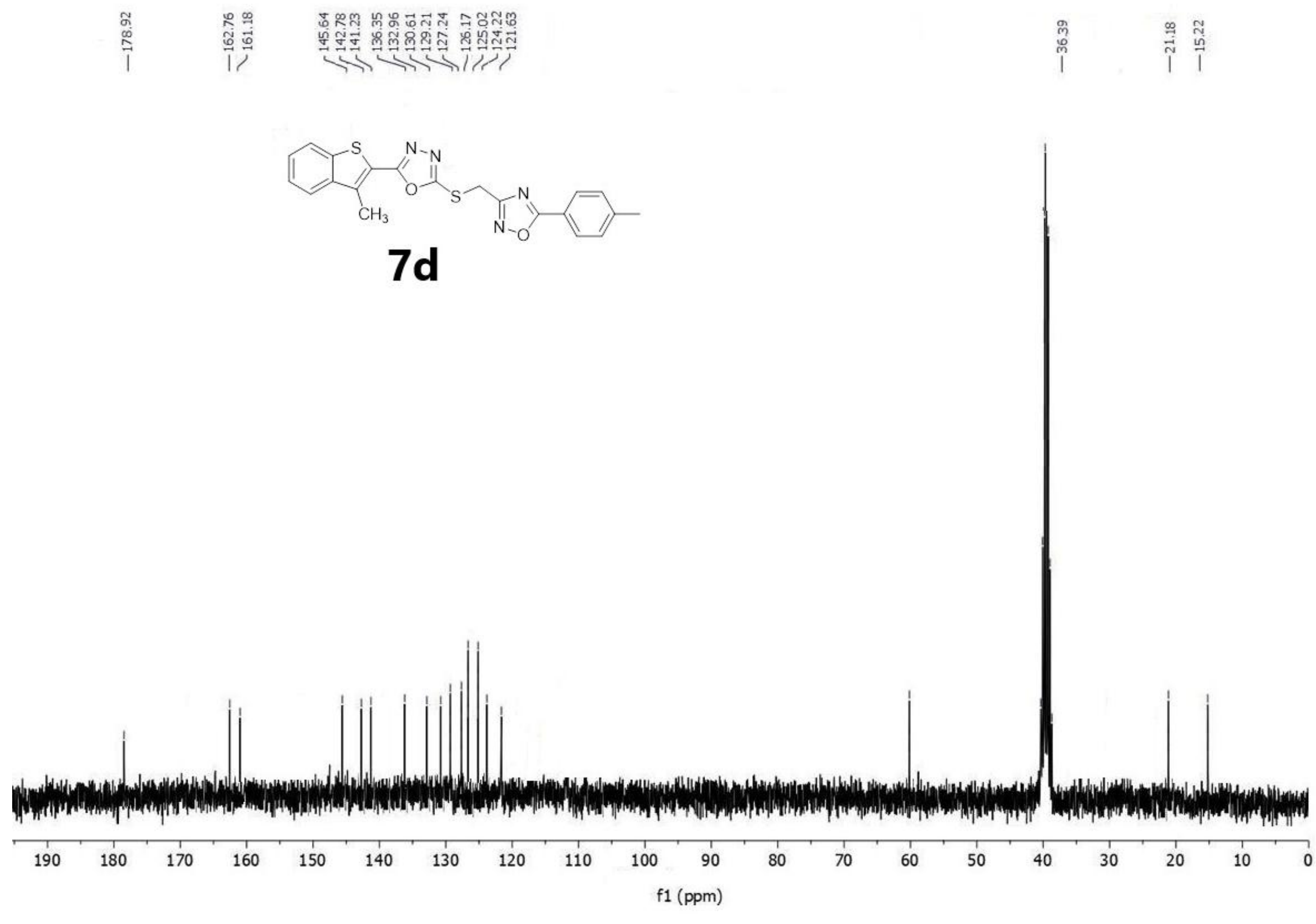

Figure S11. ^{13}C NMR of **7d**

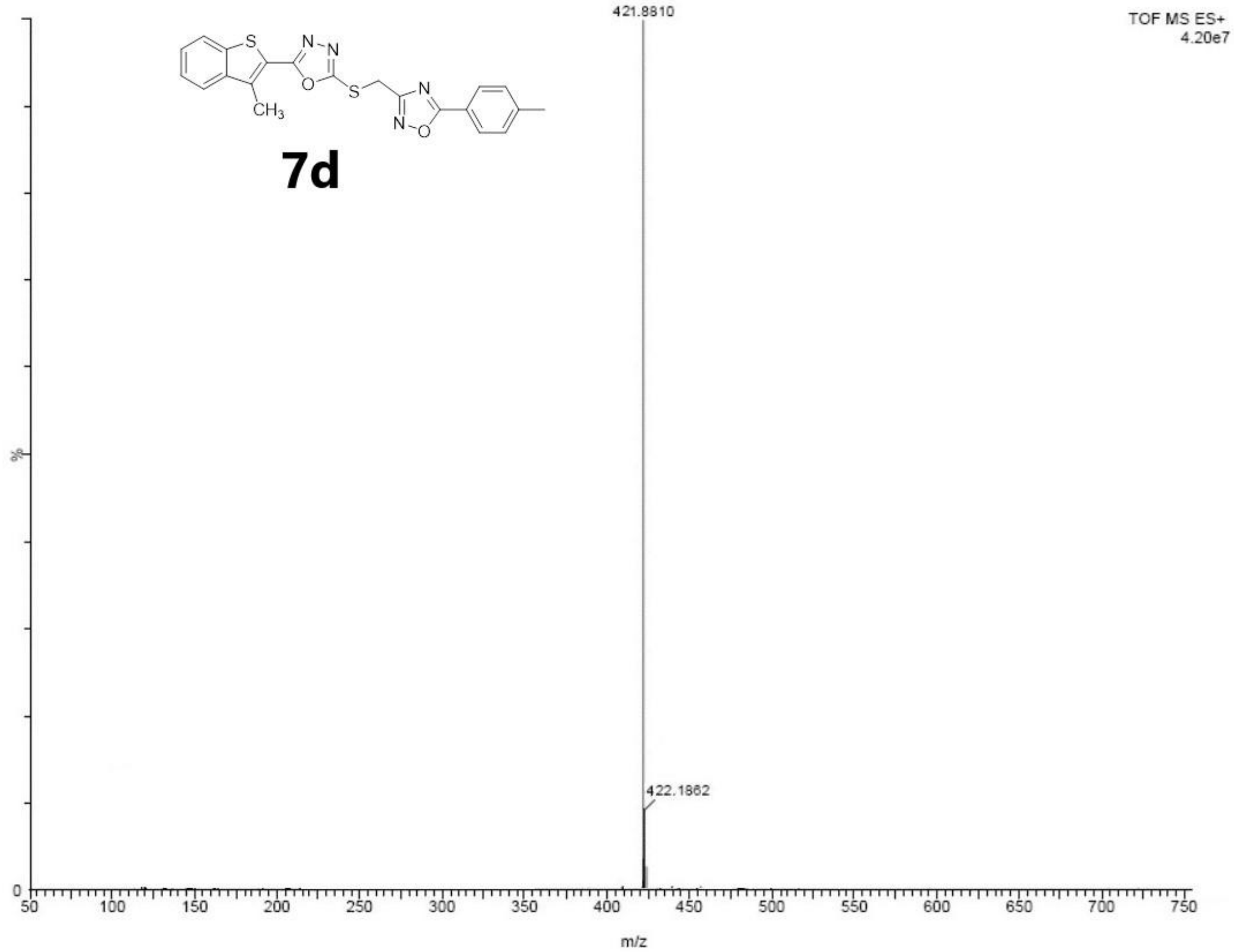

Figure S12. HRMS of **7d**

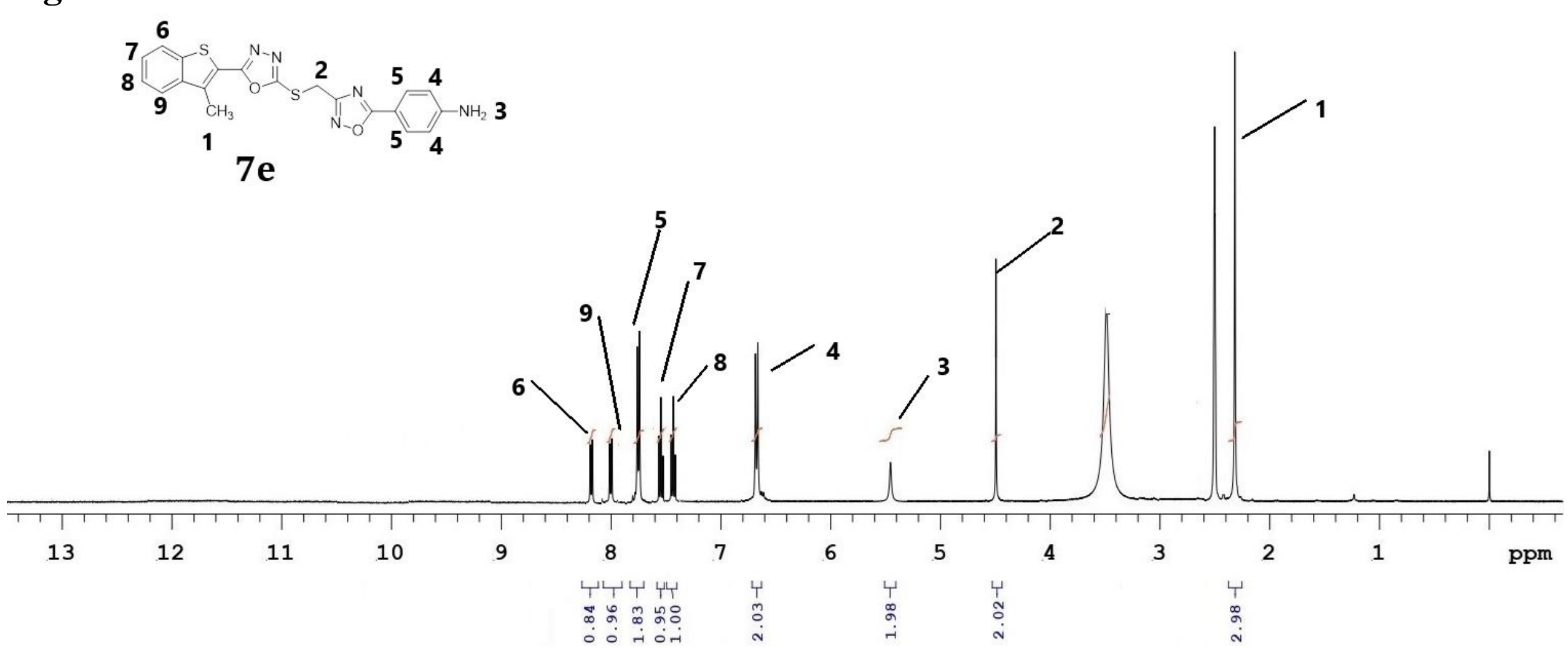

Figure S13. ^{1}H NMR of **7e**

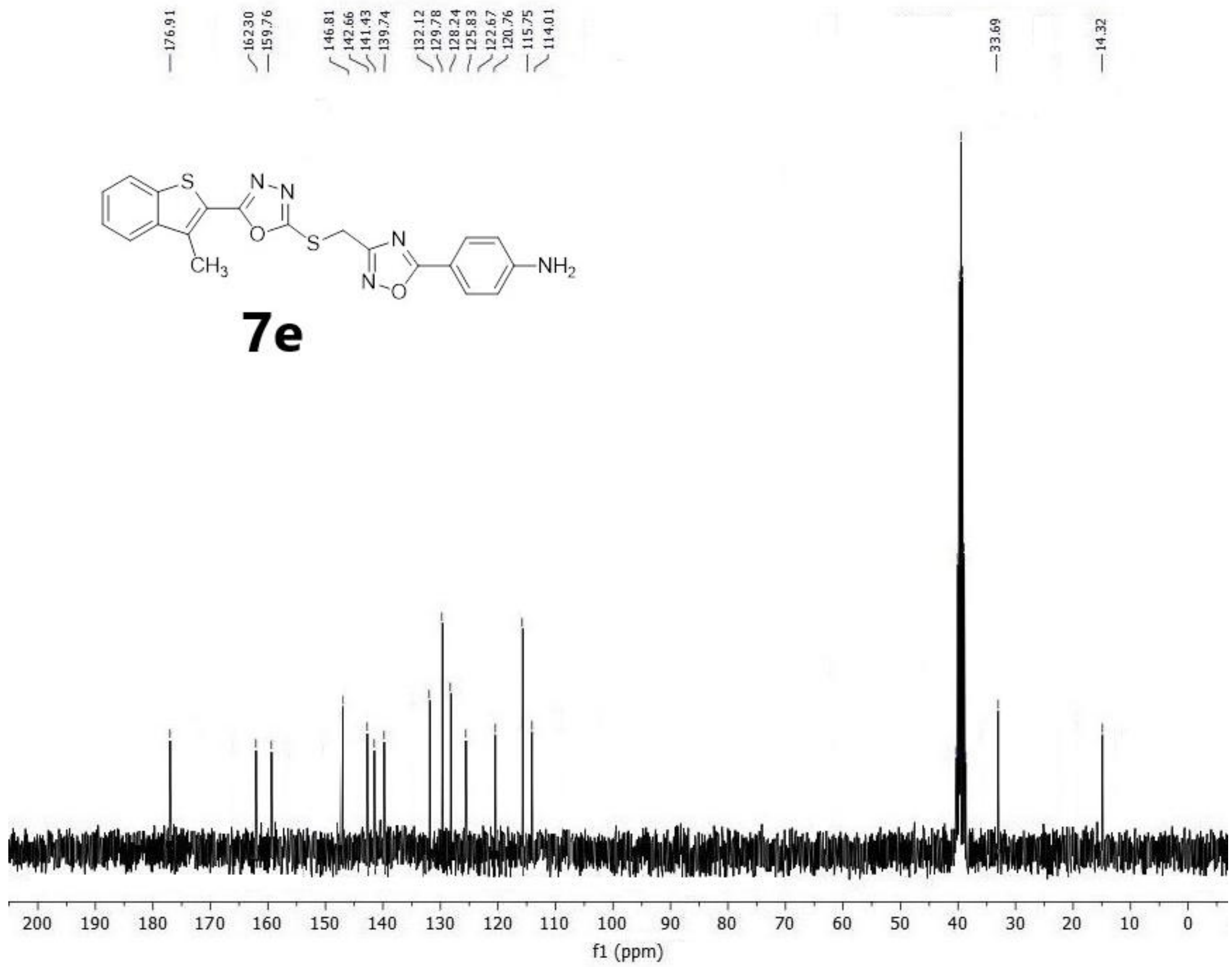

Figure S14. ^{13}C NMR of **7e**

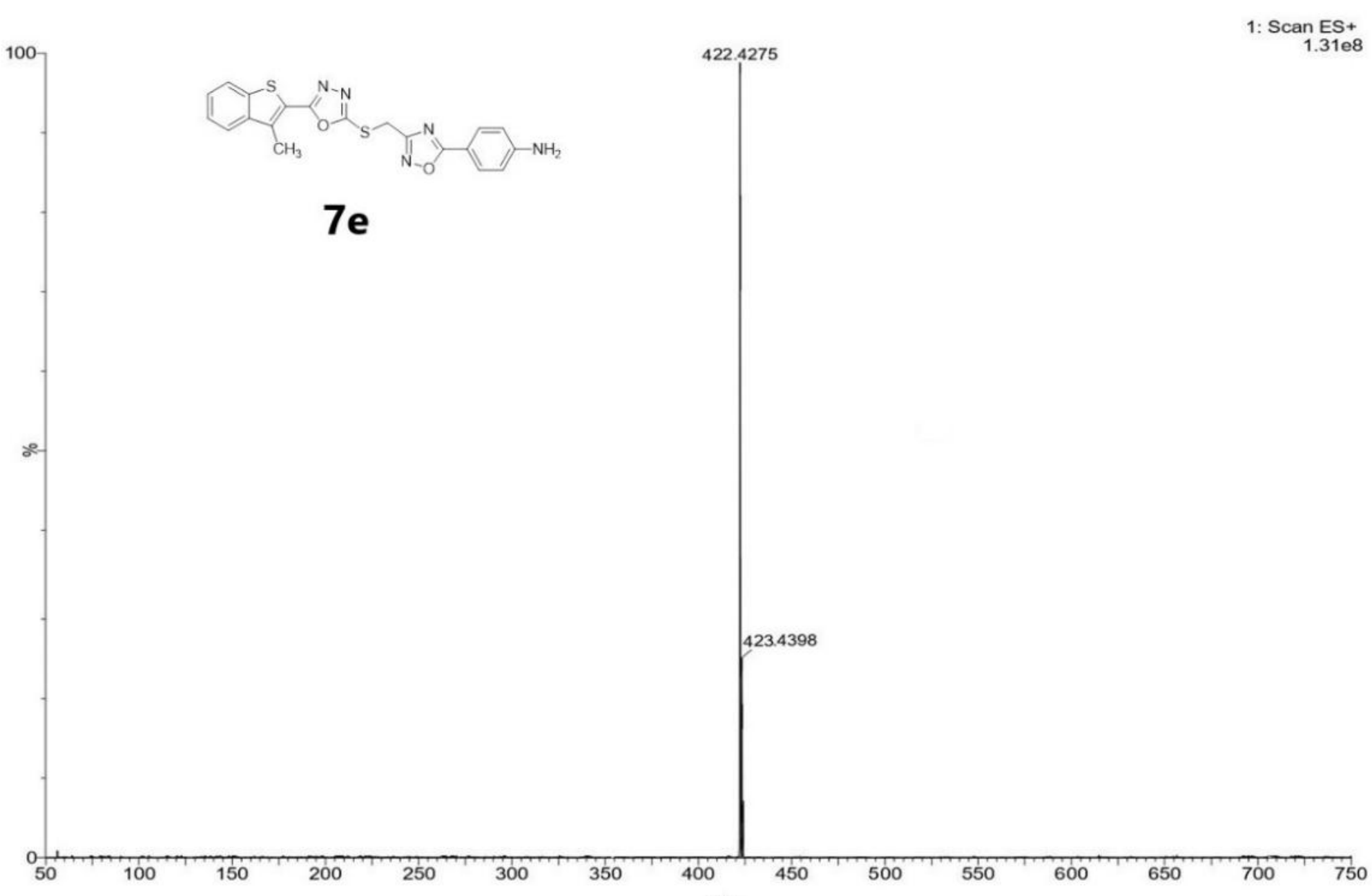

Figure S15. HRMS of **7e**

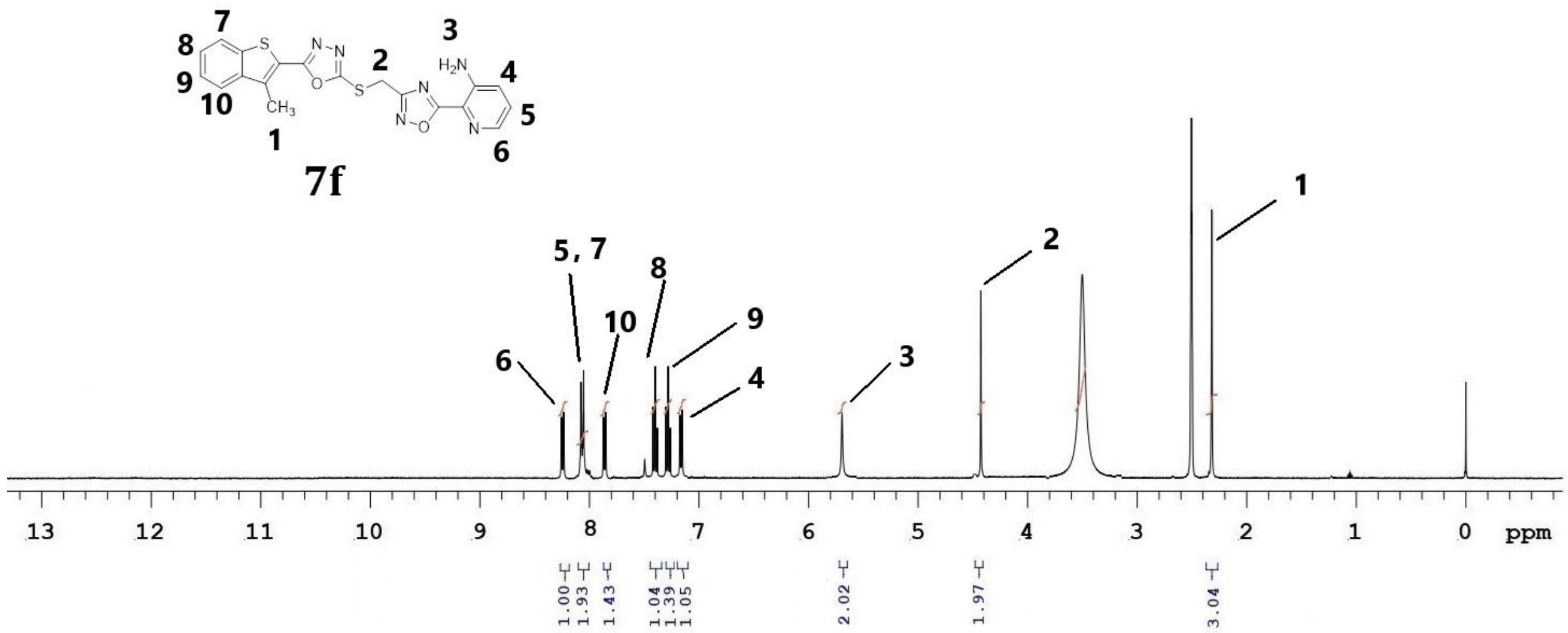

Figure S16. ^{1}H NMR of **7f**

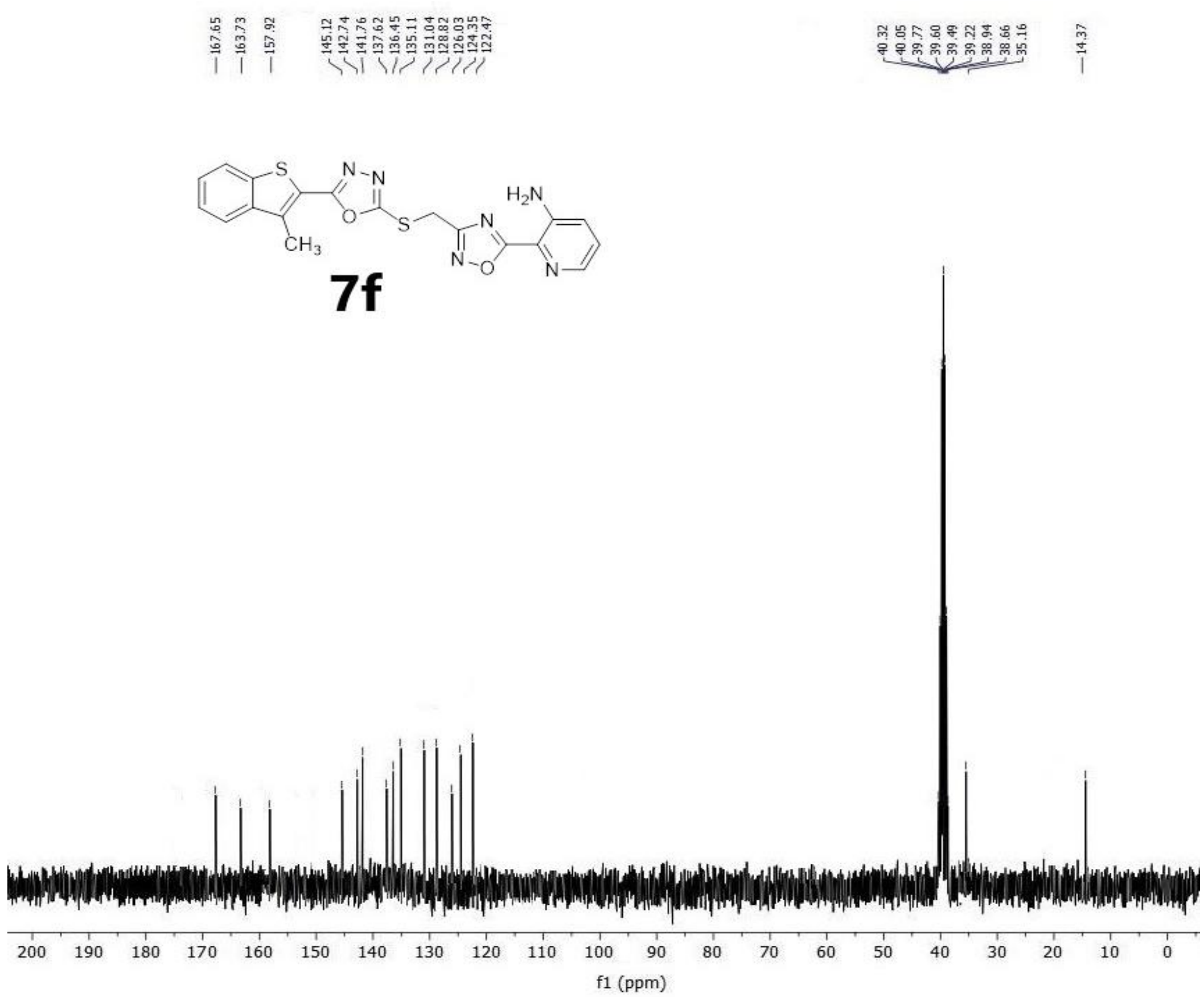

Figure S17. ^{13}C NMR of **7f**

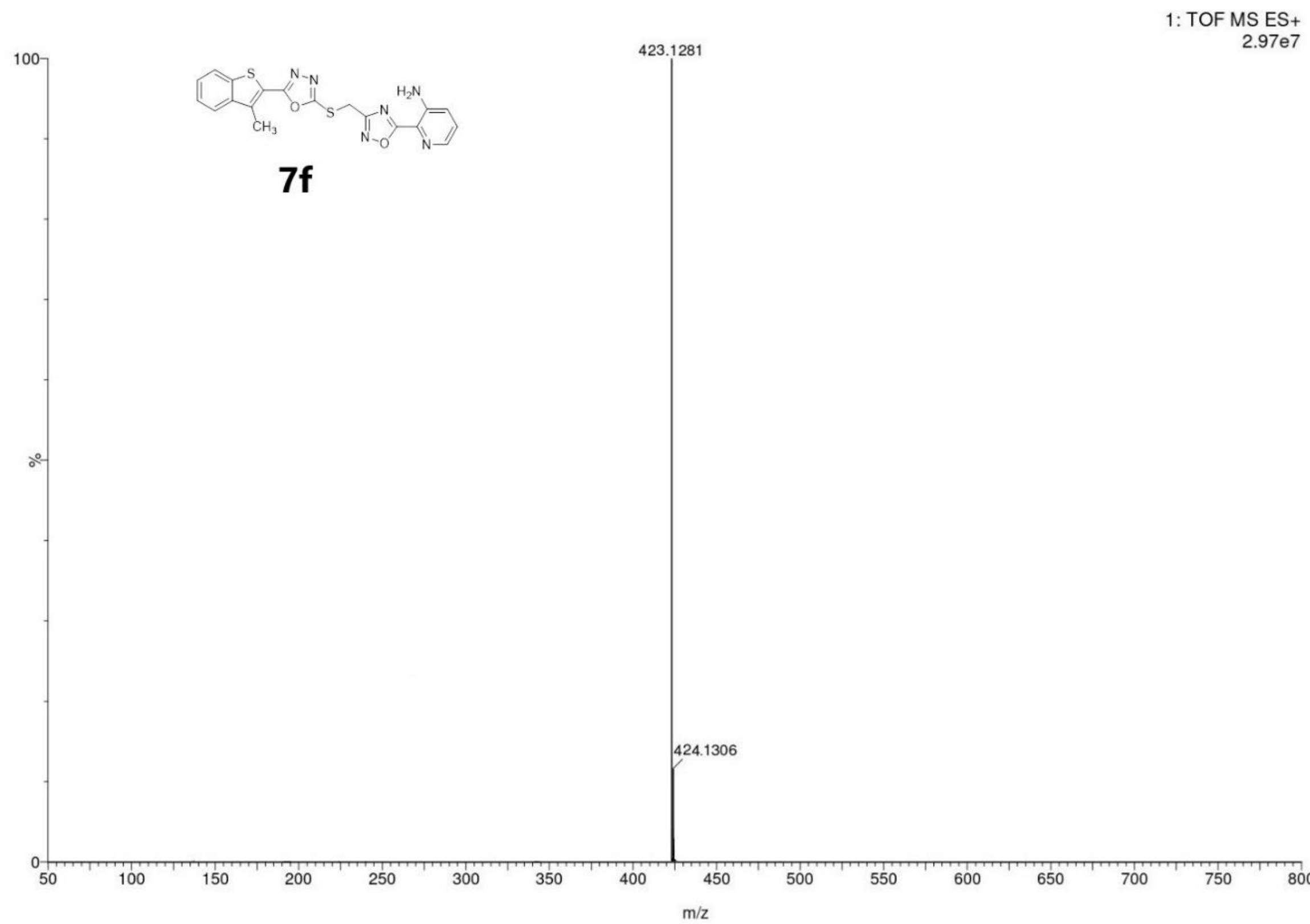

Figure S18. HRMS of **7f**

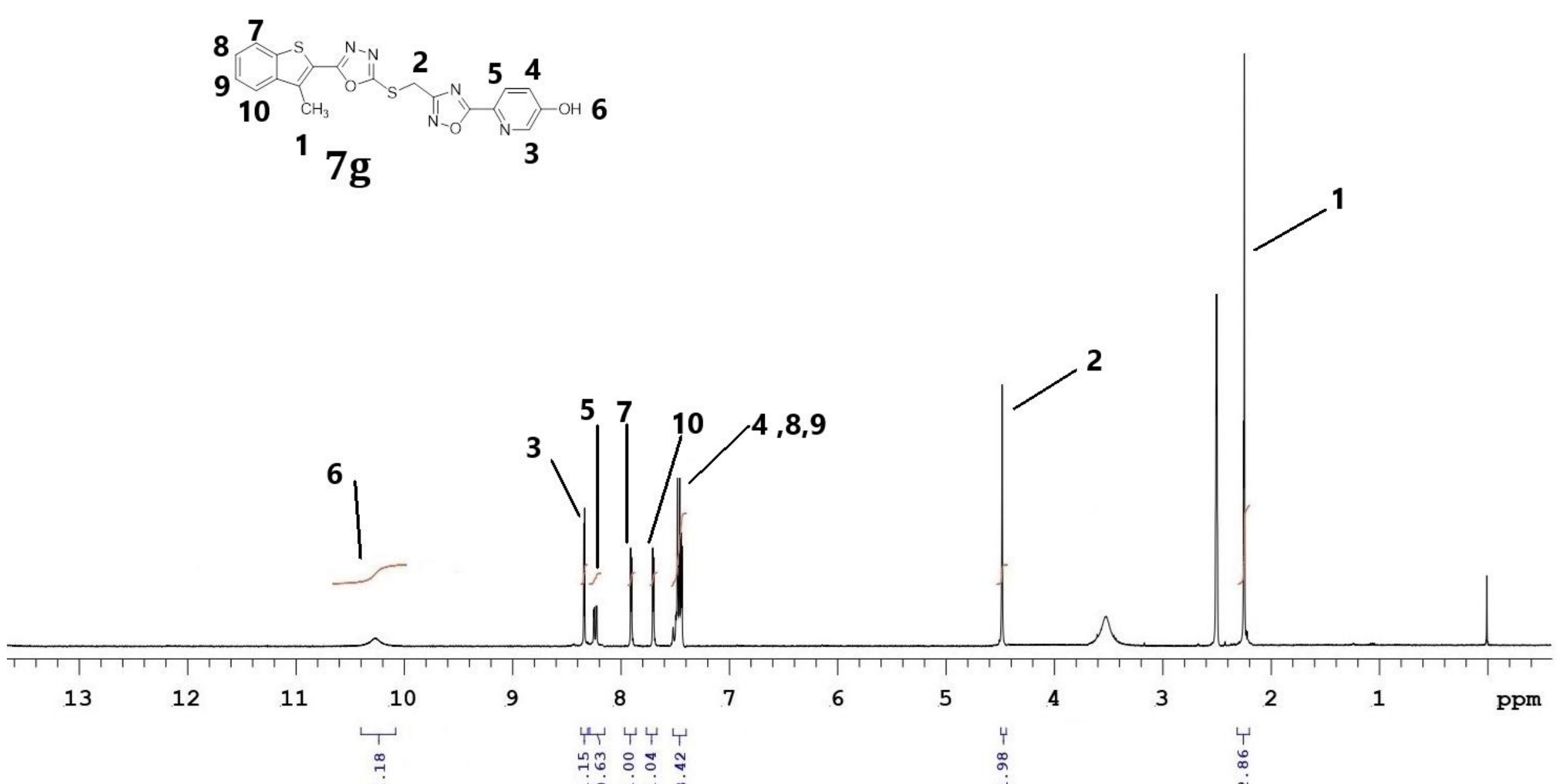

Figure S19. ^{1}H NMR of **7g**

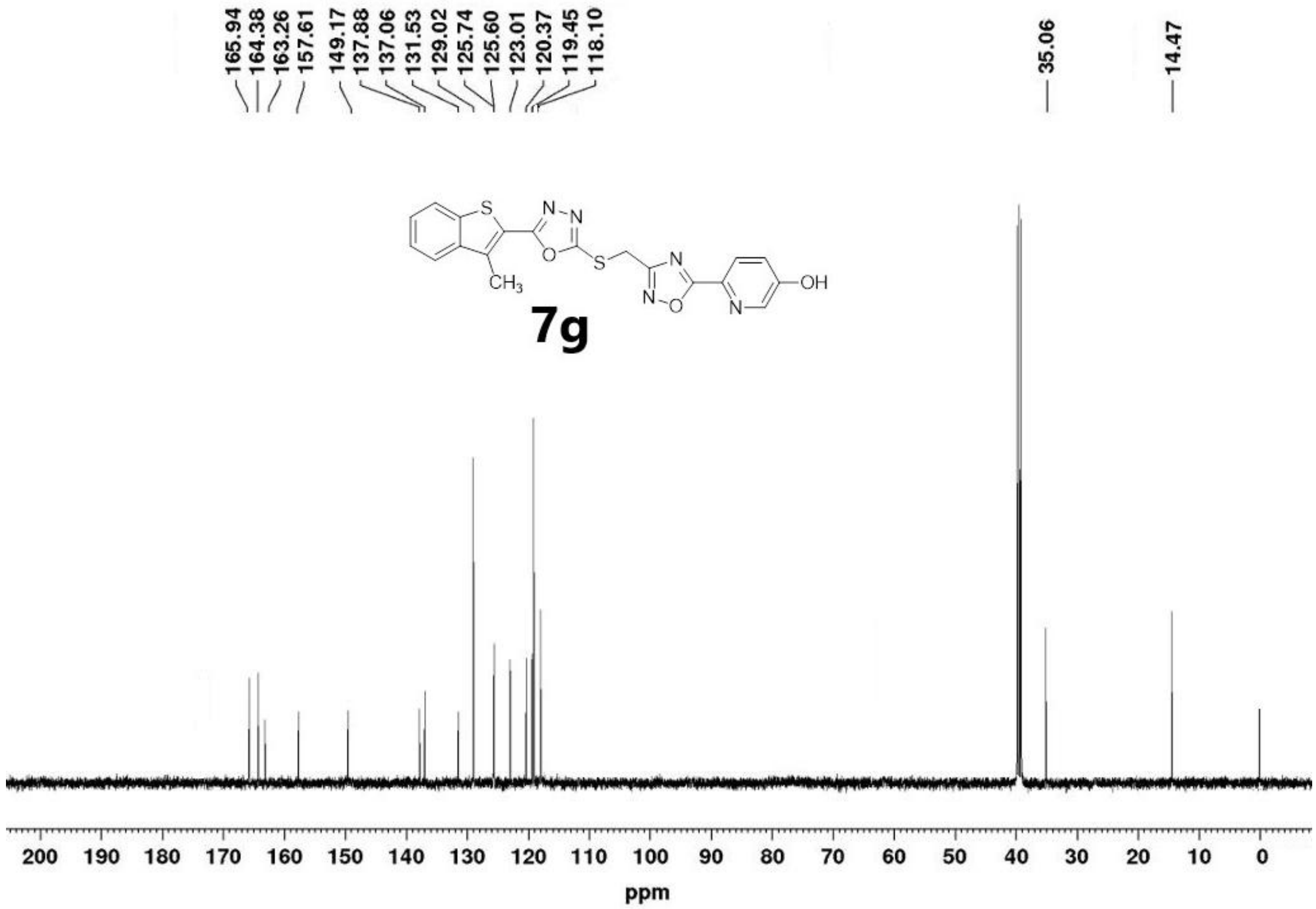

Figure S20. ^{13}C NMR of **7g**

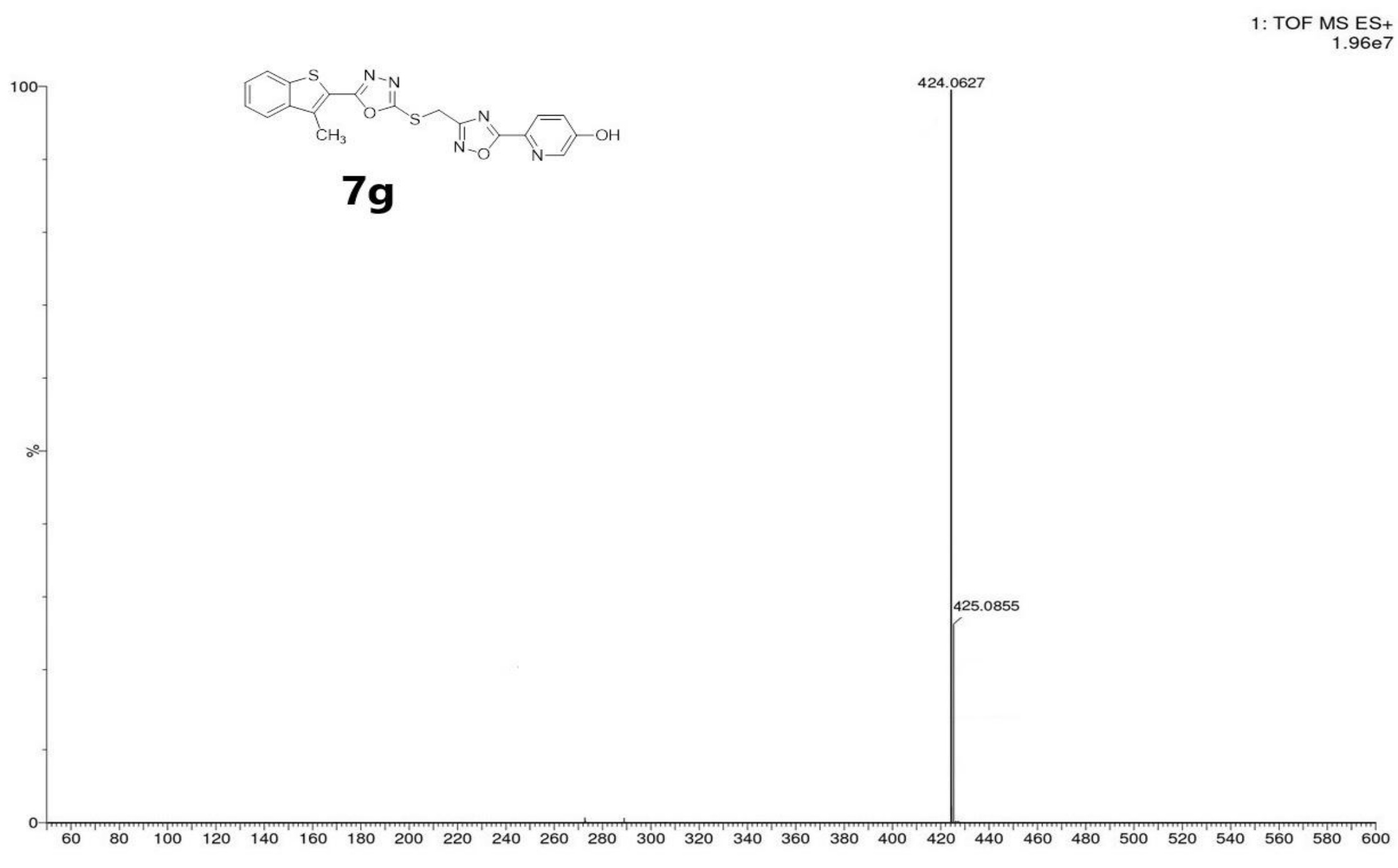

Figure S21. HRMS of **7g**

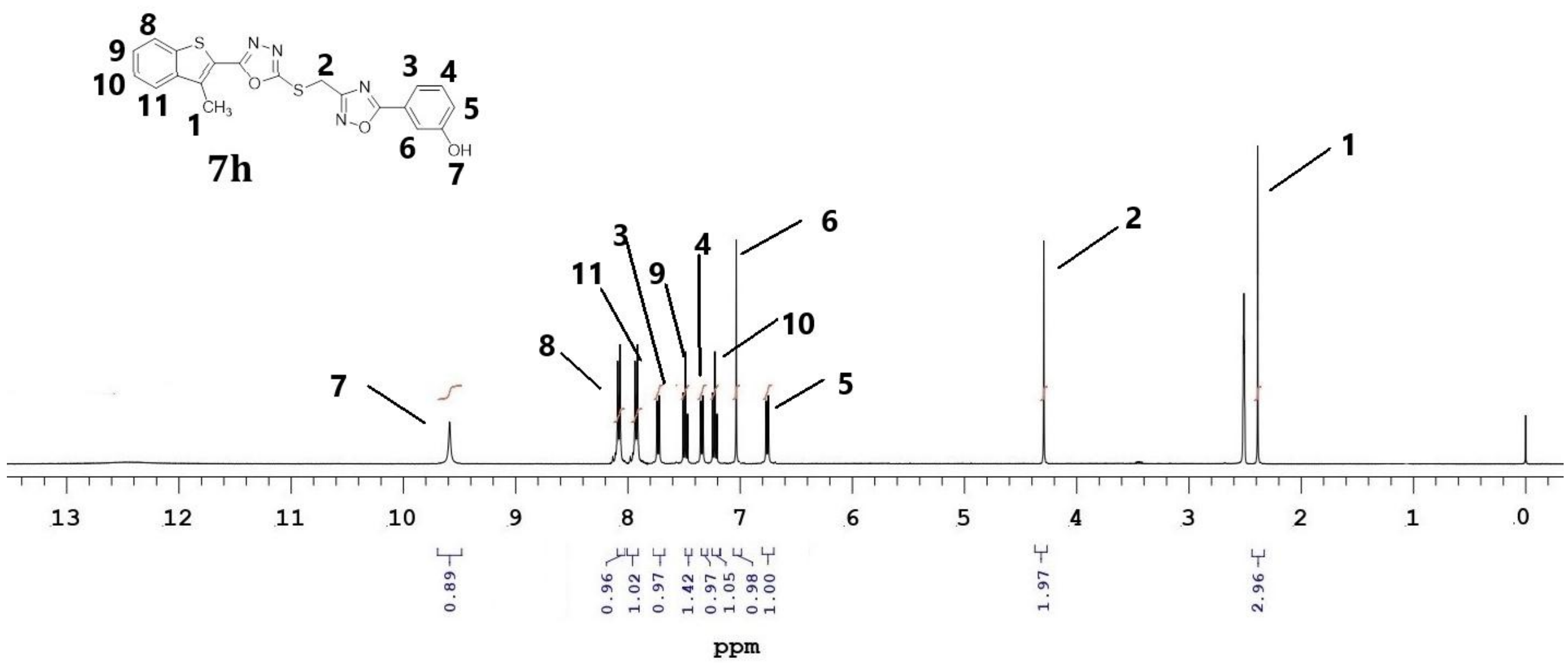

Figure S22. ^{1}H NMR of **7h**

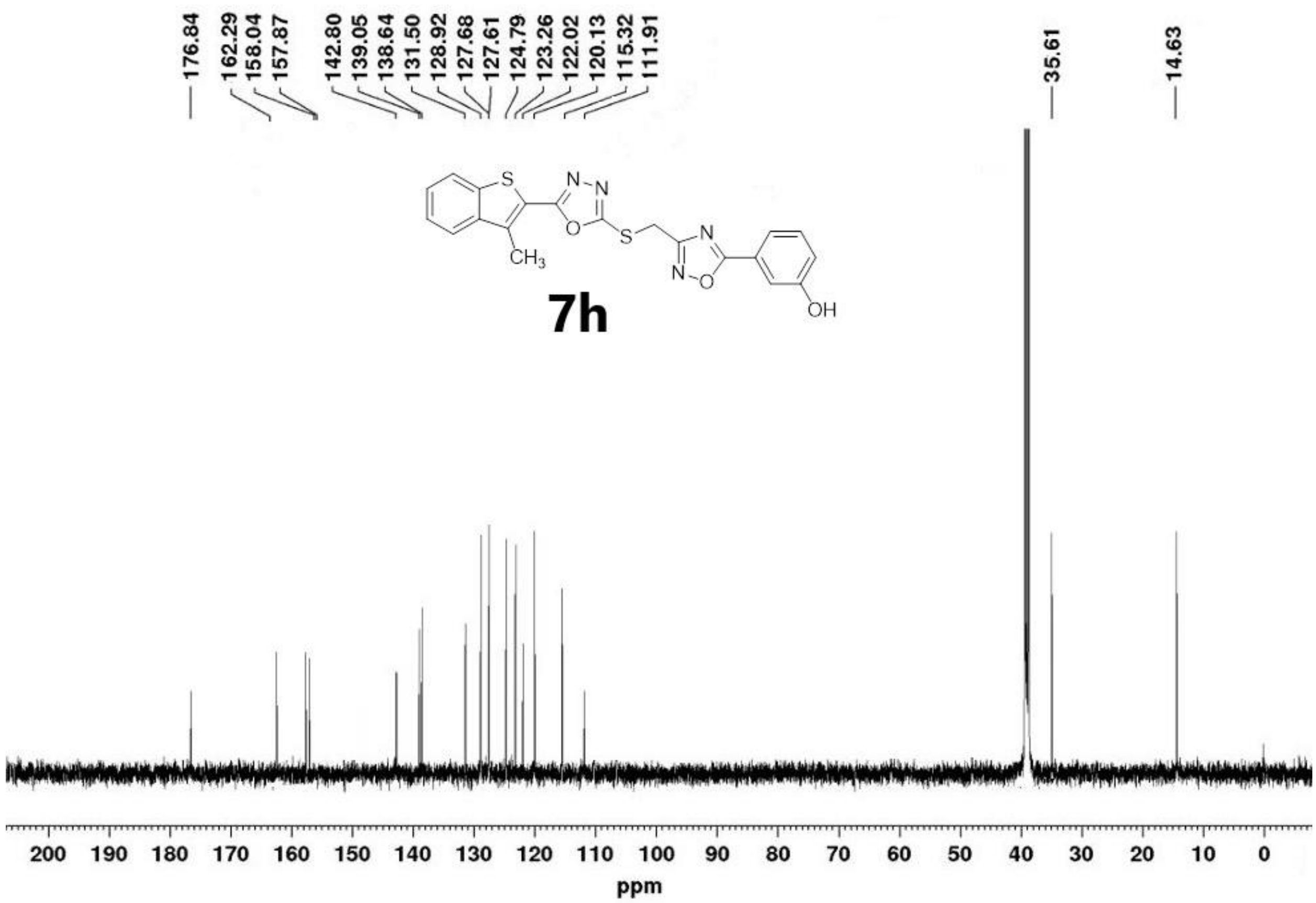

Figure S23. ^{13}C NMR of **7h**

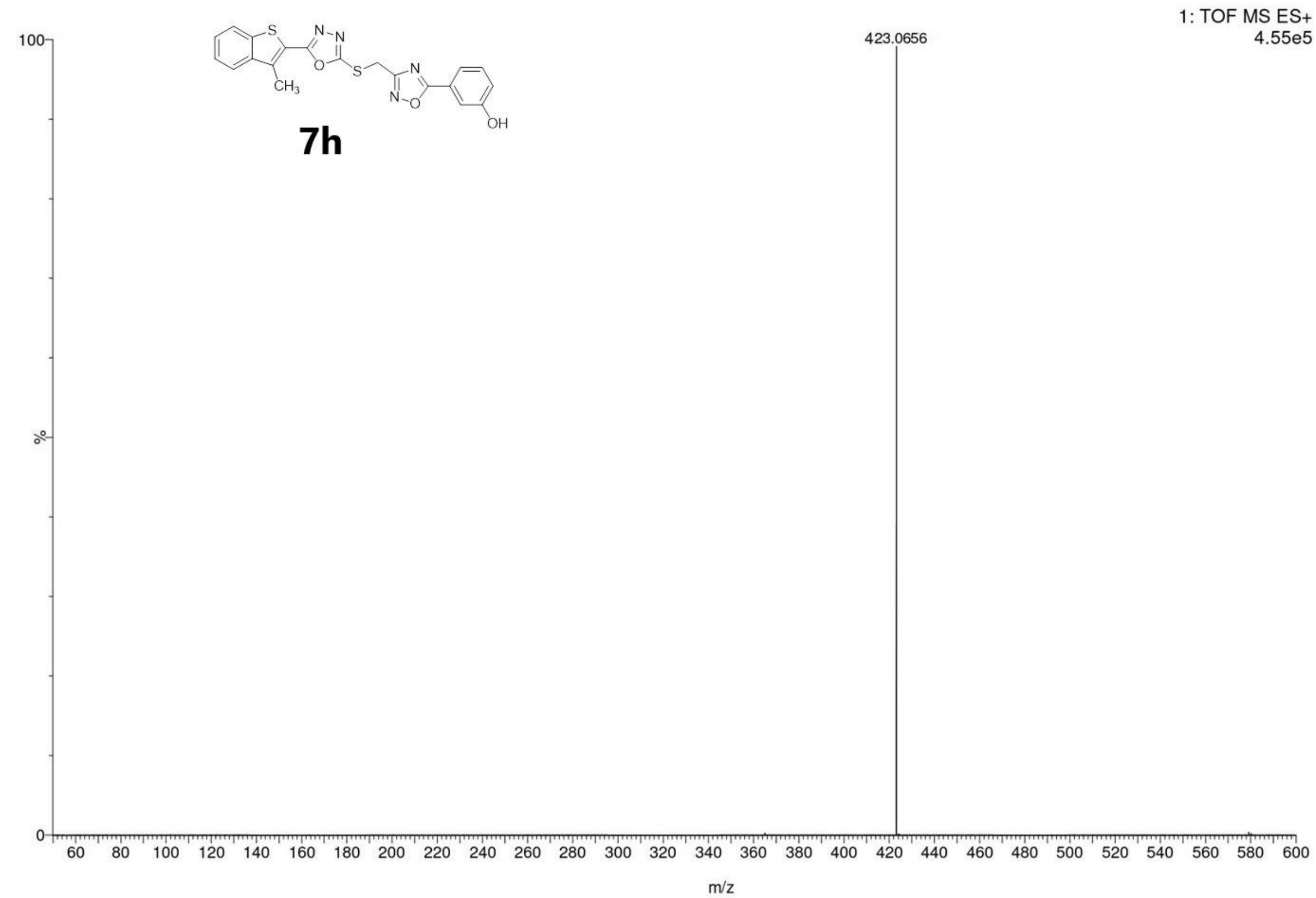

Figure S24. HRMS of **7h**

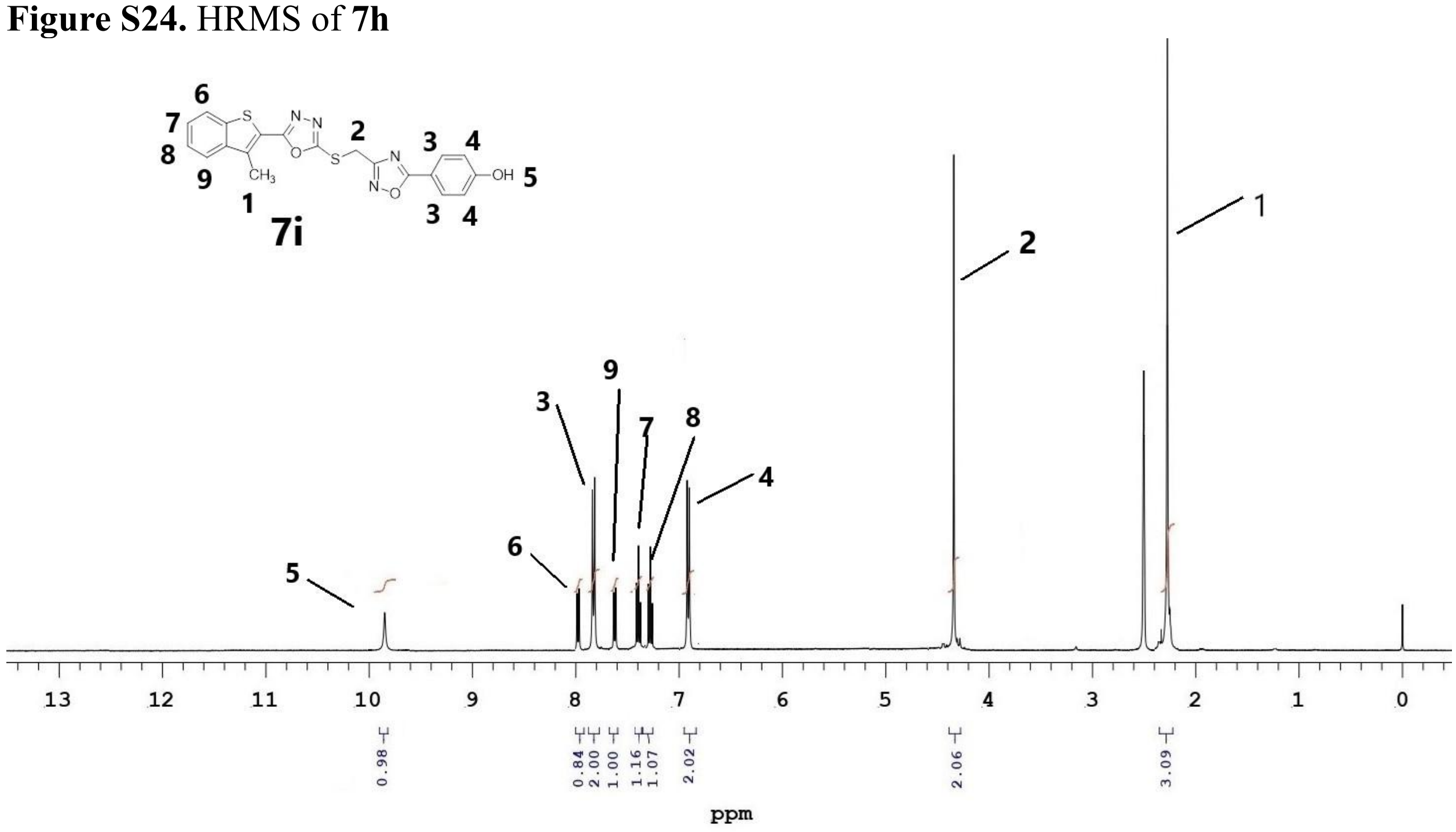

Figure S25. ^{1}H NMR of **7i**

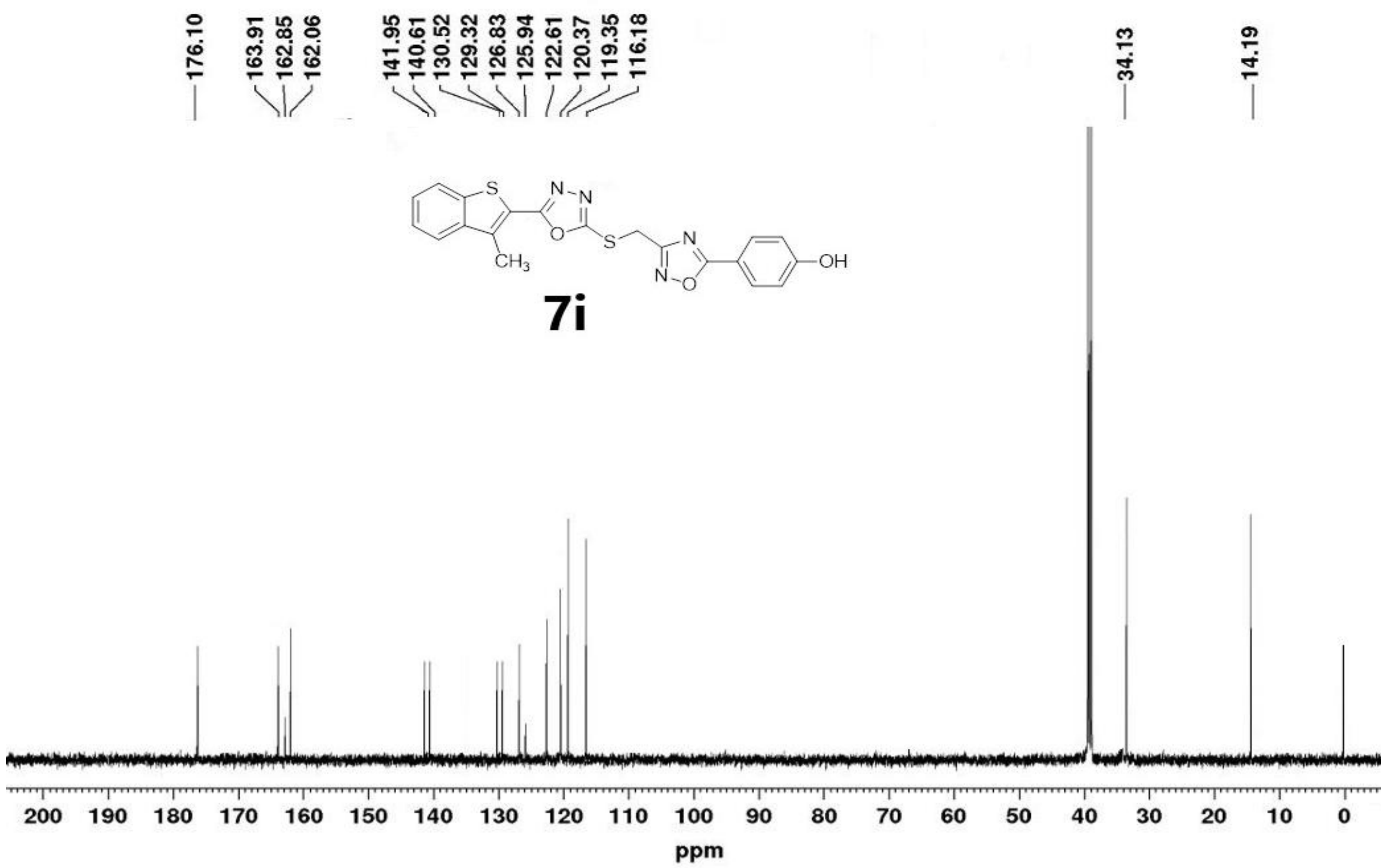

Figure S26. ^{13}C NMR of **7i**

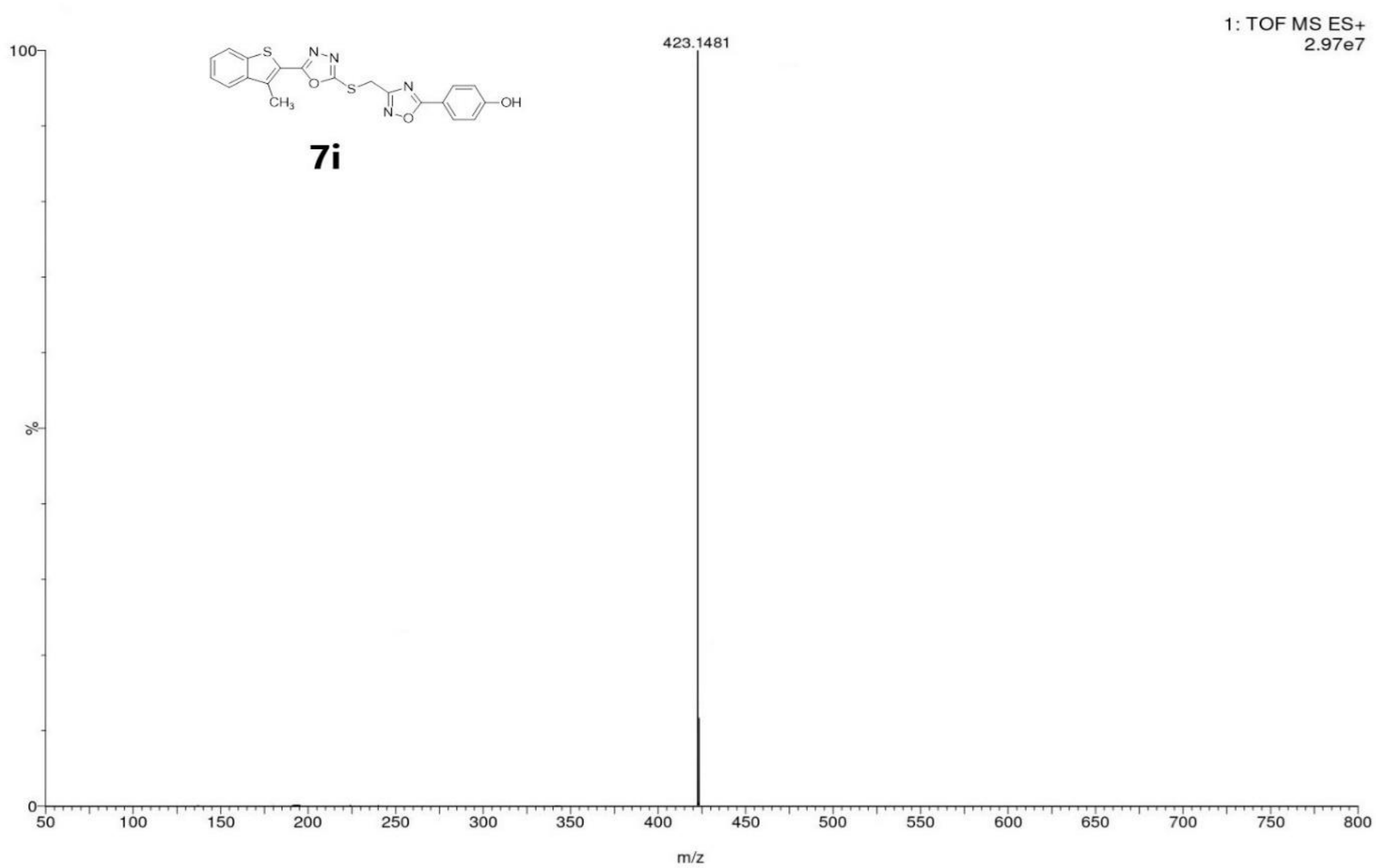

Figure S27. HRMS of **7i**

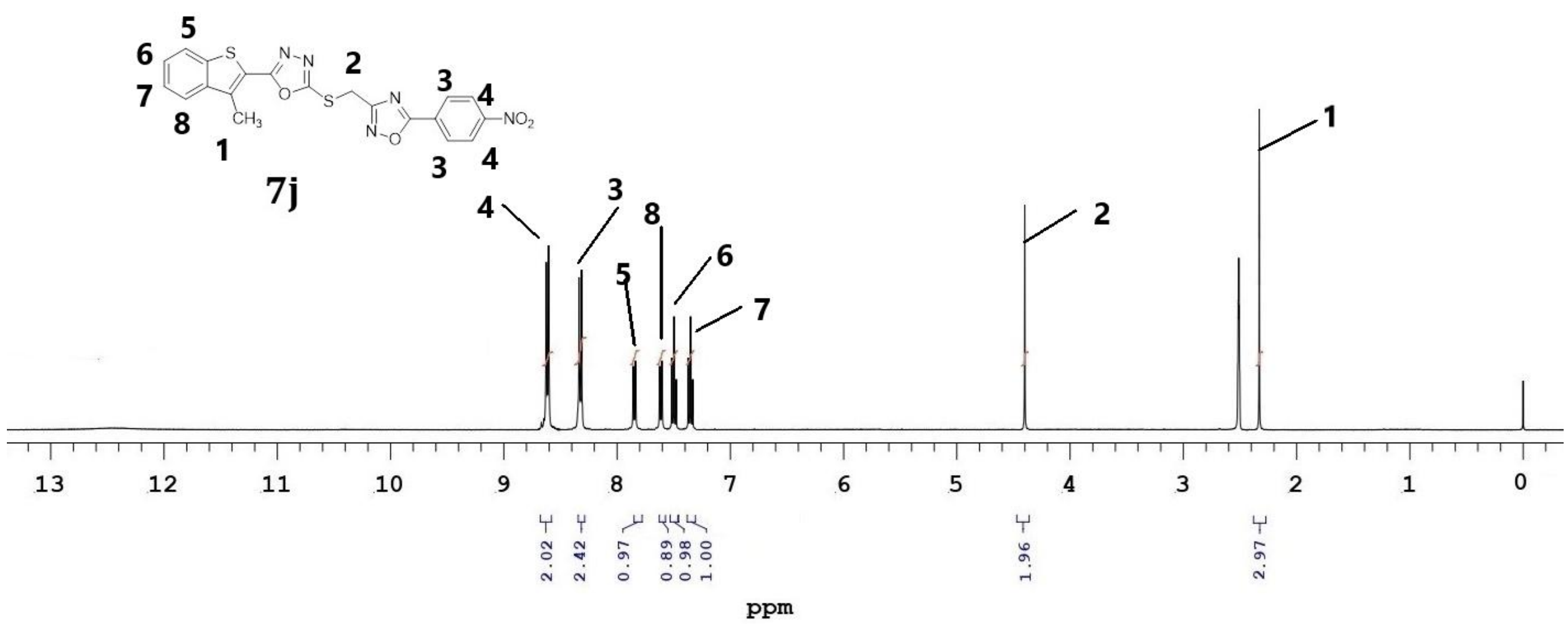

Figure S28. ^{1}H NMR of **7j**

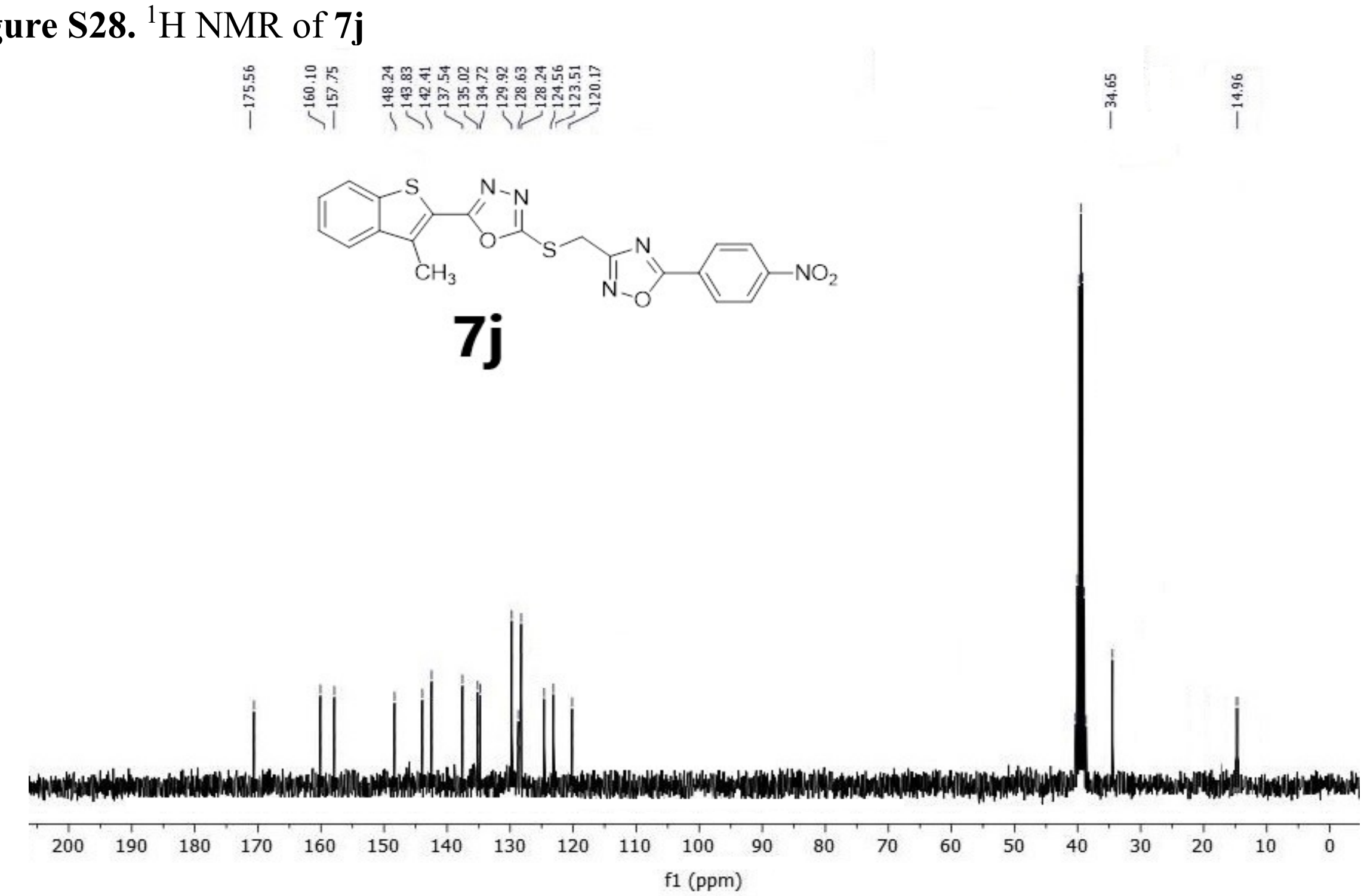

Figure S29. ^{13}C NMR of **7j**

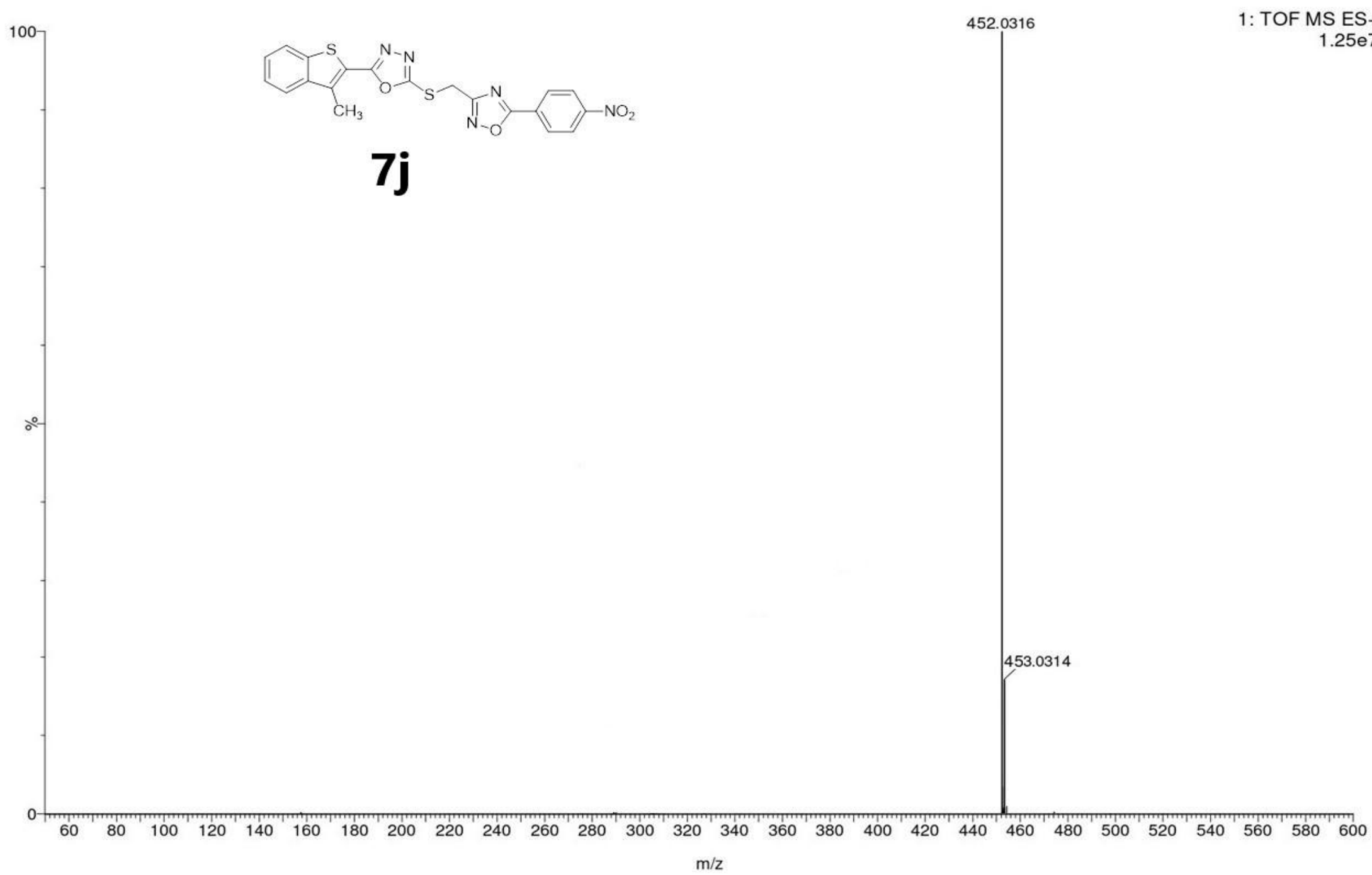

Figure S30. HRMS of **7j**

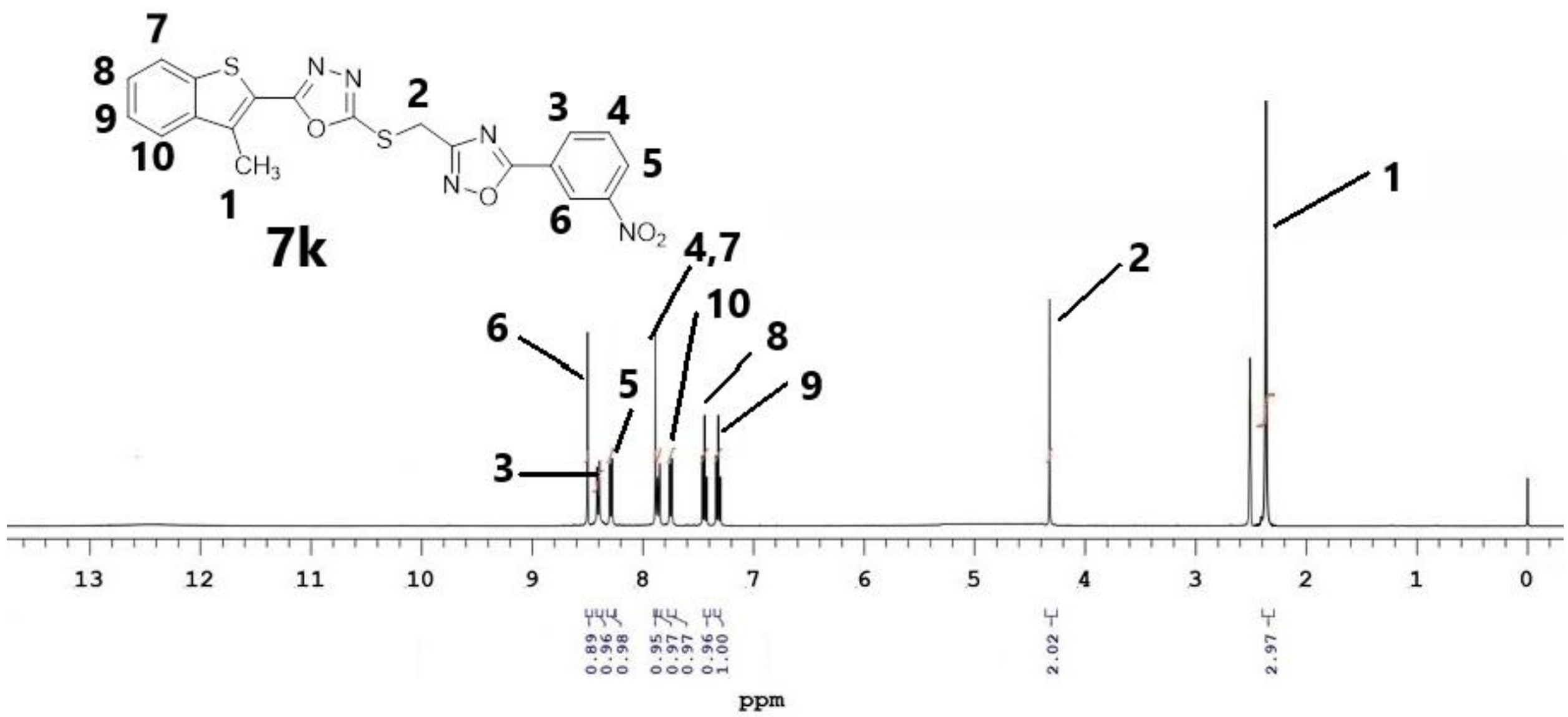

Figure S31. ^{1}H NMR of **7k**

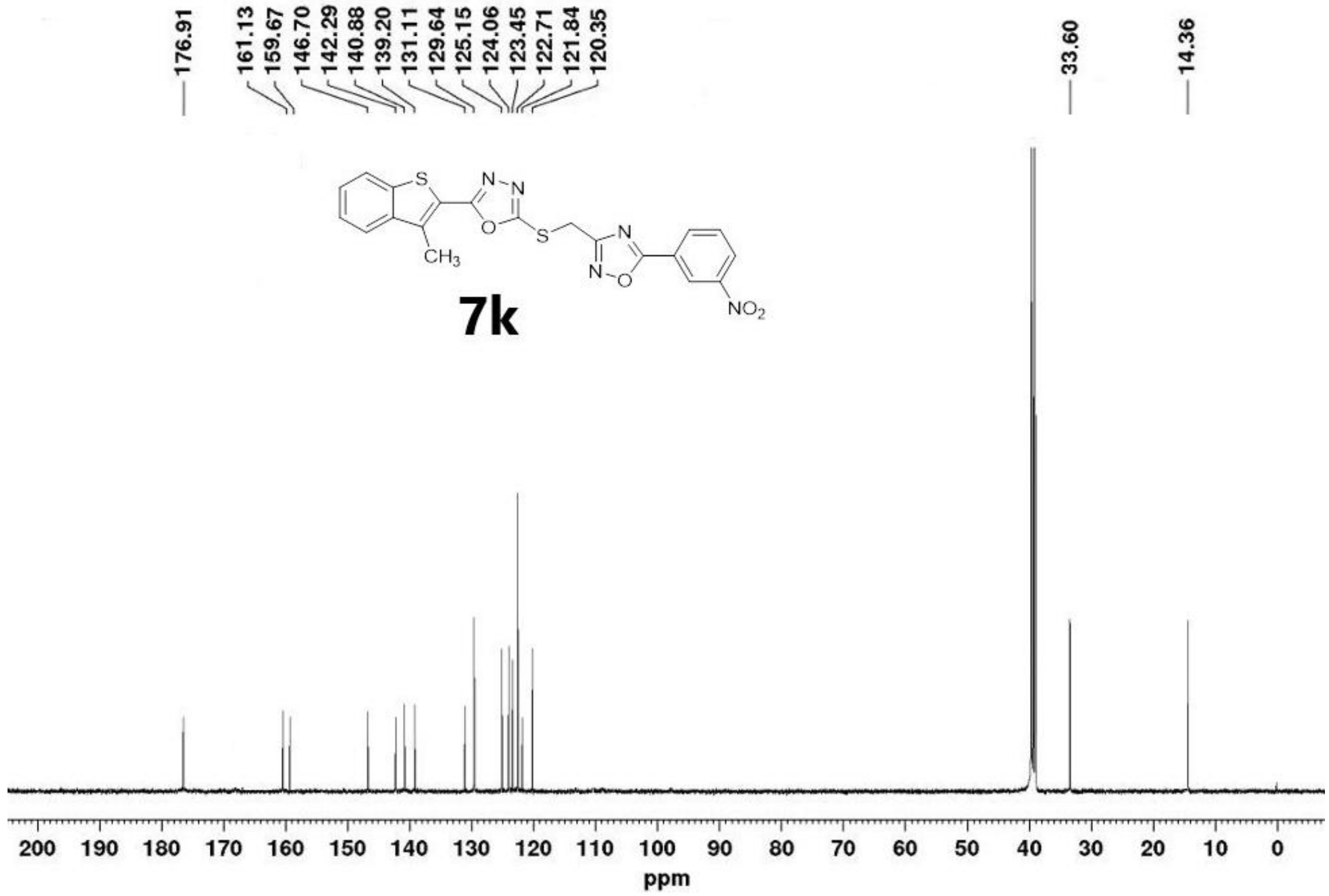

Figure S32. ^{13}C NMR of **7k**

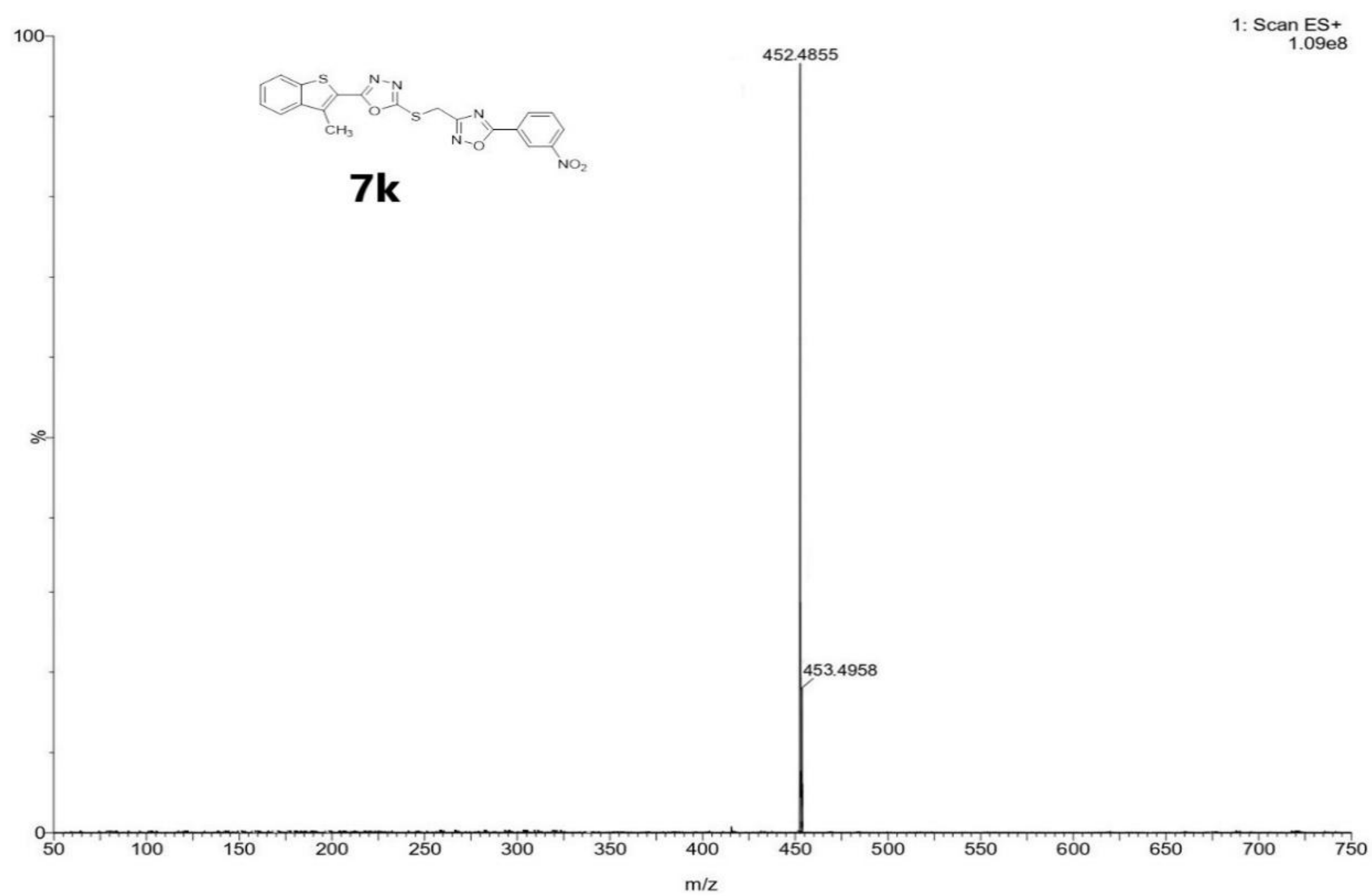

Figure S33. HRMS of **7k**
